# Identification of Genes Critical for Resistance to Infection by West Nile Virus Using RNA-Seq Analysis

**DOI:** 10.3390/v5071664

**Published:** 2013-07-08

**Authors:** Feng Qian, Lisa Chung, Wei Zheng, Vincent Bruno, Roger P. Alexander, Zhong Wang, Xiaomei Wang, Sebastian Kurscheid, Hongyu Zhao, Erol Fikrig, Mark Gerstein, Michael Snyder, Ruth R. Montgomery

**Affiliations:** 1 Section of Rheumatology, Department of Internal Medicine, Yale University School of Medicine, New Haven, CT 06520, USA; E-Mails: feng.qian@yale.edu (F.Q.); xiaomei.wang@yale.edu (X.W.); 2 W. M. Keck Biotechnology Resource Laboratory, Yale University School of Medicine, New Haven, CT 06520, USA; E-Mails: lisa.chung@yale.edu (L.C.); wei.zheng@yale.edu (W.Z.); hongyu.zhao@yale.edu (H.Z.); 3 Department of Biostatistics, Yale University School of Medicine, New Haven, CT 06520, USA; 4 Department of Molecular Biophysics and Biochemistry, Yale University, New Haven, CT 06520, USA; E-Mails: vbruno@som.umaryland.edu (V.B.); roger.alexander@yale.edu (R.P.A.); zhongwang@lbl.gov (Z.W.); mark.gerstein@yale.edu (M.G.); 5 Program in Computational Biology and Bioinformatics, Yale University, New Haven, CT 06520, USA; 6 Section of Infectious Diseases, Department of Internal Medicine, Yale University School of Medicine, New Haven, CT 06520, USA; E-Mail: sebastian.kurscheid@yale.edu (S.K.); erol.fikrig@yale.edu (E.F.); 7 The Howard Hughes Medical Institute, Chevy Chase, MD 20815, USA; 8 Department of Computer Science, Yale University, New Haven, CT 06520, USA; 9 Department of Genetics, Stanford University, Stanford, CA 94305, USA; E-Mail: mpsnyder@stanford.edu

**Keywords:** anti-viral gene expression, immune response, macrophage, RNA-Seq, West Nile virus

## Abstract

The West Nile virus (WNV) is an emerging infection of biodefense concern and there are no available treatments or vaccines. Here we used a high-throughput method based on a novel gene expression analysis, RNA-Seq, to give a global picture of differential gene expression by primary human macrophages of 10 healthy donors infected *in vitro* with WNV. From a total of 28 million reads per sample, we identified 1,514 transcripts that were differentially expressed after infection. Both predicted and novel gene changes were detected, as were gene isoforms, and while many of the genes were expressed by all donors, some were unique. Knock-down of genes not previously known to be associated with WNV resistance identified their critical role in control of viral infection. Our study distinguishes both common gene pathways as well as novel cellular responses. Such analyses will be valuable for translational studies of susceptible and resistant individuals—and for targeting therapeutics—in multiple biological settings.

## 1. Introduction

West Nile virus (WNV) is a mosquito-borne enveloped positive-strand RNA virus belonging to the family Flaviviridae, which includes yellow fever and dengue viruses [1]. From 1999–2012, 37,088 cases were reported to the Centers for Diseases Control, including 1,549 fatalities, with the estimated number of people infected approaching three million [2,3,4]. WNV infections in healthy humans are typically asymptomatic, but severe symptoms—more common in older patients (>55 years old)—including mengingoencephalitis and death [2,3]. The immune response to WNV is multifactorial including critical roles for both adaptive [5,6,7,8] and innate immune responses [9]. Mice depleted of macrophages, neutrophils, or lacking key components of innate immunity exhibit higher and extended viremia and increased mortality to infection with WNV [9,10,11]. 

The ability to profile the transcriptome of eukaryotic cells has led to the identification of key components that undergo alterations during diverse biological processes. For example, the profiling of gene expression in different leukemias has led to the classification of distinct subclasses of disease [12]. Recent advances in technology include an RNA sequencing method (RNA-Seq) that involves the direct sequencing of cDNA for monitoring quantitative gene expression [13,14,15,16,17,18,19,20]. RNA-Seq has at least an 8,000 fold dynamic range, and can follow expression at the level of an individual gene, exon and particular splice isoforms, eliminating the need for array design. Here we have used this method to identify the gene expression program of primary human immune cells in response to infection with WNV. We have identified both common and unique responses to viral infection as well as components linked to successful resistance to infection that may be targets for therapeutics. 

## 2. Results and Discussion

### 2.1. RNA-Seq Analysis of Differential Gene Expression by Human Macrophages Infected with WNV

To follow the level of expression of host genes that change during flaviviral infection, samples of primary human macrophages from 10 healthy donors were infected *in vitro* with virulent WNV (strain CT 2741, MOI = 1) for 24 hours [21]. Q-PCR confirmed elevated expression of mRNA for IL-8, expected in response to WNV infection, as well as of WNV E gene, reflecting the level of viral RNA (data not shown). PolyA+ RNA was prepared from uninfected and WNV-infected primary macrophages, fragmented, and subjected to sequencing using the Illumina Genome Analyzer 2. Approximately 28 million quality filtered 36 nt long reads were obtained from each sample. About 88% of reads were mapped to the human genome using TOPHAT [22], suggesting good quality of RNA-seq (Supplementary Table S1). Genes and transcripts were scored for expression by a maximum likelihood based method implemented in Cufflinks [23]. 

**Figure 1:**
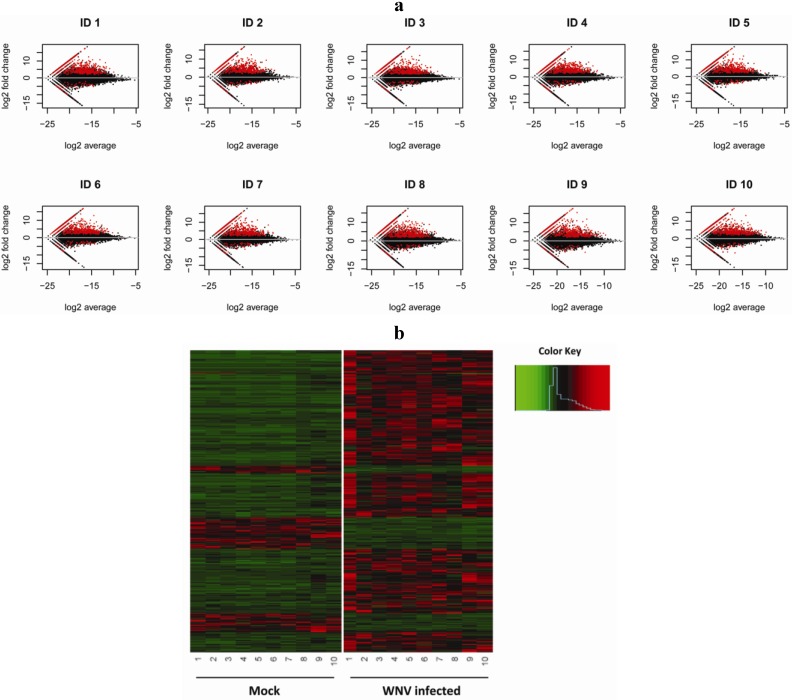
Differential gene expression of human macrophages infected with West Nile virus (WNV). (**a**) MA plots of RNA-seq data. The M (log fold change) of each transcript between mock and infected pair is plotted against A (average log concentration/expression level) of each mock and infected pair. In these plots, each point represents an annotated transcript. The black dots reflect no change and the red dots represent transcripts with 4-fold change by edgeR analysis. The straight lines in each plot reflect zero expression in one condition and nonzero expression in the other condition. (**b**) Heatmap for 1,514 differentially expressed transcripts (Bayesian DE paired analysis) in human macrophages infected with WNV using hierarchical clustering analysis. Red, black and green colors indicate gene expression above, equal to and below the mean, respectively, for subjects #1–10.

Variation between human subjects may reveal distinct yet effective anti-viral mechanisms used by different subjects when responding to infection with WNV. Thus we sought to identify both common and individual-specific changes in the anti-viral gene program. We compared the normalized expression levels from control uninfected and WNV-infected macrophage by performing both pairwise and joint comparisons to detect differentially expressed transcripts. Both methods identified a consistent set of differentially expressed genes. The first method compares each control/infected sample pair using fold change adjusted by using trimmed-mean normalization [24]. A total of 732 transcripts showed 4-fold or greater change after infection with WNV consistently across all 10 individuals (Supplementary Table S2). When comparing the fold change *vs.* the expression level for our 10 biological uninfected and infected sample pairs, we noted both upregulation and downregulation of transcripts that reached greater than 4 fold (Figure 1a). The pattern of differential expression of transcripts was similar across all 10 donors suggesting that the anti-viral program includes an essential cluster of regulated genes. These include well-characterized responses such as type I interferons and chemokines and chemokine receptors [25] as well as less well characterized targets. 

Of note, individuals may have unique mechanisms for anti-viral responses and our initial analysis would not identify differentially expressed transcripts detected for some individuals but not others. To achieve higher statistical power in analysis of our samples, we combined information from subject samples simultaneously and compared the 10 control/10 infected sample pairs jointly using a Bayesian hierarchical mixture model to identify differentially expressed transcripts [26]. This newly developed algorithm adopts a Poisson-Gamma model for paired expression counts to account for individual variation. The expression difference (fold change) is modeled by a mixture of two component distributions, one for equal expression and the other for differential expression. Parameter space and posterior probability of being differentially expressed are explored using Markov Chain Monte-Carlo (MCMC). When this approach was applied to the full collection of 10 sample pairs using the posterior probability cutoff 0.5, we detected 1,514 transcripts with differential expression between the control uninfected and WNV-infected samples (Supplementary Table S3). This collection of transcripts identified includes 505 of the 732 transcripts detected by the individual pair analysis and a wealth of additional transcripts (1,009) that may provide critical insight into anti-viral pathways. For a global perspective on gene regulation during WNV infection, a heat map of 1,514 differentially expressed transcripts was generated using hierarchical clustering analysis (Figure 1b). Our approach identifies differentially expressed genes at a lower false discovery rate and successfully detects transcripts with high differences after infection even if overall expression levels are low. The Bayesian analysis can also identify a transcript that shows a large difference across a majority of individuals while a few individuals show no difference. The magnitude of the fold change can differ individual by individual. This paired analysis allows sufficient analytic depth to identify transcriptional changes that may represent an uncommon anti-viral response pathway, which can be subjected to additional studies to clarify pathogenetic mechanisms.

In addition to identifying differentially expressed transcripts, the depth of the RNA-seq datasets can identify changes at the isoform/splicing level of individual genes [19]. In this analysis, we have detected 1,514 transcripts corresponding to 1,148 unique genes of which 366 (23%) were found to have >1 isoform. This powerful analysis demonstrates the pattern of over or under expression for both consistent and unique features of the macrophage anti-viral program.

### 2.2. Virally Induced Pathways Identified by Functional Annotation Clustering

To determine the cellular effects of these differentially expressed gene transcripts, we conducted functional annotation clustering analysis using DAVID, an on-line functional annotation tool for gene enrichment analysis [27,28]. The list of ENSEMBL transcript IDs corresponding to these differentially expressed genes mapped to 929 DAVID IDs [29]. The functional annotation analysis of the top 8 clusters, all with an enrichment score ≥5.24, reflects an active anti‑viral gene expression program including the genes that were detected to be commonly up- or down-regulated in response to *in vitro* WNV-challenge (Table 1). Cluster 1 (DAVID enrichment score: 20.92) primarily consists of genes that are involved in the regulation of defense and immune response. The second functional annotation cluster (DAVID enrichment score: 9.21) includes genes related to immune cell activation, cluster 3 (DAVID enrichment score: 6.62) and cluster 4 (DAVID enrichment score: 6.40) consists of genes with known functions in cytokine and chemokine production. Additional clusters 5–8 identify molecular functions of genes associated with a more generalized apoptosis, cell death, and signal transduction. The induction of apoptosis by WNV, which regulates pro-inflammatory responses, has been previously reported in cell lines and neuronal cell types and may be mediated by induction of regulatory miRNA [30,31,32]. The functional cluster analysis was similar when we analyzed the 1,009 transcripts identified only using the Bayesian method; these were enriched to immune response categories reflecting an active anti-viral gene expression program (data not shown). 

While the majority of differentially expressed genes showed an increase on infection with WNV, 293 of the 1,514 transcripts were detected to be down-regulated (19.4%). The down-regulated transcripts were enriched in functional clusters involved in intracellular organelle and membrane transport functions in the samples infected with WNV. Notably, the sensitivity provided by the model also allows identification of rare differentially expressed transcripts in only a few donors for which determining functional significance will require further investigation. 

**Table 1:** Results of functional annotation clustering.

Category	Term	Count	PValue	FDR
**Cluster 1**	**Enrichment Score: 20.92**			
GO: BP	GO:0006952~defense response	101	2.50 × 10^−25^	4.53 × 10^−22^
GO: BP	GO:0006954~inflammatory response	64	3.66 × 10^−20^	6.64 × 10^−17^
GO: BP	GO:0009611~response to wounding	83	1.80 × 10^−19^	3.27 × 10^−16^
**Cluster 2**	**Enrichment Score: 9.21**			
GO: BP	GO:0001775~cell activation	46	2.83 × 10^−11^	5.13 × 10^−8^
GO: BP	GO:0045321~leukocyte activation	40	2.67 × 10^−10^	4.84 × 10^−7^
GO: BP	GO:0046649~lymphocyte activation	34	3.07 × 10^−9^	5.57 × 10^−6^
GO: BP	GO:0042110~T cell activation	26	6.22 × 10^−9^	1.13 × 10^−5^
**Cluster 3**	**Enrichment Score: 6.62**			
SP_PIR_KEYWORDS	inflammatory response	26	4.45 × 10^−15^	6.43 × 10^−12^
SP_PIR_KEYWORDS	chemotaxis	22	7.21 × 10^−12^	1.04 × 10^−8^
GO: MF	GO:0005125~cytokine activity	37	2.14 × 10^−11^	3.31 × 10^−8^
SP_PIR_KEYWORDS	cytokine	33	7.05 × 10^−11^	1.02 × 10^−7^
GO: MF	GO:0042379~chemokine receptor binding	18	1.62 × 10^−10^	2.50 × 10^−7^
INTERPRO	IPR001811:Small chemokine, interleukin-8-like	16	3.86 × 10^−10^	6.24 × 10^−7^
GO: MF	GO:0008009~chemokine activity	17	5.38 × 10^−10^	8.32 × 10^−7^
SMART	SM00199:SCY	16	2.96 × 10^−9^	3.89 × 10^−6^
KEGG_PATHWAY	hsa04060:Cytokine-cytokine receptor interaction	44	4.30 × 10^−9^	5.16 × 10^−6^
SP_PIR_KEYWORDS	inflammation	12	5.28 × 10^−9^	7.64 × 10^−6^
GO: BP	GO:0006935~chemotaxis	27	2.20 × 10^−7^	3.99 × 10^−4^
GO: BP	GO:0042330~taxis	27	2.20 × 10^−7^	3.99 × 10^−4^
GO: CC	GO:0005615~extracellular space	65	2.68 × 10^−7^	3.73 × 10^−4^
KEGG_PATHWAY	hsa04062:Chemokine signaling pathway	32	5.92 × 10^−7^	7.10 × 10^−4^
GO: BP	GO:0007626~locomotory behavior	35	2.35 × 10^−6^	4.26 × 10^−3^
PIR_SUPERFAMILY	PIRSF001950:small inducible chemokine, C/CC types	9	1.28 × 10^−5^	1.78 × 10^−2^
INTERPRO	IPR000827:Small chemokine, C-C group, conserved site	9	1.66 × 10^−5^	2.68 × 10^−2^
**Cluster 4**	**Enrichment Score: 6.40**			
GO: BP	GO:0001817~regulation of cytokine production	33	1.03 × 10^−9^	1.86 × 10^−6^
GO: BP	GO:0051240~positive regulation of multicellular organismal process	32	3.97 × 10^−6^	7.21 × 10^−3^
GO: BP	GO:0001819~positive regulation of cytokine production	17	1.47 × 10^−5^	2.67 × 10^−2^
**Cluster 5**	**Enrichment Score: 6.32**			
GO: BP	GO:0002237~response to molecule of bacterial origin	20	6.81 × 10^−8^	1.24 × 10^−4^
GO: BP	GO:0034097~response to cytokine stimulus	19	9.11 × 10^−8^	1.65 × 10^−4^
GO: BP	GO:0032496~response to lipopolysaccharide	18	3.37 × 10^−7^	6.11 × 10^−4^
GO: BP	GO:0009617~response to bacterium	26	2.43 × 10^−5^	4.41 × 10^−2^
**Cluster 6**	**Enrichment Score: 5.95**			
GO: BP	GO:0042981~regulation of apoptosis	87	1.03 × 10^−10^	1.86 × 10^−7^
GO: BP	GO:0043067~regulation of programmed cell death	87	1.65 × 10^−10^	3.00 × 10^−7^
GO: BP	GO:0010941~regulation of cell death	87	1.99 × 10^−10^	3.61 × 10^−7^
GO: BP	GO:0043065~positive regulation of apoptosis	48	1.20 × 10^−6^	2.18 × 10^−3^
GO: BP	GO:0043068~positive regulation of programmed cell death	48	1.46 × 10^−6^	2.65 × 10^−3^
GO: BP	GO:0010942~positive regulation of cell death	48	1.67 × 10^−6^	3.03 × 10^−3^
GO: BP	GO:0006916~anti-apoptosis	27	2.66 × 10^−5^	4.82 × 10^−2^
GO: BP	GO:0006917~induction of apoptosis	36	2.75 × 10^−5^	4.98 × 10^−2^
**Cluster 7**	**Enrichment Score: 5.33**			
GO: BP	GO:0002684~positive regulation of immune system process	42	1.05 × 10^−11^	1.91 × 10^−8^
GO: BP	GO:0050865~regulation of cell activation	29	1.06 × 10^−7^	1.92 × 10^−4^
GO: BP	GO:0002694~regulation of leukocyte activation	28	1.26 × 10^−7^	2.29 × 10^−4^
GO: BP	GO:0051249~regulation of lymphocyte activation	26	1.75 × 10^−7^	3.17 × 10^−4^
GO: BP	GO:0050671~positive regulation of lymphocyte proliferation	15	5.79 × 10^−7^	1.05 × 10^−3^
GO: BP	GO:0032946~positive regulation of mononuclear cell proliferation	15	7.35 × 10^−7^	1.33 × 10^−3^
GO: BP	GO:0070665~positive regulation of leukocyte proliferation	15	7.35 × 10^−7^	1.33 × 10^−3^
GO: BP	GO:0050867~positive regulation of cell activation	21	1.04 × 10^−6^	1.88 × 10^−3^
GO: BP	GO:0050670~regulation of lymphocyte proliferation	18	1.04 × 10^−6^	1.89 × 10^−3^
GO: BP	GO:0032944~regulation of mononuclear cell proliferation	18	1.24 × 10^−6^	2.26 × 10^−3^
GO: BP	GO:0070663~regulation of leukocyte proliferation	18	1.24 × 10^−6^	2.26 × 10^−3^
GO: BP	GO:0002696~positive regulation of leukocyte activation	20	2.08 × 10^−6^	3.78 × 10^−3^
GO: BP	GO:0051251~positive regulation of lymphocyte activation	19	2.30 × 10^−6^	4.17 × 10^−3^
GO: BP	GO:0050863~regulation of T cell activation	20	9.40 × 10^−6^	1.71 × 10^−2^
**Cluster 8**	**Enrichment Score: 5.24**			
GO: BP	GO:0043122~regulation of I-kappaB kinase/NF-kappaB cascade	21	5.63 × 10^−7^	1.02 × 10^−3^
GO: BP	GO:0043123~positive regulation of I-kappaB kinase/NF-kappaB cascade	19	2.30 × 10^−6^	4.17 × 10^−3^
GO: BP	GO:0010647~positive regulation of cell communication	39	3.50 × 10^−6^	6.36 × 10^−3^
GO: BP	GO:0010740~positive regulation of protein kinase cascade	25	5.99 × 10^−6^	1.09 × 10^−2^

### 2.3. Correlation of Gene Expression Changes with Genome-Wide RNAi Analysis

**Figure 2:**
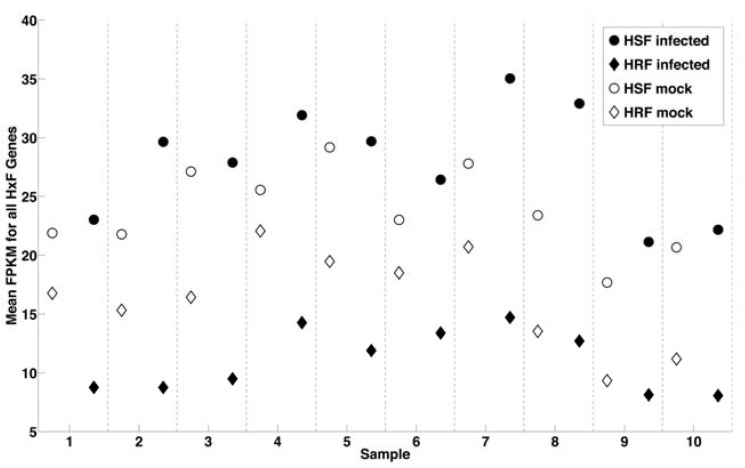
Correlation of WNV infection with change in expression of genes identified by genome-wide RNAi screen. Plot represents average expression (FPKM) of ~180 host susceptibility factors (HSF) genes in mock (●) and ~13 host resistance factors (HRF) genes (◆) samples with uninfected samples shown in open symbols. For all 10 samples, the average expression (FPKM) of HSF genes increases while the average expression of HRF genes decreases from mock to infected samples.

We have further classified our results from RNA-Seq in light of our recent genome-scale RNA interference (RNAi) screen for human genes associated with WNV infection [33]. This high‑throughput study silenced 21,121 human genes in HeLa cells and provided the first comprehensive picture of the molecular interactions of WNV with human cells. RNAi has proven informative for studies of HIV and hepatitis C virus as well [34,35]. RNAi identified human cellular factors facilitating WNV infection including several known host factors of WNV infection (e.g., endosomal proton pump vATPase) and 294 host targets that have not previously been associated with WNV infection, defined as host susceptibility factors (HSF) and host resistance factors (HRF) [33]. When we calculated the average expression in each RNA-Seq sample of ~200 HSF and ~13 HRF as defined in the RNAi study, we found that for each subject, average HSF expression increases and average HRF expression decreases after infection (Figure 2). Further, we identified 13 HSFs and 2 HRFs from HeLa cells that are also differentially expressed in RNA-Seq analysis of human macrophages and likely represent important elements in the anti-viral gene program (Supplementary Table S4). While the magnitude of effect varied between donors, the overall gene changes are quite consistent. This suggests that viral infection is a powerful stimulus that may redirect host gene expression to create an environment that is more conducive to the virus, a common viral strategy to promote replication and survival.

### 2.4. RNAi Knockdown of Novel Genes Shows Critical Role in Resistance to WNV Infection

**Figure 3:**
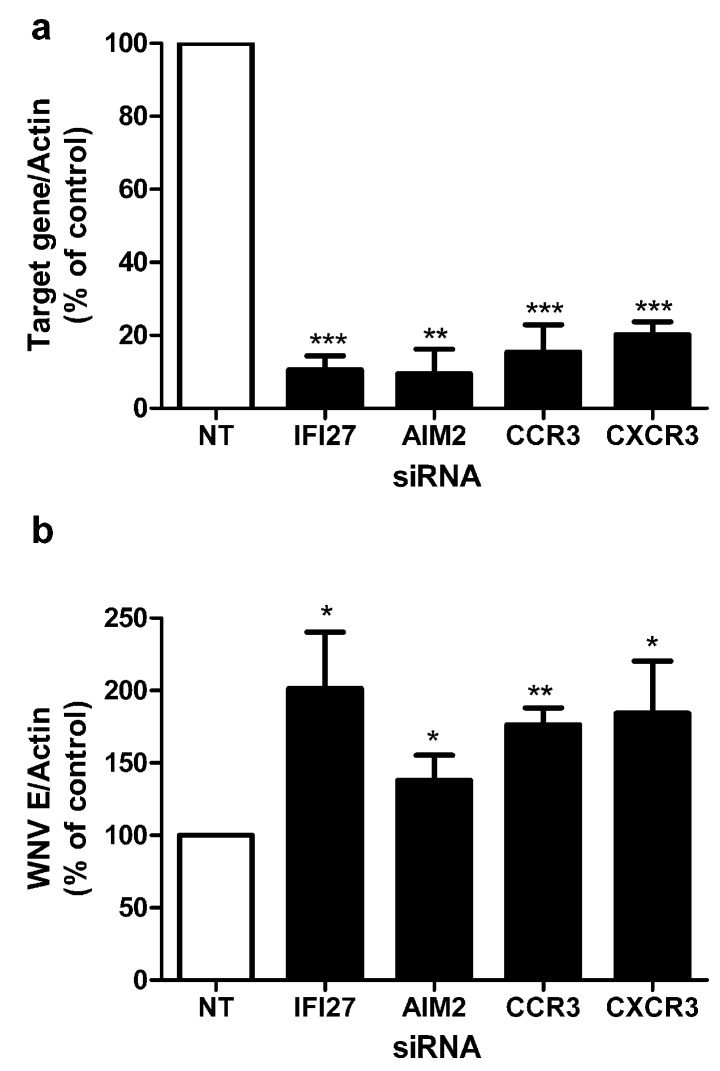
Effect of RNAi knockdown on WNV infection. Primary macrophages from healthy volunteers were transfected using the Nucleofection technology with siRNA targeting the genes shown and a non-targeting (nt) control siRNA. (**a**) The efficiency of RNAi knockdown after 48 h of infection with WNV was assessed via qPCR of target gene/actin compared with non-targeting control cells which was defined as 100% expression (n = 3). (**b**) WNV burdens in human macrophages after RNAi silencing. After 36 h of transfection, macrophages were infected with WNV (MOI = 1) for 48 h and viral load was quantified by qPCR. Data shown are the means ± SEM (*p < 0.05, ** *p* < 0.01, ***p < 0.001, T-test). Representative results from at least three independent experiments with successful RNAi silencing (>70%).

To dissect mechanisms of the anti-viral program in human macrophages, we further assessed a group of genes identified here by RNA-Seq. We have previously shown that knockdown of specific targets in primary macrophages can modulate efficiency of WNV infection and modify anti-viral responses [21]. To assess the role of differentially expressed genes identified by RNA-Seq analysis, siRNA duplexes corresponding to target genes were used to knockdown levels of the target protein in primary macrophages prior to infection with WNV. Target genes were selected which were common to all 10 donors and not previously known to be associated with macrophage response to WNV infection. The 732 common transcripts that were significantly differentially expressed by all 10 donors correspond to 564 unique genes (Supplementary Table S2), which we further characterized by functional annotation clustering analysis using DAVID. The molecular function of these genes is primarily characterized by their cytokine activity (GO:0005125, 11.4%), which is supported by the finding that genes associated with the “Cytokine-cytokine receptor interactions” (KEGG:hsa04060, 12.2%) were found to be over-represented in the sample list as compared to the background of the whole human genome. Enriched cluster contains additional KEGG pathway associated genes, most notably the pathways for “Toll-like receptor signaling” (KEGG:04620, 6.9%), “Cytosolic DNA‑sensing” (KEGG:hsa04623, 5.3%) and the pathway for “RIG-I-like receptor signaling” (KEGG:hsa04622, 5.8%). This observation supports the expectation that an *in vitro* WNV challenge of macrophages obtained from healthy donors triggers a strong anti-viral response. Since the enriched genes in these pathways are type I IFN and chemokines, two interferon-stimulated genes and two chemokine receptors were selected for RNAi knockdown. The targets selected were IFI27 (interferon alpha-inducible protein 27), AIM2 (absent in melanoma 2), CCR3 (receptor of chemokines CCL5 and CCL8), and CXCR3 (receptor of chemokine CXCL10), which is essential for control of WNV infection in the central nervous system [36]. When we quantified expression of these targets by Q‑PCR, each target gene, IFI27, AIM2, and CCL5, CCL8, and CXCL10—ligands for CCR3 and CXCR3—was upregulated in all 10 samples as confirmed by Q-PCR (average fold upregulation for IFI27, 133 fold; AIM2, 476 fold; CCL5, 249 fold; CCL8, 2724 fold; and CXCL10, 22772 fold; range *p* = 0.03–*p* = 0.008). Each of the genes was silenced efficiently as quantified by Q-PCR using gene specific primers; gene expression was determined after 48 h of infection with WNV and was decreased to 10%–20% of levels in non-targeting siRNA transfected control cells (Figure 3a). siRNA treated macrophages were infected with WNV and the viral load was determined by Q-PCR of the WNV envelope gene (E) at 48 h when detection of viral replication is maximal (Figure 3b). Of note, RNAi knockdown of each target resulted in increased viral load, suggesting increased efficiency of viral infection, and an important role for these proteins (or others they may affect) in resistance to infection with WNV. The identification of these genes through RNA-Seq and validation through knockdown studies has demonstrated their previously unrecognized role in control of WNV infection. 

## 3. Experimental Materials and Methods

### 3.1. Blood Donors and Preparation of Cells

Heparinized blood from healthy volunteers was obtained with written informed consent under an approved protocol of the Human Investigations Committee of Yale University School of Medicine. Donors had no acute illness, took no antibiotics or non-steroidal anti-inflammatory drugs [37]. Donors (n = 10) were aged 27.7 ± 3.9 years (range 22–36), 40% female and all were white. Peripheral blood mononuclear cells (PBMCs) were isolated using Ficoll-Hypaque (GE Healthcare, NJ, USA) and suspended in RPMI-1640 containing L-Glutamine supplemented with 20% (v/v) human serum (Lonza, MD, USA), 100 U/mL penicillin and 100 µg/mL streptomycin (Invitrogen, CA, USA). Cells were plated at 2 × 10^7^ cells/10 cm tissue culture dish. Non-adherent cells were washed away after 2 h, and adherent monocytes were incubated 6–8 days to mature into macrophages [21]. 

### 3.2. WNV Strains and Infections

Virulent WNV (CT-2741) was generously provided by Dr. John Anderson, Connecticut Agricultural Experiment Station, New Haven, CT, USA [38] and was passaged once in Vero cells (ATCC CCL-81) and viral PFU were quantified by plaque assays [21]. WNV studies were conducted in a Biosafety Level 3 facility, licensed by the State of Connecticut and Yale University. Cells were infected with WNV (multiplicity of infection, MOI = 1) for 24 h. Under these conditions, viral infection results in infection of 80%–90% of macrophages by 12 h as quantified by immunofluorescent staining using a monoclonal antibody directed against WNV E protein [21]. 

### 3.3. RNA Interference

Adherent monocyte-derived macrophages were dislodged using Trypsin/EDTA solution (0.5 mg/mL trypsin and 0.2 mg/mL EDTA in PBS) for 30 min at room temperature. Macrophages (2 × 10^6^) were transfected with 2 µM of small interfering RNAs (siRNAs) targeting IFI27, AIM2, CCR3 or CXCR3 (Dharmacon, CO, USA), using the nucleofection technology (Amaxa, MD, USA). Cells transfected with non-targeting siRNAs will be used as a control. Transfected cells were incubated for 36 h before infection studies. 

### 3.4. Quantitative Polymerase Chain Reaction (qPCR)

Total RNA was harvested from infected and uninfected macrophages using the RNeasy mini kit (Qiagen, MD, USA), and cDNA was synthesized using AffinityScript Multi Temperature cDNA Synthesis Kit (Stratagene, CA, USA) according to standard protocols. Amplification was performed in an iCycler (Bio-Rad, CA, USA) for 60 cycles with an annealing temperature at 60 °C and values were normalized to β-actin. Primers and probes for qPCRs were from Dharmacon or synthesized according to customized sequences as follows: WNV-E (forward: 5'- TTC TCG AAG GCG ACA GCT G-3', reverse: 5'- CCG CCT CCA TAT TCA TCA TC-3', probe: FAM ATG TCT AAG GAC AAG CCT ACC ATC TAMRA) and β-actin (forward: 5'-ATC CTG GCCTCG CTGTCCAC-3', reverse: 5'- GGG CCG GAC TCG TCA TAC-3', probe: FAM TCC AGC AGA TGT GGA TCA GCA AGC A TAMRA) [39]. 

### 3.5. Preparation of Libraries for Illumina Deep-Sequencing

mRNA was fragmented into 150–300 bp fragments by incubation in RNA Fragmentation Reagent (Ambion, TX, USA) at 70 °C for 5 minutes. The fragmented mRNA was purified away from the fragmentation buffer by Agencourt RNAClean beads (Beckman Coulter, MA, USA) following manufacturer’s instructions. The purified, fragmented mRNA was converted into double-stranded cDNA using the SuperScript Double-Stranded cDNA Synthesis Kit (Invitrogen, CA, USA) by priming with random hexamers. cDNA libraries were prepared for Illumina deep-sequencing as previously described [13]. 36 nucleotides of sequence were determined from one end of each cDNA fragment using high throughput DNA sequencing [40]. The RNA-Seq raw data files have been deposited in NCBI’s Gene Expression Omnibus (GEO) and are accessible through GEO Series accession number GSE40718 [41,42]. 

### 3.6. RNA-Seq Analysis

Hi-Seq sequence data from 10 donors (mock or WNV infected) was processed to obtain estimates expression level of genes and transcripts. Using the fastq files, all sequencing reads were mapped back to human genome (hg19) using default setting of TopHat (v1.1.4) [22]. Cufflinks (v0.9.3) [43], was used to assemble the mapped reads against the ENSEMBL (release 57) gene structure annotation, and estimate expression levels for each transcript [23], and was not allowed to modify the gene annotations. To estimate isoform-level abundances, Cufflinks uses a probabilistic model of paired-end sequencing to derive a likelihood for the abundance of transcripts to calculate ambiguous isoforms. To analyze the gene expression of human macrophages we first converted the estimated expression levels in FPKM unit (# reads × 10^9^/transcript length/library) to pseudocounts (# of reads originated from each transcript isoform). The effective transcript length reported by Cufflinks is used for the conversion. The pseudocounts were further normalized across samples using the trimmed mean of M‑values (TMM) method [44]. Differentially expressed transcripts were identified using edgeR and Bayesian DE paired analysis. Transcripts were excluded if <10 in paired conditions or expressed in ≤5 subjects. The resulting transcript set was 36,409 transcripts. To be considered differentially expressed, a transcript may be present in either mock or WNV-infected samples. 

For the functional annotation clustering, the 1,514 differentially expressed transcripts were submitted for analysis using the DAVID web server [29]. The “Medium” default settings were used for classification stringency. The functional annotation clustering is based on the occurrence of annotation terms e.g., Gene Ontology (GO) [45] in the list of sample genes as compared to the occurrence of these terms in the “background” or “population”. The known annotations for the entire human genome in the categories “GO—Biological Process”, “GO—Cellular Component”, “GO—Molecular Function”, “SP_PIR_KEYWORDS”, “PIR_SUPERFAMILY”, “INTERPRO”, “SMART” and “KEGG pathways” were selected as background [46].

## 4. Conclusions

We have employed RNASeq to provide a comprehensive identification of the critical elements in the host innate immune response to infection with WNV. In contrast to microarray experiments, which use pre-defined oligonucleotide probes for measuring differential expression of genes, RNA-Seq as used in this experiment does not introduce a selection bias in terms of the background or gene population. The abundant gene transcripts identified through RNASeq require high level bioinformatics analysis and our analysis achieves higher statistical power through combination of samples. To evaluate both common gene pathways as well as novel cellular responses will be essential, as we examine differences between susceptible and resistant individuals in the population. 

In our analysis we have shown both predicted and novel gene changes in response to infection with WNV. Several targets identified here as differentially expressed on infection with WNV are common host factors—such as the IFITM proteins—that similarly mediate cellular resistance to other viruses including influenza A virus and dengue virus [47]. The interferon (IFN) response is an essential host defense program that limits viral infection and was shown here with the upregulation of many interferon-inducible genes including MX1, OAS1. These targets are of particular interest as recent genomic studies have identified polymorphisms in OAS1 and MX1 that are associated with increased risk of symptomatic WNV infection [9,48,49]. Similarly, chemokines and chemokine receptors have been shown to be important in control of WNV infection [11,50]. In humans, a 32-bp deletion in the coding region of the CC chemokine receptor 5 (CCR5Δ32) was reported to be associated with both increased susceptibility to WNV infection and death [51,52], and in mice, CXCR3 and its ligand CXCL10 are required for T cell-mediated clearance of WNV primarily within the cerebellum during WNV encephalitis [36]. In this study, we demonstrate upregulation of chemokines CCL5 and CCL8 after WNV infection, and increased viral load following RNAi knockdown of their receptor CCR3, confirming generally accepted roles in control of WNV infection. We also detected an increase in expression of TLR3 at 24 h, even though TLR3 is dramatically down regulated in macrophages of young donors at 3 h, suggesting a dynamic regulation of TLR3 in response to WNV [21].

In addition, we have identified a large number of genes not previously associated with WNV responses. One such gene identified here, IFI27, is a mitochondrial protein that sensitizes cells to apoptotic stimuli via mitochondrial membrane destabilization [53]. Its identification suggests a novel mechanism of pathogenesis of WNV infection involving a role for pro-apoptotic signaling and a potential avenue for intervention. Our analysis also identified the involvement of several pathways for which a role in the host response to WNV had not been fully investigated, including the activation of inflammasome signaling. We observed downregulation of NLRP1 and NLRC4 and upregulation of NLRP3 and AIM2 during WNV infection. AIM2 is a cytosolic DNA receptor that forms an inflammasome with ASC to trigger caspase 1 activation [54] for which a role in RNA virus infection was unknown. A role for inflammasomes in WNV infection has recently been described in a murine model [55]. The increased viral load shown after RNAi knockdown of AIM2 in this study suggests an important role for AIM2 in control of WNV infection.

**Figure 4:**
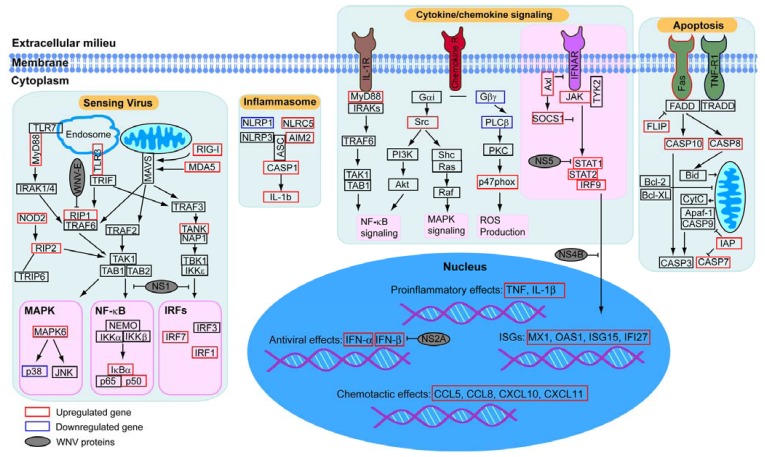
Key signaling pathways modulated by WNV infection. Genes within selected pathways identified in the RNAseq analysis were placed on the map using annotation information from Gene Ontology, KEGG Pathway, Biocarta Pathway, PANTHER and Reactome. Transcriptional regulation in response to WNV in human macrophages is indicated by boxes (black, unchanged; red increase in gene expression; blue for decreased expression). Interactions between WNV proteins and host components are based on previous reports and are shown in grey.

To build a model of the key signaling pathways modulated by WNV infection, we focused on functional annotation of pathways with many differentially regulated genes identified by our analysis, such as viral sensing pathways including TLR and RLR, which recognize specific pathogen associated molecular patterns (PAMPs) and initiate signal transduction pathways (Figure 4). Our model includes upregulation of key WNV recognition receptors such as TLR3, TLR7, MDA5 and RIG-I, which are known to control WNV infection in mouse models [56]. Similarly, we demonstrate upregulation of many cytokines, chemokines, including type 1 interferon and proinflammatory cytokines, and their receptors and signaling pathways which well-characterized roles in anti-WNV responses [57,58]. Our analysis also highlights additional key cellular components including the activation of the autophagy and apoptosis pathways. The autophagy machinery can be used to deliver viral genetic material to endosomal TLRs for efficient induction of type I interferon and has been linked to diverse aspects of innate and adaptive immunity [59]. Many viruses encode proteins that can inhibit apoptosis [60] and the triggering of apoptosis has been noted in WNV-infected neuronal tissue [61], but details of the roles for autophagy and apoptosis in WNV pathogenesis in innate immunity are not fully understood. Finally, when we incorporated WNV proteins that directly associate with these pathways into the model, we observed that the virus can target diverse signaling pathways through both physical and regulatory interactions. This observation emphasizes the regulation and counter-regulation between host and virus. This method should prove valuable for translational studies of resistant and susceptible populations, or responders and non-responders—and for targeting therapeutics—in multiple biological settings.

## Supplementary Files

Supplementary File 1 Supplementary (ZIP, 454 KB)
