# Supplementary material for: Identification of Genes Critical for Resistance to Infection by West Nile Virus Using RNA-Seq Analysis
Source: Viruses. 2013 Jul 8;5(7):1664–81. doi: 10.3390/v5071664 (PMC3738954; doi:10.3390/v5071664)
Supplement: Supplementary File 1 — Supplementary (ZIP, 454 KB) [file viruses-05-01664-s001.zip › Qian_Table S1 RNA-Seq sample mapping summary.pdf]

Table S1 RNA-Seq Subject mapping summary

Sample	Total_filtered_reads	Tophat_mapped_reads	Unique_hits	#(2 hits)	#(>2 hits)	Splice_alignments	Map_percentage
Subject1 mock	29922477	26773755	21142047	2306561	3325147	2806037	89.48%
Subject2 mock	29996610	25700116	20509594	2123347	3067175	2680850	85.68%
Subject3 mock	29676563	26254698	20737318	2225931	3291449	2740810	88.47%
Subject4 mock	25817531	20729674	16083833	1883729	2762112	2120403	80.29%
Subject5 mock	26965296	24405800	19486598	1920550	2998652	2535653	90.51%
Subject6 mock	26900450	24057866	19045097	1980317	3032452	2470119	89.43%
Subject7 mock	27023417	22863435	17918320	1921850	3023265	2554503	84.61%
Subject8 mock	30954809	27646983	22586755	2005423	3054805	2827679	89.31%
Subject9 mock	30034811	27651798	23394336	1742483	2514979	2675800	92.07%
Subject10 mock	29084544	25752069	21449731	1747538	2554800	2684162	88.54%
Subject1 infected	31423035	29234002	24539712	1870818	2823472	3005014	93.03%
Subject2 infected	26606088	22577040	18339803	1656295	2580942	2452830	84.86%
Subject3 infected	28869391	27125200	22238152	1966117	2920931	2792203	93.96%
Subject4 infected	30178658	26639669	21573294	1989027	3077348	2825635	88.27%
Subject5 infected	30064035	27494737	22516790	1974384	3003563	2765992	91.45%
Subject6 infected	29003531	26632199	21878939	1868949	2884311	2813084	91.82%
Subject7 infected	29775059	24127800	19461319	1824800	2841681	2648383	81.03%
Subject8 infected	26245391	22220813	18020732	1608350	2591731	2418831	84.67%
Subject9 infected	22694314	20705016	17778532	1150971	1775513	2003159	91.23%
Subject10 infected	25723307	22441808	19070864	1345836	2025108	2257434	87.24%
