# Supplementary material for: Identification of Genes Critical for Resistance to Infection by West Nile Virus Using RNA-Seq Analysis
Source: Viruses. 2013 Jul 8;5(7):1664–81. doi: 10.3390/v5071664 (PMC3738954; doi:10.3390/v5071664)
Supplement: Supplementary File 1 — Supplementary (ZIP, 454 KB) [file viruses-05-01664-s001.zip › Qian_Table S2. Differentially expressed transcripts common to all donors.pdf]

Table S2. Differentially expressed transcripts common to all donors

Ensembl.Transcript.ID	Transcript.Name	Ensembl.Gene.ID	Gene.Name	Average Fold change log2 fold change	SD
ENST00000371818	IFIT3-001	ENSG00000119917	IFIT3	16.88	0.45
ENST00000371804	IFIT1-001	ENSG00000185745	IFIT1	16.53	0.42
ENST00000371826	IFIT2-001	ENSG00000119922	IFIT2	16.35	0.89
ENST00000371811	IFIT3-002	ENSG00000119917	IFIT3	14.24	0.70
ENST00000335760	APOBEC3B-005	ENSG00000179750	APOBEC3B	12.36	0.33
ENST00000483004	CFB-013	ENSG00000243649	CFB	10.75	0.66
ENST00000432102	NCF1B-002	ENSG00000182487	NCF1B	11.18	0.76
ENST00000380205	IFNA8-001	ENSG00000120242	IFNA8	10.68	1.00
ENST00000251630	PDGFRL-001	ENSG00000104213	PDGFRL	9.99	0.62
ENST00000389070	CCL4-201	ENSG00000129277	CCL4	11.73	1.33
ENST00000249116	APOBEC3A-201	ENSG00000128383	APOBEC3A	10.27	1.02
ENST00000379224	ISG20-201	ENSG00000172183	ISG20	11.20	1.17
ENST00000306602	CXCL10-201	ENSG00000169245	CXCL10	12.88	3.01
ENST00000259555	IFNA13-201	ENSG00000233816	IFNA13	10.59	1.13
ENST00000358756	CCL4L1-201	ENSG00000205020	CCL4L1	9.62	1.00
ENST00000380206	IFNA2-001	ENSG00000188379	IFNA2	11.49	1.24
ENST00000253513	IDO1-201	ENSG00000131203	IDO1	12.15	2.27
ENST00000276927	IFNA1-001	ENSG00000197919	IFNA1	10.54	1.58
ENST00000257570	OASL-001	ENSG00000135114	OASL	9.80	0.90
ENST00000382040	RSAD2-001	ENSG00000134321	RSAD2	10.97	1.93
ENST00000481829	AIM2-003	ENSG00000163568	AIM2	8.73	0.28
ENST00000368130	AIM2-001	ENSG00000163568	AIM2	9.40	1.02
ENST00000464998	NEXN-007	ENSG00000162614	NEXN	10.10	0.86
ENST00000433458	NCF1-014	ENSG00000158517	NCF1	10.52	0.98
ENST00000306621	CXCL11-001	ENSG00000169248	CXCL11	12.35	2.38
ENST00000472581	CFB-004	ENSG00000243649	CFB	8.88	0.74
ENST00000377462	IRG1-001	ENSG00000102794	IRG1	11.15	1.58
ENST00000243347	TNFAIP6-001	ENSG00000123610	TNFAIP6	10.57	1.39
ENST00000380232	IFNB1-002	ENSG00000171855	IFNB1	11.38	1.89
ENST00000389069	CCL4L2-202	ENSG00000197262	CCL4L2	8.97	1.05
ENST00000225840	CCL8-201	ENSG00000108700	CCL8	12.12	2.27
ENST00000402522	TBC1D1-002	ENSG00000065882	TBC1D1	8.43	0.46
ENST00000380229	IFNW1-001	ENSG00000177047	IFNW1	9.23	1.27
ENST00000370751	IFI44L-001	ENSG00000137959	IFI44L	10.61	1.75
ENST00000491194	ADORA2A-006	ENSG00000128271	ADORA2A	8.84	1.06
ENST00000306072	ISG20-001	ENSG00000172183	ISG20	10.18	2.14
ENST00000241261	TNFSF10-001	ENSG00000121858	TNFSF10	9.59	2.53
ENST00000298902	IFI27-201	ENSG00000165949	IFI27	8.61	1.04
ENST00000328221	IFITM1-201	ENSG00000185885	IFITM1	8.51	1.29
ENST00000461547	ZBP1-004	ENSG00000124256	ZBP1	9.27	1.27
ENST00000420541	TNFSF10-002	ENSG00000121858	TNFSF10	8.85	1.86
ENST00000397372	HSH2D-202	ENSG00000196684	HSH2D	9.43	1.96
ENST00000366113	CCL5-002	ENSG00000161570	CCL5	10.30	1.71
ENST00000380222	IFNA14-001	ENSG00000228083	IFNA14	10.28	1.61
ENST00000449498	IFNA13-001	ENSG00000233816	IFNA13	9.43	1.46
ENST00000264350	HERC5-001	ENSG00000138646	HERC5	7.73	0.62
ENST00000453793	ZBP1-005	ENSG00000124256	ZBP1	7.60	0.42
ENST00000502093	AC017076-201	ENSG00000247107	AC017076.1	8.07	1.01
ENST00000441683	USP18-201	ENSG00000184979	USP18	9.90	1.78
ENST00000395822	ZBP1-003	ENSG00000124256	ZBP1	8.72	1.12
ENST00000340181	ETV7-001	ENSG00000010030	ETV7	9.93	1.71
ENST00000297439	DEFB1-001	ENSG00000164825	DEFB1	8.49	0.87
ENST00000246657	CCR7-001	ENSG00000126353	CCR7	8.95	1.17
ENST00000431042	SLC2A14-203	ENSG00000173262	SLC2A14	7.47	0.57

ENST00000478119	GCH1-004	ENSG00000131979	GCH1	10.51	1.60
ENST00000398598	MX1-002	ENSG00000157601	MX1	10.76	3.10
ENST00000338578	AC006995-201	ENSG00000241849	AC006995.3	8.62	1.11
ENST00000322982	AC005013-001	ENSG00000176734	AC005013.2	7.73	0.69
ENST00000332579	IFNE-201	ENSG00000184995	IFNE	8.18	1.19
ENST00000226279	CD38-001	ENSG00000004468	CD38	10.28	1.91
ENST00000265404	STAP1-001	ENSG00000035720	STAP1	8.04	1.22
ENST00000411427	AP001610-001	ENSG00000228318	AP001610.1	7.77	1.00
ENST00000379389	ISG15-001	ENSG00000187608	ISG15	7.83	1.00
ENST00000368605	FAM26F-001	ENSG00000188820	FAM26F	8.29	1.83
ENST00000380225	IFNA21-001	ENSG00000137080	IFNA21	7.57	0.97
ENST00000333625	IL29-201	ENSG00000182393	IL29	10.12	1.55
ENST00000355754	GBP4-001	ENSG00000162654	GBP4	8.20	0.98
ENST00000397689	AC009729-201	ENSG00000214163	AC009729.1	7.47	0.52
ENST00000451794	NEURL3-202	ENSG00000163121	NEURL3	8.99	1.43
ENST00000297905	NCF1C-001	ENSG00000165178	NCF1C	7.70	0.93
ENST00000419310	AC106760-201	ENSG00000233100	AC106760.1	7.17	1.02
ENST00000313640	EPSTI1-003	ENSG00000133106	EPSTI1	7.26	0.51
ENST00000417291	GBP5-202	ENSG00000154451	GBP5	7.02	0.82
ENST00000358127	PAX5-001	ENSG00000196092	PAX5	8.95	1.49
ENST00000443956	NCF1-003	ENSG00000158517	NCF1	9.80	1.69
ENST00000394662	RP4-644F6-001	ENSG00000225492	RP4-644F6.2	6.55	0.35
ENST00000371852	CH25H-001	ENSG00000138135	CH25H	7.92	1.05
ENST00000422952	GBP1-201	ENSG00000117228	GBP1	8.17	3.28
ENST00000500521	AL357054-201	ENSG00000247005	AL357054.2	8.35	1.38
ENST00000293272	CCL5-001	ENSG00000161570	CCL5	10.47	2.59
ENST00000412609	AC093063-001	ENSG00000226025	AC093063.1	7.98	1.25
ENST00000339598	OTOF-003	ENSG00000115155	OTOF	7.34	1.71
ENST00000392583	OAS2-202	ENSG00000111335	OAS2	7.05	0.97
ENST00000495160	HESX1-001	ENSG00000163666	HESX1	7.90	1.46
ENST00000470202	USP41-002	ENSG00000161133	USP41	7.42	1.27
ENST00000292729	USP41-001	ENSG00000161133	USP41	7.54	0.95
ENST00000296657	ANKRD33B-201	ENSG00000164236	ANKRD33B	6.66	0.74
ENST00000317669	SLC38A5-201	ENSG00000017483	SLC38A5	8.98	2.30
ENST00000259698	FAM65B-001	ENSG00000111913	FAM65B	7.67	1.07
ENST00000235739	SLAMF1-201	ENSG00000117090	SLAMF1	7.03	0.85
ENST00000229635	FAM65B-201	ENSG00000111913	FAM65B	7.40	1.06
ENST00000295934	HESX1-201	ENSG00000163666	HESX1	7.51	1.09
ENST00000357374	IFNA10-001	ENSG00000186803	IFNA10	7.13	1.08
ENST00000264888	CXCL9-201	ENSG00000138755	CXCL9	9.40	2.01
ENST00000342754	NEXN-005	ENSG00000162614	NEXN	8.75	2.21
ENST00000397250	IL15RA-203	ENSG00000134470	IL15RA	7.26	1.61
ENST00000259727	GMPR-001	ENSG00000137198	GMPR	6.39	0.56
ENST00000435988	NCF1B-001	ENSG00000182487	NCF1B	7.43	1.05
ENST00000478738	CMPK2-004	ENSG00000134326	CMPK2	8.66	1.89
ENST00000480037	ZBP1-002	ENSG00000124256	ZBP1	8.04	1.27
ENST00000057513	TNIP3-001	ENSG00000050730	TNIP3	7.51	1.38
ENST00000394465	CCL4L2-001	ENSG00000197262	CCL4L2	8.07	1.45
ENST00000239347	IFNA7-001	ENSG00000214042	IFNA7	7.06	0.98
ENST00000203629	LAG3-201	ENSG00000089692	LAG3	7.95	2.19
ENST00000215794	USP18-001	ENSG00000184979	USP18	6.63	0.78
ENST00000330714	MX2-001	ENSG00000183486	MX2	6.55	0.66
ENST00000417932	RP11-10J5-001	ENSG00000226004	RP11-10J5.1	7.64	1.05
ENST00000304952	HES4-001	ENSG00000188290	HES4	7.46	2.04
ENST00000289473	NCF1-001	ENSG00000158517	NCF1	7.09	0.96
ENST00000370091	RP4-697K14-202	ENSG00000130589	RP4-697K14.2	8.94	3.02
ENST00000342315	OAS2-201	ENSG00000111335	OAS2	5.93	0.31

ENST00000447291	IL15RA-008	ENSG00000134470	IL15RA	8.19	1.62
ENST00000500182	AC021937-201	ENSG00000245625	AC021937.1	8.28	1.43
ENST00000396367	BCL2L14-202	ENSG00000121380	BCL2L14	8.31	1.35
ENST00000398421	NCF1-002	ENSG00000158517	NCF1	8.39	2.25
ENST00000429713	SYNPO2-203	ENSG00000172403	SYNPO2	7.71	1.25
ENST00000371930	ANKRD22-001	ENSG00000152766	ANKRD22	7.21	1.56
ENST00000495658	C15orf21-002	ENSG00000179362	C15orf21	7.77	1.13
ENST00000310865	NEURL3-002	ENSG00000163121	NEURL3	8.88	1.43
ENST00000359595	HAPLN3-001	ENSG00000140511	HAPLN3	7.75	1.78
ENST00000256722	CMPK2-002	ENSG00000134326	CMPK2	9.30	2.35
ENST00000380216	IFNA16-001	ENSG00000147885	IFNA16	6.93	1.18
ENST00000263265	PLEKHA4-201	ENSG00000105559	PLEKHA4	8.73	2.74
ENST00000263642	IFIH1-001	ENSG00000115267	IFIH1	6.28	0.57
ENST00000370459	GBP5-001	ENSG00000154451	GBP5	7.21	1.53
ENST00000479540	RP4-697K14-005	ENSG00000130589	RP4-697K14.2	5.87	0.62
ENST00000332029	SOCS1-001	ENSG00000185338	SOCS1	6.14	0.68
ENST00000413767	IFNA17-001	ENSG00000234829	IFNA17	6.80	1.09
ENST00000495089	NFKBIZ-009	ENSG00000144802	NFKBIZ	6.53	1.00
ENST00000437196	EXT1-004	ENSG00000182197	EXT1	7.80	2.03
ENST00000398657	NKAIN1-202	ENSG00000084628	NKAIN1	7.86	1.29
ENST00000370747	IFI44-001	ENSG00000137965	IFI44	7.25	1.99
ENST00000479395	EIF2C3-006	ENSG00000126070	EIF2C3	6.32	0.96
ENST00000447787	AC009123-201	ENSG00000234532	AC009123.1	7.84	1.55
ENST00000458710	IFNA22P-201	ENSG00000224416	IFNA22P	7.66	1.43
ENST00000378553	LRRC50-001	ENSG00000154099	LRRC50	7.88	2.04
ENST00000395206	C20orf195-201	ENSG00000125531	C20orf195	7.23	1.61
ENST00000360954	HS3ST3B1-001	ENSG00000125430	HS3ST3B1	7.10	1.68
ENST00000396225	STAP1-002	ENSG00000035720	STAP1	6.82	1.23
ENST00000254299	GCH1-001	ENSG00000131979	GCH1	7.49	2.25
ENST00000200307	CCL7-201	ENSG00000108688	CCL7	7.70	2.25
ENST00000380212	IFNA5-001	ENSG00000147873	IFNA5	7.98	1.58
ENST00000431790	XAF1-204	ENSG00000132530	XAF1	5.69	0.64
ENST00000392619	CD300E-001	ENSG00000186407	CD300E	7.05	1.40
ENST00000378204	EXT1-001	ENSG00000182197	EXT1	6.20	0.91
ENST00000482953	MX2-002	ENSG00000183486	MX2	5.90	0.53
ENST00000311925	CCL19-001	ENSG00000172724	CCL19	7.66	1.45
ENST00000450152	RP11-464C19-002	ENSG00000226005	RP11-464C19.[illegible]	7.12	1.36
ENST00000441170	AC092661-002	ENSG00000213492	AC092661.2	5.82	0.60
ENST00000242210	NT5C3-001	ENSG00000122643	NT5C3	6.49	2.57
ENST00000296795	TLR3-201	ENSG00000164342	TLR3	5.99	1.06
ENST00000155926	TRIB2-001	ENSG00000071575	TRIB2	6.19	1.21
ENST00000370473	GBP1-001	ENSG00000117228	GBP1	6.05	0.82
ENST00000367468	PTGS2-001	ENSG00000073756	PTGS2	6.61	1.63
ENST00000225831	CCL2-001	ENSG00000108691	CCL2	6.39	0.90
ENST00000449065	AC023590-001	ENSG00000225885	AC023590.1	6.90	1.31
ENST00000491738	CMPK2-007	ENSG00000134326	CMPK2	8.36	2.02
ENST00000483300	PLSCR1-018	ENSG00000188313	PLSCR1	7.10	2.55
ENST00000397289	APOL3-006	ENSG00000128284	APOL3	10.03	3.21
ENST00000421715	IFNA4-001	ENSG00000236637	IFNA4	6.18	0.99
ENST00000436469	AC022816-001	ENSG00000230647	AC022816.1	7.10	1.61
ENST00000252889	RP4-697K14-201	ENSG00000130589	RP4-697K14.2	6.67	2.36
ENST00000458235	JAK3-204	ENSG00000105639	JAK3	5.63	0.65
ENST00000301887	BATF2-201	ENSG00000168062	BATF2	5.82	1.92
ENST00000313624	EPSTI1-001	ENSG00000133106	EPSTI1	6.50	0.89
ENST00000307407	IL8-001	ENSG00000169429	IL8	7.50	1.78
ENST00000379958	SAMD9-001	ENSG00000205413	SAMD9	5.28	0.61
ENST00000436551	AC104654-002	ENSG00000234362	AC104654.2	5.54	0.66

ENST00000296604	RANBP3L-001	ENSG00000164188	RANBP3L	7.24	1.57
ENST00000399808	IFITM3-202	ENSG00000142089	IFITM3	5.63	0.87
ENST00000435842	BATF2-202	ENSG00000168062	BATF2	7.26	2.47
ENST00000452428	IFITM3-203	ENSG00000142089	IFITM3	5.64	0.68
ENST00000318238	SAMD9L-001	ENSG00000177409	SAMD9L	4.84	0.33
ENST00000381577	CD274-001	ENSG00000120217	CD274	5.02	0.57
ENST00000228928	OAS3-201	ENSG00000111331	OAS3	5.31	0.51
ENST00000284027	MCOLN2-001	ENSG00000153898	MCOLN2	6.91	2.39
ENST00000463146	AC025580-201	ENSG00000240564	AC025580.6	5.44	0.61
ENST00000500660	AL024507-201	ENSG00000246971	AL024507.3	7.55	1.45
ENST00000261867	SLC30A4-001	ENSG00000104154	SLC30A4	5.96	0.78
ENST00000473070	FAM65B-003	ENSG00000111913	FAM65B	6.81	1.14
ENST00000474368	MX2-004	ENSG00000183486	MX2	5.54	0.74
ENST00000448696	IFNE-001	ENSG00000184995	IFNE	5.73	0.91
ENST00000382210	IL32-203	ENSG00000008517	IL32	5.87	1.01
ENST00000426204	RP11-202G18-001	ENSG00000227531	RP11-202G18.	6.05	1.05
ENST00000470735	NEXN-006	ENSG00000162614	NEXN	7.29	1.38
ENST00000462359	SLC38A5-006	ENSG00000017483	SLC38A5	5.55	1.07
ENST00000281282	CGNL1-001	ENSG00000128849	CGNL1	6.50	1.05
ENST00000357461	TMEM229B-201	ENSG00000198133	TMEM229B	5.44	0.51
ENST00000404898	CACNA1I-002	ENSG00000100346	CACNA1I	6.27	1.18
ENST00000475369	C1orf224-002	ENSG00000237276	C1orf224	5.73	0.83
ENST00000244869	EREG-001	ENSG00000124882	EREG	6.35	1.45
ENST00000300119	MYO1A-001	ENSG00000166866	MYO1A	6.02	1.01
ENST00000442657	HOXB9-201	ENSG00000170689	HOXB9	5.39	0.74
ENST00000259008	BRIP1-201	ENSG00000136492	BRIP1	6.37	1.72
ENST00000482622	AKT1S1-007	ENSG00000204673	AKT1S1	8.26	2.46
ENST00000441631	XAF1-206	ENSG00000132530	XAF1	8.72	3.35
ENST00000473921	HESX1-002	ENSG00000163666	HESX1	6.46	1.11
ENST00000265598	LAMP3-001	ENSG00000078081	LAMP3	6.93	1.77
ENST00000329654	PDE4B-001	ENSG00000184588	PDE4B	7.06	2.01
ENST00000466596	HS3ST3B1-002	ENSG00000125430	HS3ST3B1	6.04	1.18
ENST00000409454	C15orf21-001	ENSG00000179362	C15orf21	5.25	0.65
ENST00000394495	CCL4-202	ENSG00000129277	CCL4	8.35	2.42
ENST00000499665	AL137186-201	ENSG00000244816	AL137186.1	6.00	1.12
ENST00000380210	IFNA6-001	ENSG00000120235	IFNA6	5.22	0.91
ENST00000263247	APOBEC3G-201	ENSG00000239713	APOBEC3G	4.62	0.39
ENST00000339270	CCL4L2-002	ENSG00000197262	CCL4L2	8.21	2.04
ENST00000391826	IL4I1-203	ENSG00000104951	IL4I1	4.79	0.43
ENST00000356897	IL27-001	ENSG00000197272	IL27	5.87	1.03
ENST00000375887	TNFSF13B-001	ENSG00000102524	TNFSF13B	4.97	0.52
ENST00000442064	RP4-725G10-001	ENSG00000230191	RP4-725G10.4	5.86	0.93
ENST00000371045	PDE4B-007	ENSG00000184588	PDE4B	6.48	1.88
ENST00000290575	C1R-201	ENSG00000159403	C1R	6.27	0.92
ENST00000474669	PARP14-003	ENSG00000173193	PARP14	9.23	4.10
ENST00000481869	HES4-003	ENSG00000188290	HES4	7.16	2.41
ENST00000394809	MYLK3-001	ENSG00000140795	MYLK3	5.56	1.26
ENST00000327442	KPNA7-001	ENSG00000185467	KPNA7	6.07	1.32
ENST00000413928	AC015849-001	ENSG00000237805	AC015849.1	6.32	1.40
ENST00000228434	CD69-201	ENSG00000110848	CD69	5.57	0.82
ENST00000478182	CD80-002	ENSG00000121594	CD80	4.73	0.51
ENST00000471652	ZC3HAV1-003	ENSG00000105939	ZC3HAV1	9.90	2.72
ENST00000423083	NCF1B-003	ENSG00000182487	NCF1B	5.66	1.15
ENST00000357585	SSTR2-202	ENSG00000180616	SSTR2	5.62	1.21
ENST00000263341	IL1B-001	ENSG00000125538	IL1B	7.09	2.18
ENST00000453713	AC023157-201	ENSG00000223722	AC023157.1	5.53	0.98
ENST00000393255	AC025280-201	ENSG00000213275	AC025280.1	5.33	0.83

ENST00000222573	ITGB8-001	ENSG00000105855	ITGB8	5.47	0.94
ENST00000259030	RTP4-001	ENSG00000136514	RTP4	4.28	0.29
ENST00000253680	HSH2D-201	ENSG00000196684	HSH2D	7.60	2.23
ENST00000370292	CSAG2-001	ENSG00000184324	CSAG2	4.93	0.70
ENST00000263816	LRP2-001	ENSG00000081479	LRP2	5.79	1.37
ENST00000315994	SH3PXD2A-002	ENSG00000107957	SH3PXD2A	9.05	1.89
ENST00000295136	AC092653-201	ENSG00000244120	AC092653.1	5.65	0.85
ENST00000425368	CFB-001	ENSG00000243649	CFB	10.06	2.30
ENST00000435171	IL15RA-009	ENSG00000134470	IL15RA	5.74	1.80
ENST00000396331	LILRB1-002	ENSG00000104972	LILRB1	4.78	0.65
ENST00000460845	ZC3HAV1-004	ENSG00000105939	ZC3HAV1	4.92	2.14
ENST00000295908	PPM1K-001	ENSG00000163644	PPM1K	4.58	0.34
ENST00000370608	MCOLN2-201	ENSG00000153898	MCOLN2	6.37	2.37
ENST00000448787	PLSCR1-003	ENSG00000188313	PLSCR1	8.07	3.39
ENST00000433668	SERPING1-203	ENSG00000149131	SERPING1	4.71	0.62
ENST00000216099	APOBEC3D-001	ENSG00000243811	APOBEC3D	7.74	2.90
ENST00000379775	PFKFB3-002	ENSG00000170525	PFKFB3	4.70	0.81
ENST00000449264	TNF-001	ENSG00000232810	TNF	5.91	0.87
ENST00000456021	ASPHD2-202	ENSG00000128203	ASPHD2	6.46	1.21
ENST00000401395	KIAA1671-202	ENSG00000197077	KIAA1671	6.24	1.93
ENST00000316660	PMAIP1-001	ENSG00000141682	PMAIP1	4.87	1.02
ENST00000415810	CSAG3-001	ENSG00000197463	CSAG3	4.57	0.56
ENST00000427522	RP4-697K14-001	ENSG00000130589	RP4-697K14.2	6.98	3.26
ENST00000479435	TNFSF13B-002	ENSG00000102524	TNFSF13B	7.99	2.70
ENST00000477392	RP11-511P7-001	ENSG00000242258	RP11-511P7.1	5.96	1.61
ENST00000394472	SAMD9L-201	ENSG00000177409	SAMD9L	5.29	0.99
ENST00000391835	AKT1S1-002	ENSG00000204673	AKT1S1	4.76	0.73
ENST00000328880	GPR109A-201	ENSG00000182782	GPR109A	5.66	1.38
ENST00000269202	MEP1B-201	ENSG00000141434	MEP1B	5.01	1.03
ENST00000319921	AC124319-201	ENSG00000180843	AC124319.2	3.97	0.24
ENST00000407997	APOBEC3G-001	ENSG00000239713	APOBEC3G	6.25	2.82
ENST00000348655	IRF7-003	ENSG00000185507	IRF7	7.87	3.14
ENST00000353172	KCTD14-001	ENSG00000151364	KCTD14	5.20	1.31
ENST00000400837	CRLF2-204	ENSG00000205755	CRLF2	5.26	1.05
ENST00000418094	IDO2-003	ENSG00000188676	IDO2	5.74	0.91
ENST00000378023	FAM65B-002	ENSG00000111913	FAM65B	6.57	1.29
ENST00000443559	IFITM2-203	ENSG00000185201	IFITM2	4.86	0.90
ENST00000489444	GBP3-002	ENSG00000117226	GBP3	6.31	2.91
ENST00000392048	SP110-004	ENSG00000135899	SP110	3.88	0.24
ENST00000380698	SERPINB9-001	ENSG00000170542	SERPINB9	4.35	0.60
ENST00000495622	ITIH3-010	ENSG00000162267	ITIH3	4.25	0.51
ENST00000329464	TRIM69-201	ENSG00000185880	TRIM69	5.94	1.27
ENST00000354258	TAP1-001	ENSG00000168394	TAP1	4.35	0.43
ENST00000469048	IRF7-004	ENSG00000185507	IRF7	5.18	0.98
ENST00000478442	RABGAP1L-010	ENSG00000152061	RABGAP1L	5.96	2.68
ENST00000269518	PMAIP1-002	ENSG00000141682	PMAIP1	5.68	1.79
ENST00000441828	RUFY4-202	ENSG00000188282	RUFY4	5.90	1.69
ENST00000468219	CLEC19A-204	ENSG00000188477	CLEC19A	4.10	0.61
ENST00000361099	STAT1-001	ENSG00000115415	STAT1	4.85	0.85
ENST00000461384	GBP3-007	ENSG00000117226	GBP3	5.38	1.85
ENST00000466205	CD40-006	ENSG00000101017	CD40	5.85	3.04
ENST00000470496	PLSCR1-020	ENSG00000188313	PLSCR1	6.46	2.63
ENST00000361112	SLFN12L-201	ENSG00000205045	SLFN12L	5.10	1.16
ENST00000480395	TRIM22-002	ENSG00000132274	TRIM22	4.57	0.61
ENST00000371795	IFIT5-001	ENSG00000152778	IFIT5	3.98	0.32
ENST00000378354	CCL3L1-201	ENSG00000205021	CCL3L1	6.90	2.34
ENST00000251642	DHX58-001	ENSG00000108771	DHX58	3.89	0.21

ENST00000493432	PLSCR1-005	ENSG00000188313	PLSCR1	5.04	0.81
ENST00000342887	C12orf63-201	ENSG00000188596	C12orf63	5.13	0.96
ENST00000343295	IDO2-001	ENSG00000188676	IDO2	6.28	1.53
ENST00000246549	FFAR2-201	ENSG00000126262	FFAR2	4.68	1.02
ENST00000370005	ELOVL3-001	ENSG00000119915	ELOVL3	5.39	0.99
ENST00000401630	IL6-005	ENSG00000136244	IL6	7.08	1.76
ENST00000222249	KCNN1-201	ENSG00000105642	KCNN1	5.85	1.69
ENST00000366578	ACTN2-001	ENSG00000077522	ACTN2	5.18	1.12
ENST00000392045	SP140-002	ENSG00000079263	SP140	8.90	3.15
ENST00000400497	RP11-37E23-001	ENSG00000215515	RP11-37E23.2	5.82	1.50
ENST00000413475	TMPRSS13-203	ENSG00000137747	TMPRSS13	7.34	2.07
ENST00000435131	IGF2BP3-202	ENSG00000136231	IGF2BP3	6.21	1.84
ENST00000457912	SMTNL1-202	ENSG00000214872	SMTNL1	4.59	1.69
ENST00000379965	TRIM22-001	ENSG00000132274	TRIM22	5.92	3.61
ENST00000477068	SP110-005	ENSG00000135899	SP110	3.83	0.32
ENST00000270458	CACNG8-001	ENSG00000142408	CACNG8	5.55	1.61
ENST00000494664	C3orf1-001	ENSG00000113845	C3orf1	8.79	2.68
ENST00000395135	PML-202	ENSG00000140464	PML	3.97	0.80
ENST00000306384	VAMP5-001	ENSG00000168899	VAMP5	4.72	1.20
ENST00000429934	RP11-383F6-001	ENSG00000229677	RP11-383F6.1	5.01	1.75
ENST00000343780	SEMA4D-001	ENSG00000187764	SEMA4D	6.11	3.04
ENST00000484704	HLA-F-012	ENSG00000204642	HLA-F	6.26	3.38
ENST00000360534	DPP4-001	ENSG00000197635	DPP4	6.28	2.51
ENST00000394506	CCL15-002	ENSG00000161574	CCL15	5.70	1.33
ENST00000376896	RORB-001	ENSG00000198963	RORB	4.70	0.79
ENST00000366457	AC023590-002	ENSG00000225885	AC023590.1	6.06	1.30
ENST00000261937	FLT4-001	ENSG00000037280	FLT4	4.70	0.95
ENST00000341855	RP11-760D2-001	ENSG00000213067	RP11-760D2.8	4.39	0.87
ENST00000477265	IL15-003	ENSG00000164136	IL15	5.27	1.96
ENST00000441380	IFITM4P-001	ENSG00000235821	IFITM4P	4.86	0.94
ENST00000443143	AC116049-201	ENSG00000236562	AC116049.2	4.15	0.63
ENST00000393743	DDX60-202	ENSG00000137628	DDX60	4.75	0.97
ENST00000422704	SEMA4D-005	ENSG00000187764	SEMA4D	4.34	0.80
ENST00000435786	PML-205	ENSG00000140464	PML	3.80	0.29
ENST00000367061	TAGAP-201	ENSG00000164691	TAGAP	4.40	0.90
ENST00000310706	JUP-001	ENSG00000173801	JUP	6.06	2.77
ENST00000414795	AC017076-002	ENSG00000225964	AC017076.4	4.98	1.17
ENST00000483358	NUB1-008	ENSG00000013374	NUB1	5.60	2.77
ENST00000299663	CLEC4E-201	ENSG00000166523	CLEC4E	4.26	0.85
ENST00000308521	APOBEC3F-001	ENSG00000128394	APOBEC3F	4.21	0.40
ENST00000413202	AC133644-004	ENSG00000222041	AC133644.3	7.50	2.26
ENST00000415816	IFI35-202	ENSG00000068079	IFI35	4.17	0.58
ENST00000223642	C5-001	ENSG00000106804	C5	4.15	0.74
ENST00000395641	NUPR1-201	ENSG00000176046	NUPR1	8.45	4.11
ENST00000399600	C17orf87-001	ENSG00000161929	C17orf87	4.06	0.66
ENST00000359092	AXL-202	ENSG00000167601	AXL	5.03	1.27
ENST00000499682	AC106881-201	ENSG00000247646	AC106881.1	6.08	1.18
ENST00000494150	APOBEC3G-007	ENSG00000239713	APOBEC3G	3.88	0.35
ENST00000491591	DPP4-010	ENSG00000197635	DPP4	5.12	1.77
ENST00000374045	TNFSF15-001	ENSG00000181634	TNFSF15	6.87	1.85
ENST00000298992	ABTB2-201	ENSG00000166016	ABTB2	3.69	0.42
ENST00000226299	LAP3-001	ENSG00000002549	LAP3	3.82	0.49
ENST00000357018	CACNA1A-202	ENSG00000141837	CACNA1A	5.35	1.94
ENST00000397709	MAP1LC3A-003	ENSG00000101460	MAP1LC3A	5.52	1.60
ENST00000415067	XXbac-BPG246D15-00	ENSG00000204261	XXbac-BPG246	4.40	1.89
ENST00000405885	IRF1-008	ENSG00000125347	IRF1	3.75	0.39
ENST00000337752	C5orf56-001	ENSG00000197536	C5orf56	3.70	0.45

ENST00000377508	OAS1-203	ENSG00000089127	OAS1	9.52	4.29
ENST00000301807	LBA1-201	ENSG00000168016	LBA1	4.34	0.74
ENST00000366140	AC017076-001	ENSG00000225964	AC017076.4	5.67	1.26
ENST00000450418	AC023480-001	ENSG00000229241	AC023480.1	4.62	1.20
ENST00000443093	AC116366-001	ENSG00000234290	AC116366.1	4.74	2.31
ENST00000235150	RNF19B-002	ENSG00000116514	RNF19B	3.32	0.15
ENST00000435224	ABTB2-001	ENSG00000166016	ABTB2	5.68	1.78
ENST00000467569	RIN2-005	ENSG00000132669	RIN2	7.69	1.99
ENST00000452357	OAS1-205	ENSG00000089127	OAS1	3.88	0.49
ENST00000433412	PHF15-203	ENSG00000043143	PHF15	4.86	2.40
ENST00000245907	C3-001	ENSG00000125730	C3	9.90	3.60
ENST00000370315	C6orf150-002	ENSG00000164430	C6orf150	3.47	0.34
ENST00000427236	RP4-641G12-001	ENSG00000238015	RP4-641G12.2	4.66	0.95
ENST00000304425	RP11-354P17-001	ENSG00000171889	RP11-354P17.[illegible]	5.22	1.49
ENST00000339867	RP3-391O22-001	ENSG00000196114	RP3-391O22.1	4.20	0.88
ENST00000268058	PML-001	ENSG00000140464	PML	3.69	0.37
ENST00000309186	FZD4-201	ENSG00000174804	FZD4	4.75	0.90
ENST00000303115	IL7R-001	ENSG00000168685	IL7R	3.72	0.45
ENST00000409112	SP100-004	ENSG00000067066	SP100	7.48	2.47
ENST00000239468	TNFSF18-201	ENSG00000120337	TNFSF18	5.78	1.51
ENST00000427581	LILRB1-007	ENSG00000104972	LILRB1	6.30	2.99
ENST00000412669	AC002456-003	ENSG00000223969	AC002456.1	5.69	1.27
ENST00000436078	RP11-91K9-001	ENSG00000231574	RP11-91K9.1	4.79	0.96
ENST00000456917	MIRHG2-001	ENSG00000234883	MIRHG2	4.12	0.80
ENST00000398738	C6orf138-001	ENSG00000244694	C6orf138	5.22	1.62
ENST00000299665	CLEC4D-201	ENSG00000166527	CLEC4D	4.21	1.27
ENST00000418263	CCNA1-201	ENSG00000133101	CCNA1	7.38	3.03
ENST00000460200	PSTPIP2-004	ENSG00000152229	PSTPIP2	3.80	1.17
ENST00000361820	GRIN3A-001	ENSG00000198785	GRIN3A	3.43	0.50
ENST00000456466	AC124319-203	ENSG00000180843	AC124319.2	4.25	0.87
ENST00000453265	PSMB9-201	ENSG00000240065	PSMB9	3.72	0.71
ENST00000414055	C1orf224-201	ENSG00000237276	C1orf224	6.00	1.45
ENST00000444639	KIAA0040-001	ENSG00000235750	KIAA0040	4.62	2.68
ENST00000485662	IFI44-007	ENSG00000137965	IFI44	4.64	0.92
ENST00000376753	FBXO6-001	ENSG00000116663	FBXO6	3.76	0.54
ENST00000356107	TJP1-201	ENSG00000104067	TJP1	5.41	2.49
ENST00000374897	TAP2-001	ENSG00000204267	TAP2	3.43	0.44
ENST00000446230	PIWIL4-202	ENSG00000134627	PIWIL4	4.48	2.37
ENST00000411702	AC124319-202	ENSG00000180843	AC124319.2	3.57	0.31
ENST00000343575	CXCL12-001	ENSG00000107562	CXCL12	3.93	1.15
ENST00000451096	RHEBL1-203	ENSG00000167550	RHEBL1	4.18	1.64
ENST00000371899	SLC2A6-001	ENSG00000160326	SLC2A6	4.21	1.11
ENST00000253110	C19orf66-201	ENSG00000130813	C19orf66	3.23	0.29
ENST00000499116	AL135914-201	ENSG00000248087	AL135914.3	4.15	0.72
ENST00000476223	RP11-383G6-001	ENSG00000240576	RP11-383G6.3	4.49	1.28
ENST00000056233	NFE2L3-001	ENSG00000050344	NFE2L3	3.85	0.76
ENST00000454686	RP1-193M11-001	ENSG00000213500	RP1-193M11.1	4.11	0.80
ENST00000233057	EIF2AK2-001	ENSG00000055332	EIF2AK2	3.38	0.41
ENST00000379375	EDN1-001	ENSG00000078401	EDN1	6.01	1.99
ENST00000462315	PARP9-003	ENSG00000138496	PARP9	3.83	0.65
ENST00000424614	OPTN-001	ENSG00000123240	OPTN	5.59	2.97
ENST00000374859	PSMB9-001	ENSG00000240065	PSMB9	3.52	0.43
ENST00000367066	TAGAP-001	ENSG00000164691	TAGAP	3.42	0.40
ENST00000467593	PSMB9-004	ENSG00000240065	PSMB9	4.47	2.11
ENST00000324873	NUPR1-001	ENSG00000176046	NUPR1	4.39	0.93
ENST00000478808	USP30-004	ENSG00000135093	USP30	3.82	0.70
ENST00000377507	TNFRSF9-001	ENSG00000049249	TNFRSF9	4.29	0.87

ENST00000420981	RP11-420G6-001	ENSG00000230438	RP11-420G6.1	4.07	0.87
ENST00000499886	AC092053-201	ENSG00000246007	AC092053.1	3.81	0.50
ENST00000378342	CCL4L2-201	ENSG00000197262	CCL4L2	7.96	2.30
ENST00000499648	AC009712-201	ENSG00000247829	AC009712.2	4.07	0.93
ENST00000433961	TRIM5-005	ENSG00000132256	TRIM5	3.77	0.86
ENST00000259339	TOR1B-001	ENSG00000136816	TOR1B	3.38	0.33
ENST00000447329	RP4-794H19-002	ENSG00000230812	RP4-794H19.4	4.26	1.17
ENST00000421011	IRF1-201	ENSG00000125347	IRF1	3.53	0.30
ENST00000422824	AC027228-201	ENSG00000229669	AC027228.1	5.33	1.33
ENST00000355055	IL28B-201	ENSG00000197110	IL28B	4.98	1.01
ENST00000369158	HIST2H3C-001	ENSG00000203811	HIST2H3C	3.89	0.70
ENST00000403683	HIST2H3A-001	ENSG00000203852	HIST2H3A	3.91	0.72
ENST00000331289	ASCL2-001	ENSG00000183734	ASCL2	3.49	0.67
ENST00000356865	ATP10A-201	ENSG00000206190	ATP10A	3.85	0.92
ENST00000454181	LYSMD2-201	ENSG00000140280	LYSMD2	4.20	1.04
ENST00000344300	C15orf48-001	ENSG00000166920	C15orf48	4.34	0.66
ENST00000355083	TMEM110-004	ENSG00000213533	TMEM110	3.34	0.34
ENST00000403662	CSF2RB-001	ENSG00000100368	CSF2RB	4.61	3.23
ENST00000461203	C5orf56-004	ENSG00000197536	C5orf56	4.31	1.36
ENST00000444414	CCL4L1-208	ENSG00000205020	CCL4L1	7.07	1.93
ENST00000438323	IFI35-203	ENSG00000068079	IFI35	5.30	3.57
ENST00000454398	HLA-DPA3-001	ENSG00000237398	HLA-DPA3	5.54	1.44
ENST00000442800	AC006027-002	ENSG00000244279	AC006027.2	5.36	2.35
ENST00000409930	IL1RN-005	ENSG00000136689	IL1RN	6.36	4.31
ENST00000372278	CD40-201	ENSG00000101017	CD40	3.41	0.35
ENST00000237289	TNFAIP3-001	ENSG00000118503	TNFAIP3	3.91	0.69
ENST00000437954	AC124319-201	ENSG00000237199	AC124319.3	3.56	0.55
ENST00000481958	APOBEC3G-003	ENSG00000239713	APOBEC3G	3.59	0.52
ENST00000492569	RP11-292E2-001	ENSG00000238755	RP11-292E2.3	3.96	0.81
ENST00000374429	CXCL12-002	ENSG00000107562	CXCL12	4.48	1.19
ENST00000421237	RP11-439L18-001	ENSG00000224460	RP11-439L18.2	4.21	0.72
ENST00000264246	CD80-001	ENSG00000121594	CD80	7.79	2.59
ENST00000396404	CYP19A1-202	ENSG00000137869	CYP19A1	5.51	1.75
ENST00000381799	RHOH-001	ENSG00000168421	RHOH	4.44	0.94
ENST00000395003	PHF15-002	ENSG00000043143	PHF15	3.28	0.45
ENST00000296545	IL15-001	ENSG00000164136	IL15	5.32	1.57
ENST00000418895	LGALS3BP-202	ENSG00000108679	LGALS3BP	3.55	0.89
ENST00000395761	CXCL1-201	ENSG00000163739	CXCL1	5.03	1.22
ENST00000499234	AC125257-201	ENSG00000247918	AC125257.1	8.00	2.62
ENST00000409746	PSTPIP2-002	ENSG00000152229	PSTPIP2	5.17	2.74
ENST00000269389	SECTM1-201	ENSG00000141574	SECTM1	3.69	0.53
ENST00000408936	DAB2IP-009	ENSG00000136848	DAB2IP	5.69	2.11
ENST00000409652	APOL6-001	ENSG00000221963	APOL6	3.24	0.45
ENST00000278505	ENDOD1-201	ENSG00000149218	ENDOD1	3.30	0.46
ENST00000470876	PHF15-004	ENSG00000043143	PHF15	3.73	1.76
ENST00000270443	ZNF702P-001	ENSG00000242779	ZNF702P	7.43	2.53
ENST00000394691	PLEKHG3-201	ENSG00000126822	PLEKHG3	4.78	2.49
ENST00000372886	PKIG-004	ENSG00000168734	PKIG	5.67	1.41
ENST00000309739	RND1-201	ENSG00000172602	RND1	4.14	1.12
ENST00000377712	AC092653-001	ENSG00000204872	AC092653.3	5.17	1.40
ENST00000368599	FAM26E-001	ENSG00000178033	FAM26E	5.83	1.39
ENST00000393739	DDX60-201	ENSG00000137628	DDX60	4.00	0.63
ENST00000380874	FOXC1-001	ENSG00000054598	FOXC1	4.23	1.20
ENST00000367055	SOD2-002	ENSG00000112096	SOD2	3.93	0.80
ENST00000502006	AC008063-201	ENSG00000246092	AC008063.2	3.66	0.66
ENST00000258381	SP110-002	ENSG00000135899	SP110	6.11	3.49
ENST00000382723	MSX1-001	ENSG00000163132	MSX1	5.39	1.56

ENST00000462451	VAMP5-002	ENSG00000168899	VAMP5	4.60	2.20
ENST00000431240	NOD2-201	ENSG00000167207	NOD2	4.23	1.41
ENST00000296161	DTX3L-001	ENSG00000163840	DTX3L	2.87	0.27
ENST00000273153	CSRNP1-001	ENSG00000144655	CSRNP1	4.19	0.72
ENST00000486332	TAP1-002	ENSG00000168394	TAP1	4.52	2.30
ENST00000501134	AC025574-201	ENSG00000247899	AC025574.1	3.50	0.89
ENST00000481066	PNPT1-005	ENSG00000138035	PNPT1	4.27	0.87
ENST00000427842	TNFSF15-201	ENSG00000181634	TNFSF15	5.09	1.59
ENST00000432272	RP13-297E16-002	ENSG00000223511	RP13-297E16.1	4.24	1.06
ENST00000393931	JUP-008	ENSG00000173801	JUP	4.78	1.14
ENST00000401899	RNF19B-202	ENSG00000116514	RNF19B	5.19	3.30
ENST00000409330	NBN-003	ENSG00000104320	NBN	4.73	3.11
ENST00000243346	NMI-001	ENSG00000123609	NMI	3.17	0.37
ENST00000500725	AL512428-201	ENSG00000245474	AL512428.1	4.37	0.89
ENST00000285013	SLFN13-201	ENSG00000154760	SLFN13	5.21	1.93
ENST00000447143	STAT4-201	ENSG00000138378	STAT4	6.22	3.21
ENST00000492382	PARP9-004	ENSG00000138496	PARP9	4.52	1.69
ENST00000200652	SLC22A4-001	ENSG00000197208	SLC22A4	3.38	0.71
ENST00000325719	OR52K2-201	ENSG00000181963	OR52K2	4.11	0.81
ENST00000425894	AC027119-001	ENSG00000229642	AC027119.1	4.11	0.97
ENST00000497857	RUFY4-004	ENSG00000188282	RUFY4	4.04	0.91
ENST00000448809	C9orf91-201	ENSG00000157693	C9orf91	3.04	0.44
ENST00000395113	UBE2L6-203	ENSG00000156587	UBE2L6	2.87	0.18
ENST00000371697	ANKRD1-001	ENSG00000148677	ANKRD1	6.13	1.86
ENST00000423390	BX255923-001	ENSG00000240907	BX255923.2	6.02	1.73
ENST00000337530	KIAA1958-001	ENSG00000165185	KIAA1958	3.26	0.58
ENST00000300797	PRRT2-201	ENSG00000167371	PRRT2	3.94	1.04
ENST00000370466	GBP2-002	ENSG00000162645	GBP2	4.23	2.83
ENST00000415826	PLA2G16-203	ENSG00000176485	PLA2G16	6.15	2.40
ENST00000355014	SEMA4A-002	ENSG00000196189	SEMA4A	3.55	0.76
ENST00000245457	PTGER2-001	ENSG00000125384	PTGER2	3.55	0.54
ENST00000306139	ATF5-201	ENSG00000169136	ATF5	3.72	0.81
ENST00000420301	CYP19A1-203	ENSG00000137869	CYP19A1	4.48	1.35
ENST00000428917	TAP2-201	ENSG00000204267	TAP2	3.10	0.57
ENST00000379882	DDX58-201	ENSG00000107201	DDX58	5.44	1.28
ENST00000361171	FAM125B-001	ENSG00000196814	FAM125B	3.10	0.36
ENST00000477775	ATXN7L1-008	ENSG00000146776	ATXN7L1	5.66	2.03
ENST00000369583	DUSP-001	ENSG00000138166	DUSP	3.19	0.49
ENST00000373645	SP140-003	ENSG00000079263	SP140	4.02	0.88
ENST00000468472	MUSTN1-004	ENSG00000243696	MUSTN1	3.34	0.37
ENST00000361157	IFI6-001	ENSG00000126709	IFI6	3.40	0.56
ENST00000260282	FXYD6-201	ENSG00000137726	FXYD6	3.15	0.46
ENST00000294997	CR1L-001	ENSG00000197721	CR1L	5.30	1.42
ENST00000243440	BATF3-001	ENSG00000123685	BATF3	3.85	1.31
ENST00000396650	C15orf48-201	ENSG00000166920	C15orf48	3.84	0.63
ENST00000394627	CCL7-003	ENSG00000108688	CCL7	6.16	2.28
ENST00000236147	SELL-001	ENSG00000188404	SELL	3.45	0.56
ENST00000302326	MN1-001	ENSG00000169184	MN1	3.78	0.96
ENST00000263464	BIRC3-201	ENSG00000023445	BIRC3	3.30	0.54
ENST00000399817	IFITM2-202	ENSG00000185201	IFITM2	3.07	0.50
ENST00000392981	XRN1-004	ENSG00000114127	XRN1	3.19	0.57
ENST00000267015	GPR84-201	ENSG00000139572	GPR84	2.91	0.33
ENST00000395148	CFLAR-005	ENSG00000003402	CFLAR	2.93	0.31
ENST00000371752	ZNFX1-001	ENSG00000124201	ZNFX1	2.97	0.40
ENST00000458061	C1R-202	ENSG00000159403	C1R	5.40	1.57
ENST00000314553	EXOC3L-201	ENSG00000179044	EXOC3L	4.27	1.52
ENST00000441767	PRICKLE1-202	ENSG00000139174	PRICKLE1	5.10	1.34

ENST00000262776	LGALS3BP-201	ENSG00000108679	LGALS3BP	3.27	0.65
ENST00000314128	STAT2-201	ENSG00000170581	STAT2	3.27	0.54
ENST00000498904	AL160008-201	ENSG00000247275	AL160008.2	4.61	1.48
ENST00000490682	CASP8-008	ENSG00000064012	CASP8	3.19	0.52
ENST00000266542	C1RL-201	ENSG00000139178	C1RL	4.25	1.62
ENST00000437695	RP5-1141O19-001	ENSG00000227085	RP5-1141O19.	3.69	0.66
ENST00000221847	EBI3-201	ENSG00000105246	EBI3	4.87	1.39
ENST00000438195	RP4-794H19-001	ENSG00000230812	RP4-794H19.4	4.50	1.39
ENST00000367128	FAM72A-001	ENSG00000196550	FAM72A	3.78	2.00
ENST00000495552	MUSTN1-003	ENSG00000243696	MUSTN1	3.05	0.50
ENST00000456298	FAM177B-005	ENSG00000197520	FAM177B	3.67	0.66
ENST00000264492	CXCL2-201	ENSG00000081041	CXCL2	3.63	1.01
ENST00000259939	RNF144B-001	ENSG00000137393	RNF144B	3.02	0.50
ENST00000489597	SP110-012	ENSG00000135899	SP110	4.88	2.77
ENST00000342771	AUTS2-001	ENSG00000158321	AUTS2	4.34	1.91
ENST00000383320	HCP5-201	ENSG00000206337	HCP5	4.41	1.73
ENST00000355295	TDRD7-001	ENSG00000196116	TDRD7	3.95	1.89
ENST00000252785	SCO2-002	ENSG00000130489	SCO2	6.61	4.24
ENST00000383660	PARP15-201	ENSG00000173200	PARP15	5.48	2.49
ENST00000486194	HLA-F-010	ENSG00000204642	HLA-F	6.65	3.60
ENST00000456775	ST7OT1-001	ENSG00000227199	ST7OT1	3.63	1.16
ENST00000245414	IRF1-001	ENSG00000125347	IRF1	3.48	0.72
ENST00000336301	RNF213-201	ENSG00000173821	RNF213	4.44	1.13
ENST00000417218	AL592494-001	ENSG00000227082	AL592494.3	3.48	0.92
ENST00000392068	SGPP2-201	ENSG00000163082	SGPP2	4.35	1.77
ENST00000412615	TRAFD1-202	ENSG00000135148	TRAFD1	2.60	0.24
ENST00000485978	SLC2A6-003	ENSG00000160326	SLC2A6	4.59	2.22
ENST00000278175	ADM-201	ENSG00000148926	ADM	3.01	0.38
ENST00000434136	AC124319-201	ENSG00000234591	AC124319.1	3.21	0.50
ENST00000482294	CBX7-002	ENSG00000100307	CBX7	2.89	0.41
ENST00000273582	KIAA0226-001	ENSG00000145016	KIAA0226	2.87	0.38
ENST00000470178	CFLAR-022	ENSG00000003402	CFLAR	4.89	3.00
ENST00000260126	SLCO5A1-201	ENSG00000137571	SLCO5A1	4.74	1.39
ENST00000366134	RP13-314C10-001	ENSG00000233785	RP13-314C10.[illegible]	4.58	1.56
ENST00000428912	RP5-908D6-001	ENSG00000233235	RP5-908D6.1	3.31	0.72
ENST00000418839	RP11-408H1-003	ENSG00000223387	RP11-408H1.2	4.59	1.32
ENST00000467626	CRLF2-002	ENSG00000205755	CRLF2	3.80	1.12
ENST00000354922	PNRC1-002	ENSG00000146278	PNRC1	6.09	2.67
ENST00000245903	CD70-201	ENSG00000125726	CD70	5.06	1.55
ENST00000373177	HDX-001	ENSG00000165259	HDX	4.06	1.04
ENST00000282045	PSTPIP2-201	ENSG00000152229	PSTPIP2	7.43	3.38
ENST00000366489	C1orf150-002	ENSG00000169224	C1orf150	4.50	1.19
ENST00000336032	PNRC1-001	ENSG00000146278	PNRC1	3.00	0.51
ENST00000373451	FTSJD2-001	ENSG00000137200	FTSJD2	2.74	0.34
ENST00000323646	PLA2G16-201	ENSG00000176485	PLA2G16	5.40	2.21
ENST00000358752	FUT4-201	ENSG00000196371	FUT4	3.00	0.44
ENST00000369155	HIST2H2BE-001	ENSG00000184678	HIST2H2BE	2.88	0.54
ENST00000429054	RNF144B-202	ENSG00000137393	RNF144B	2.80	0.37
ENST00000340023	FPR2-201	ENSG00000171049	FPR2	3.89	1.08
ENST00000423313	KIAA0040-002	ENSG00000235750	KIAA0040	8.54	3.02
ENST00000442824	AC116347-201	ENSG00000238000	AC116347.1	2.93	0.43
ENST00000375940	MASTL-004	ENSG00000120539	MASTL	7.78	3.34
ENST00000371897	SLC2A6-002	ENSG00000160326	SLC2A6	4.40	2.87
ENST00000215886	LGALS2-001	ENSG00000100079	LGALS2	4.61	1.55
ENST00000381652	JAK2-001	ENSG00000096968	JAK2	3.48	0.92
ENST00000374155	RUFY4-002	ENSG00000188282	RUFY4	5.43	2.07
ENST00000493136	ITIH3-003	ENSG00000162267	ITIH3	5.15	1.66

ENST00000382848	GJB2-001	ENSG00000165474	GJB2	3.67	1.01
ENST00000468624	MOV10-007	ENSG00000155363	MOV10	2.81	0.32
ENST00000493645	PARP15-006	ENSG00000173200	PARP15	4.32	2.37
ENST00000449067	FBXO6-003	ENSG00000116663	FBXO6	2.95	0.40
ENST00000260184	DDX60L-201	ENSG00000181381	DDX60L	3.65	0.91
ENST00000431231	ARHGAP23-201	ENSG00000225485	ARHGAP23	3.69	0.73
ENST00000329281	BLZF1-002	ENSG00000117475	BLZF1	3.04	0.56
ENST00000483055	NTNG2-005	ENSG00000196358	NTNG2	4.48	1.31
ENST00000373961	IFI6-202	ENSG00000126709	IFI6	3.57	0.83
ENST00000457488	DAPK2-001	ENSG00000035664	DAPK2	4.43	1.17
ENST00000353739	RNF38-002	ENSG00000137075	RNF38	6.68	1.96
ENST00000267838	LYSMD2-001	ENSG00000140280	LYSMD2	4.98	2.60
ENST00000501838	AL109616-201	ENSG00000246147	AL109616.1	3.69	0.94
ENST00000369175	FAM72C-001	ENSG00000203817	FAM72C	3.18	0.56
ENST00000374007	ASAH2B-002	ENSG00000204147	ASAH2B	3.38	0.70
ENST00000396847	TRIM5-004	ENSG00000132256	TRIM5	5.62	3.06
ENST00000463000	WDR86-005	ENSG00000187260	WDR86	3.95	1.22
ENST00000229634	NCOA7-201	ENSG00000111912	NCOA7	7.97	4.06
ENST00000284110	HS3ST3A1-001	ENSG00000153976	HS3ST3A1	4.66	1.08
ENST00000330871	SOCS3-201	ENSG00000184557	SOCS3	3.64	0.84
ENST00000500570	AC008875-201	ENSG00000245900	AC008875.2	2.67	0.34
ENST00000264832	ICAM1-201	ENSG00000090339	ICAM1	3.23	0.55
ENST00000393113	AC073655-201	ENSG00000213250	AC073655.1	3.11	0.57
ENST00000373887	TRAF1-001	ENSG00000056558	TRAF1	4.53	1.08
ENST00000422211	CCL3L1-001	ENSG00000205021	CCL3L1	5.34	1.27
ENST00000402854	RP11-367G6-001	ENSG00000220517	RP11-367G6.2	3.26	0.86
ENST00000444269	CR1L-201	ENSG00000197721	CR1L	5.22	1.40
ENST00000367808	BLZF1-001	ENSG00000117475	BLZF1	3.35	0.79
ENST00000315768	C17orf87-201	ENSG00000161929	C17orf87	3.89	0.85
ENST00000334409	PDCD1-001	ENSG00000188389	PDCD1	3.97	1.88
ENST00000320578	RAB39-201	ENSG00000179331	RAB39	3.37	0.91
ENST00000420252	AC013470-001	ENSG00000236048	AC013470.3	2.68	0.28
ENST00000369159	HIST2H2AA4-001	ENSG00000203812	HIST2H2AA4	3.43	0.71
ENST00000500836	AC010319-201	ENSG00000244887	AC010319.2	2.58	0.35
ENST00000409397	SPATS2L-023	ENSG00000196141	SPATS2L	7.29	2.96
ENST00000455793	RP11-3D23-001	ENSG00000236852	RP11-3D23.1	2.41	0.20
ENST00000278940	TMPRSS13-201	ENSG00000137747	TMPRSS13	5.22	2.21
ENST00000473341	PARP12-002	ENSG00000059378	PARP12	2.98	0.67
ENST00000369161	HIST2H2AA3-001	ENSG00000183558	HIST2H2AA3	3.42	0.71
ENST00000501526	AL035530-201	ENSG00000246620	AL035530.1	3.16	0.59
ENST00000322282	GRAMD1B-201	ENSG00000023171	GRAMD1B	3.57	1.82
ENST00000269209	FAM59A-002	ENSG00000141441	FAM59A	5.06	2.20
ENST00000311601	SH3PXD2B-201	ENSG00000174705	SH3PXD2B	2.78	0.51
ENST00000426906	C22orf28-201	ENSG00000100220	C22orf28	2.58	0.31
ENST00000337387	WTAP-002	ENSG00000146457	WTAP	3.04	0.57
ENST00000452637	RP11-403I13-001	ENSG00000226067	RP11-403I13.7	2.93	0.76
ENST00000460961	CFLAR-024	ENSG00000003402	CFLAR	2.62	0.41
ENST00000256015	BTG1-201	ENSG00000133639	BTG1	2.53	0.26
ENST00000367054	SOD2-201	ENSG00000112096	SOD2	4.80	2.72
ENST00000471700	PSME2-003	ENSG00000100911	PSME2	2.94	0.73
ENST00000357443	MOV10-005	ENSG00000155363	MOV10	5.64	3.74
ENST00000453033	RP11-278A16-001	ENSG00000225131	RP11-278A16.:	2.99	0.52
ENST00000268459	NKD1-001	ENSG00000140807	NKD1	3.14	1.14
ENST00000439871	IL8RBP-002	ENSG00000229754	IL8RBP	3.15	0.73
ENST00000297977	HDX-201	ENSG00000165259	HDX	4.37	1.63
ENST00000261366	LMNB1-001	ENSG00000113368	LMNB1	2.84	0.50
ENST00000279249	CDC42EP2-201	ENSG00000149798	CDC42EP2	3.58	0.85

ENST00000502167	AC145210-202	ENSG00000247317	AC145210.1	4.26	1.44
ENST00000473240	GCA-013	ENSG00000115271	GCA	5.42	2.36
ENST00000476513	APOBEC3F-004	ENSG00000128394	APOBEC3F	3.25	0.84
ENST00000369173	AL358813-201	ENSG00000203815	AL358813.2	3.18	0.90
ENST00000372285	CD40-001	ENSG00000101017	CD40	3.17	0.69
ENST00000296026	CXCL3-201	ENSG00000163734	CXCL3	4.28	1.13
ENST00000262510	NLRC5-001	ENSG00000140853	NLRC5	2.63	0.69
ENST00000378947	C5orf56-003	ENSG00000197536	C5orf56	4.02	1.38
ENST00000400889	FAM72D-001	ENSG00000215784	FAM72D	2.78	0.35
ENST00000296414	DAPP1-201	ENSG00000070190	DAPP1	2.94	0.71
ENST00000435315	AF127936-001	ENSG00000226751	AF127936.4	4.52	1.86
ENST00000288466	ZNF618-201	ENSG00000157657	ZNF618	2.76	0.39
ENST00000394613	PLA2G16-202	ENSG00000176485	PLA2G16	4.52	2.18
ENST00000242351	ZC3HAV1-001	ENSG00000105939	ZC3HAV1	3.27	0.72
ENST00000300589	NOD2-001	ENSG00000167207	NOD2	3.43	0.95
ENST00000453177	RP11-370B11-001	ENSG00000236739	RP11-370B11.:	2.67	0.50
ENST00000367797	F5-001	ENSG00000198734	F5	3.36	0.74
ENST00000498457	RP11-292E2-001	ENSG00000241220	RP11-292E2.2	4.09	1.29
ENST00000454907	RP3-497J21-001	ENSG00000235008	RP3-497J21.1	4.62	1.75
ENST00000435374	MAP2K6-202	ENSG00000108984	MAP2K6	4.95	2.18
ENST00000290349	CBR1-001	ENSG00000159228	CBR1	2.44	0.19
ENST00000255688	RARRES3-201	ENSG00000133321	RARRES3	6.03	3.15
ENST00000255465	CCNA1-001	ENSG00000133101	CCNA1	5.65	3.15
ENST00000330243	IRF7-001	ENSG00000185507	IRF7	3.57	1.14
ENST00000354115	IL1RN-002	ENSG00000136689	IL1RN	3.28	0.72
ENST00000501717	AC061992-201	ENSG00000245152	AC061992.4	3.52	0.96
ENST00000416879	AC010930-201	ENSG00000225101	AC010930.2	3.88	0.87
ENST00000446848	SNX10-201	ENSG00000086300	SNX10	3.23	0.68
ENST00000382215	AC233309-201	ENSG00000205885	AC233309.1	3.80	1.21
ENST00000265807	SH2D4A-001	ENSG00000104611	SH2D4A	3.59	1.03
ENST00000368121	DARC-002	ENSG00000213088	DARC	4.44	1.79
ENST00000453010	C9orf109-001	ENSG00000231528	C9orf109	2.46	0.29
ENST00000227752	IL10RA-201	ENSG00000110324	IL10RA	2.61	0.37
ENST00000258534	DRAM1-201	ENSG00000136048	DRAM1	2.37	0.40
ENST00000454730	HPSE-201	ENSG00000173083	HPSE	2.93	0.56
ENST00000355338	WARS-202	ENSG00000140105	WARS	6.85	4.88
ENST00000316043	AC007536-201	ENSG00000176654	AC007536.1	4.44	1.28
ENST00000499282	AL160471-201	ENSG00000246064	AL160471.2	3.37	1.04
ENST00000368089	KCNJ10-001	ENSG00000177807	KCNJ10	5.11	1.33
ENST00000302035	SLAMF1-001	ENSG00000117090	SLAMF1	5.08	1.86
ENST00000452222	RP11-428O18-001	ENSG00000230641	RP11-428O18.	5.27	1.84
ENST00000436911	TRGC2-001	ENSG00000227191	TRGC2	3.19	0.88
ENST00000464885	BCL2L14-004	ENSG00000121380	BCL2L14	5.76	1.55
ENST00000413796	AC002511-001	ENSG00000233214	AC002511.4	3.95	1.05
ENST00000231228	IL12B-001	ENSG00000113302	IL12B	4.71	1.55
ENST00000367089	DYNLT1-001	ENSG00000146425	DYNLT1	2.29	0.22
ENST00000450096	PNPT1-201	ENSG00000138035	PNPT1	3.42	2.34
ENST00000374291	STMN1-005	ENSG00000117632	STMN1	-3.19	0.95
ENST00000441742	AC115284-201	ENSG00000233341	AC115284.1	-2.64	0.46
ENST00000393203	PTGFRN-001	ENSG00000134247	PTGFRN	-3.17	0.93
ENST00000393423	RGL3-203	ENSG00000205517	RGL3	-4.06	2.54
ENST00000361170	IQGAP3-001	ENSG00000183856	IQGAP3	-2.95	0.68
ENST00000330777	AC068353-201	ENSG00000182319	AC068353.1	-2.40	0.41
ENST00000270645	RCN3-201	ENSG00000142552	RCN3	-3.20	0.79
ENST00000396123	GREB1-209	ENSG00000196208	GREB1	-2.81	0.45
ENST00000464213	CD36-014	ENSG00000135218	CD36	-7.77	5.32
ENST00000225538	P2RX1-201	ENSG00000108405	P2RX1	-2.62	0.35

ENST00000490823	KHK-003	ENSG00000138030	KHK	-3.74	1.56
ENST00000329099	FAM101B-001	ENSG00000183688	FAM101B	-2.49	0.23
ENST00000342032	C7orf16-001	ENSG00000106341	C7orf16	-4.44	1.19
ENST00000455981	RP11-344B5-001	ENSG00000224307	RP11-344B5.3	-4.20	1.16
ENST00000319340	CHST13-201	ENSG00000180767	CHST13	-2.76	0.50
ENST00000354698	ZNF589-001	ENSG00000164048	ZNF589	-3.69	2.21
ENST00000402125	PROC-012	ENSG00000115718	PROC	-3.62	1.24
ENST00000481799	GGTA1-005	ENSG00000204136	GGTA1	-3.46	1.20
ENST00000501995	AL139158-201	ENSG00000244884	AL139158.1	-3.60	1.27
ENST00000453888	PARVG-204	ENSG00000138964	PARVG	-3.03	0.48
ENST00000409345	ADRA2B-001	ENSG00000222040	ADRA2B	-3.80	0.69
ENST00000241356	ADORA3-010	ENSG00000121933	ADORA3	-4.18	1.20
ENST00000276420	DOK2-001	ENSG00000147443	DOK2	-3.38	0.62
ENST00000373176	AK1-008	ENSG00000106992	AK1	-3.13	0.53
ENST00000494322	SLC11A1-010	ENSG00000018280	SLC11A1	-5.26	3.43
ENST00000299164	ADAMTS15-201	ENSG00000166106	ADAMTS15	-4.39	1.22
ENST00000468508	SLC19A1-011	ENSG00000173638	SLC19A1	-4.36	1.66
ENST00000388827	CCDC152-202	ENSG00000198865	CCDC152	-5.46	2.60
ENST00000377008	C1orf127-001	ENSG00000175262	C1orf127	-4.66	1.90
ENST00000339788	DAB2-202	ENSG00000153071	DAB2	-6.74	4.76
ENST00000413146	TNFRSF8-202	ENSG00000120949	TNFRSF8	-4.61	1.77
ENST00000331664	C2orf71-001	ENSG00000179270	C2orf71	-3.84	1.13
ENST00000374292	STMN1-006	ENSG00000117632	STMN1	-4.91	2.24
ENST00000318430	TMC8-202	ENSG00000167895	TMC8	-3.20	0.52
ENST00000264741	ITGA9-001	ENSG00000144668	ITGA9	-5.72	1.51
ENST00000335281	GNG2-201	ENSG00000186469	GNG2	-4.06	1.16
ENST00000248706	RASL11B-001	ENSG00000128045	RASL11B	-3.21	0.38
ENST00000377103	THBD-001	ENSG00000178726	THBD	-4.78	1.03
ENST00000311124	SLC19A1-001	ENSG00000173638	SLC19A1	-3.55	0.86
ENST00000367460	RGS18-001	ENSG00000150681	RGS18	-4.77	1.40
ENST00000423345	PRAM1-202	ENSG00000133246	PRAM1	-4.31	2.74
ENST00000442811	AC104809-002	ENSG00000226321	AC104809.2	-5.10	1.25
ENST00000479024	NT5DC2-004	ENSG00000168268	NT5DC2	-7.43	1.77
ENST00000318074	SYTL1-001	ENSG00000142765	SYTL1	-4.85	1.55
ENST00000502226	AL033528-201	ENSG00000247440	AL033528.1	-5.17	1.81
ENST00000399815	IFITM1-202	ENSG00000185885	IFITM1	-4.63	0.66
ENST00000438506	AC104809-003	ENSG00000233392	AC104809.3	-4.79	0.85
ENST00000356289	AMICA1-202	ENSG00000160593	AMICA1	-4.27	0.77
ENST00000221347	FCGBP-201	ENSG00000090920	FCGBP	-4.26	0.73
ENST00000430980	AC104809-201	ENSG00000226321	AC104809.2	-5.29	1.13
ENST00000294435	RBP7-001	ENSG00000162444	RBP7	-5.97	1.59
ENST00000216180	PNPLA3-001	ENSG00000100344	PNPLA3	-5.95	2.12
ENST00000427494	APOBEC3D-202	ENSG00000243811	APOBEC3D	-4.35	0.38
ENST00000441301	F13A1-203	ENSG00000124491	F13A1	-6.73	2.01
ENST00000246006	CD93-002	ENSG00000125810	CD93	-6.21	1.30
ENST00000428281	FCGBP-202	ENSG00000090920	FCGBP	-5.45	1.54
ENST00000412757	DPEP2-203	ENSG00000167261	DPEP2	-7.10	1.44
ENST00000418218	AC104809-002	ENSG00000233392	AC104809.3	-6.38	1.32
ENST00000501836	AC067945-201	ENSG00000245033	AC067945.2	-7.60	0.87
ENST00000251507	RABGAP1L-001	ENSG00000152061	RABGAP1L	-7.66	0.55
ENST00000391824	NUP62-202	ENSG00000213024	NUP62	-8.25	0.48
