# Supplementary material for: Identification of Genes Critical for Resistance to Infection by West Nile Virus Using RNA-Seq Analysis
Source: Viruses. 2013 Jul 8;5(7):1664–81. doi: 10.3390/v5071664 (PMC3738954; doi:10.3390/v5071664)
Supplement: Supplementary File 1 — Supplementary (ZIP, 454 KB) [file viruses-05-01664-s001.zip › Qian_Table S3. Differentially expressed transcripts in WNV infected macrophages.pdf]

Table S3. Differentially expressed transcripts in WNV infected macrophages

Ensembl.Transcript.ID	Transcript.Name	Ensembl.Gene.ID	Gene.Name	Fold change estimated	
				log2 fold change	SD
ENST00000479361	C1orf170-002	ENSG00000187642	C1orf170	3.91	1.94
ENST00000304952	HES4-001	ENSG00000188290	HES4	6.66	2.04
ENST00000379389	ISG15-001	ENSG00000187608	ISG15	7.85	1.00
ENST00000378625	NADK-001	ENSG00000008130	NADK	2.13	0.53
ENST00000471840	MMEL1-002	ENSG00000142606	MMEL1	-2.97	3.10
ENST00000481789	KCNAB2-011	ENSG00000069424	KCNAB2	-2.43	3.82
ENST00000377507	TNFRSF9-001	ENSG00000049249	TNFRSF9	4.14	0.87
ENST00000294435	RBP7-001	ENSG00000162444	RBP7	-5.15	1.59
ENST00000447850	CASZ1-003	ENSG00000130940	CASZ1	2.64	4.20
ENST00000476357	C1orf127-002	ENSG00000175262	C1orf127	-3.21	1.77
ENST00000377008	C1orf127-001	ENSG00000175262	C1orf127	-3.92	1.90
ENST00000376753	FBXO6-001	ENSG00000116663	FBXO6	3.66	0.54
ENST00000449067	FBXO6-003	ENSG00000116663	FBXO6	2.90	0.40
ENST00000476309	AGTRAP-006	ENSG00000177674	AGTRAP	2.81	3.85
ENST00000413146	TNFRSF8-202	ENSG00000120949	TNFRSF8	-3.74	1.77
ENST00000416696	RP5-888M10-001	ENSG00000229484	RP5-888M10.1	-3.16	0.97
ENST00000375408	ARHGEF10L-002	ENSG00000074964	ARHGEF10L	2.50	3.61
ENST00000375071	CDA-001	ENSG00000158825	CDA	-2.86	1.35
ENST00000461985	CDA-002	ENSG00000158825	CDA	-3.16	2.21
ENST00000400490	PINK1-003	ENSG00000158828	PINK1	-2.42	0.98
ENST00000400191	EPHB2-001	ENSG00000133216	EPHB2	2.10	3.86
ENST00000500280	AL021154-201	ENSG00000246830	AL021154.6	-3.52	1.39
ENST00000361729	E2F2-001	ENSG00000007968	E2F2	-2.90	1.72
ENST00000463312	ID3-003	ENSG00000117318	ID3	-2.77	3.75
ENST00000374292	STMN1-006	ENSG00000117632	STMN1	-4.02	2.24
ENST00000374291	STMN1-005	ENSG00000117632	STMN1	-2.99	0.95
ENST00000502226	AL033528-201	ENSG00000247440	AL033528.1	-4.33	1.81
ENST00000430292	PIGV-003	ENSG00000060642	PIGV	2.26	2.86
ENST00000361157	IFI6-001	ENSG00000126709	IFI6	3.33	0.56
ENST00000373961	IFI6-202	ENSG00000126709	IFI6	3.58	0.83
ENST00000481487	FNDC5-003	ENSG00000160097	FNDC5	-2.95	1.98
ENST00000291421	FNDC5-201	ENSG00000160097	FNDC5	-3.51	2.40
ENST00000401899	RNF19B-202	ENSG00000116514	RNF19B	4.02	3.30
ENST00000235150	RNF19B-002	ENSG00000116514	RNF19B	3.33	0.15
ENST00000373087	ZC3H12A-001	ENSG00000163874	ZC3H12A	3.02	0.71
ENST00000492829	ZC3H12A-003	ENSG00000163874	ZC3H12A	2.37	0.74
ENST00000472312	ZC3H12A-004	ENSG00000163874	ZC3H12A	3.01	2.48
ENST00000501995	AL139158-201	ENSG00000244884	AL139158.1	-3.14	1.27
ENST00000371761	CDKN2C-002	ENSG00000123080	CDKN2C	2.30	2.00
ENST00000488036	SLC1A7-003	ENSG00000162383	SLC1A7	-2.61	1.42
ENST00000498228	ACOT11-004	ENSG00000162390	ACOT11	-2.51	2.15
ENST00000371316	ACOT11-001	ENSG00000162390	ACOT11	-2.76	0.72
ENST00000302250	FAM151A-001	ENSG00000162391	FAM151A	-3.56	2.40
ENST00000416119	RP11-240D10-001	ENSG00000230728	RP11-240D10.1	-3.17	1.15
ENST00000357977	NFIA-008	ENSG00000162599	NFIA	-2.40	0.81
ENST00000329654	PDE4B-001	ENSG00000184588	PDE4B	6.13	2.01
ENST00000480732	NEXN-004	ENSG00000162614	NEXN	3.16	2.18
ENST00000370763	DNAJB4-001	ENSG00000162616	DNAJB4	2.82	0.81
ENST00000370747	IFI44-001	ENSG00000137965	IFI44	6.52	1.99
ENST00000485662	IFI44-007	ENSG00000137965	IFI44	4.75	0.92
ENST00000284027	MCOLN2-001	ENSG00000153898	MCOLN2	5.83	2.39
ENST00000489444	GBP3-002	ENSG00000117226	GBP3	4.62	2.91
ENST00000461384	GBP3-007	ENSG00000117226	GBP3	4.39	1.85
ENST00000498904	AL160008-201	ENSG00000247275	AL160008.2	4.57	1.48
ENST00000370473	GBP1-001	ENSG00000117228	GBP1	5.91	0.82
ENST00000370466	GBP2-002	ENSG00000162645	GBP2	3.43	2.83
ENST00000493802	GBP2-004	ENSG00000162645	GBP2	2.67	0.70
ENST00000355754	GBP4-001	ENSG00000162654	GBP4	7.97	0.98
ENST00000481397	GBP4-002	ENSG00000162654	GBP4	7.37	5.46
ENST00000370459	GBP5-001	ENSG00000154451	GBP5	6.59	1.53
ENST00000394662	RP4-644F6-001	ENSG00000225492	RP4-644F6.2	6.50	0.35
ENST00000500375	AL355145-201	ENSG00000246743	AL355145.1	-2.94	2.17
ENST00000485317	KCNA2-002	ENSG00000177301	KCNA2	-3.02	1.24
ENST00000241356	ADORA3-010	ENSG00000121933	ADORA3	-3.61	1.20
ENST00000357260	C1orf183-001	ENSG00000197852	C1orf183	-2.76	0.55
ENST00000369645	MOV10-006	ENSG00000155363	MOV10	2.52	0.68
ENST00000468624	MOV10-007	ENSG00000155363	MOV10	2.81	0.32
ENST00000357443	MOV10-005	ENSG00000155363	MOV10	3.82	3.74

ENST00000421943	RP11-426L16-001	ENSG00000225075	RP11-426L16.3	2.57	1.48
ENST00000393203	PTGFRN-001	ENSG00000134247	PTGFRN	-3.21	0.93
ENST00000369448	FAM46C-001	ENSG00000183508	FAM46C	2.33	1.35
ENST00000401014	NBPF7-201	ENSG00000215864	NBPF7	3.17	1.25
ENST00000480765	FAM72B-007	ENSG00000188610	FAM72B	2.10	0.53
ENST00000369390	FAM72B-001	ENSG00000188610	FAM72B	3.13	2.23
ENST00000437515	AL358175-002	ENSG00000230806	AL358175.1	2.50	0.91
ENST00000304465	SRGAP2P1-001	ENSG00000171943	SRGAP2P1	2.75	0.98
ENST00000417218	AL592494-001	ENSG00000227082	AL592494.3	2.83	0.92
ENST00000432384	BX284650-006	ENSG00000231734	BX284650.6	2.13	1.21
ENST00000400889	FAM72D-001	ENSG00000215784	FAM72D	2.82	0.35
ENST00000467933	SRGAP2P2-002	ENSG00000196369	SRGAP2P2	2.28	0.24
ENST00000491897	SRGAP2P2-001	ENSG00000196369	SRGAP2P2	2.97	3.84
ENST00000442509	AL592284-003	ENSG00000236943	AL592284.2	2.31	0.76
ENST00000369304	ITGA10-001	ENSG00000143127	ITGA10	2.55	0.88
ENST00000438469	RP11-325P15-001	ENSG00000230832	RP11-325P15.2	2.70	0.57
ENST00000443058	RP11-325P15-201	ENSG00000230832	RP11-325P15.2	2.34	0.42
ENST00000271348	GJA5-201	ENSG00000143140	GJA5	-2.99	1.63
ENST00000445243	RP11-495P10-001	ENSG00000231551	RP11-495P10.5	2.23	1.37
ENST00000452637	RP11-403I13-001	ENSG00000226067	RP11-403I13.7	2.46	0.76
ENST00000433084	RP11-403I13-001	ENSG00000235999	RP11-403I13.6	2.42	0.37
ENST00000369175	FAM72C-001	ENSG00000203817	FAM72C	3.02	0.56
ENST00000443602	RP11-353N4-001	ENSG00000223759	RP11-353N4.4	2.38	0.45
ENST00000369173	AL358813-201	ENSG00000203815	AL358813.2	2.72	0.90
ENST00000359348	AL358813-201	ENSG00000197844	AL358813.5	2.20	0.84
ENST00000369167	HIST2H2BF-001	ENSG00000203814	HIST2H2BF	2.52	0.77
ENST00000392939	HIST2H4A-003	ENSG00000183941	HIST2H4A	2.77	0.58
ENST00000369165	HIST2H4A-001	ENSG00000183941	HIST2H4A	2.99	1.11
ENST00000369158	HIST2H3C-001	ENSG00000203811	HIST2H3C	3.61	0.70
ENST00000369161	HIST2H2AA3-001	ENSG00000183558	HIST2H2AA3	3.39	0.71
ENST00000369159	HIST2H2AA4-001	ENSG00000203812	HIST2H2AA4	3.42	0.71
ENST00000403683	HIST2H3A-001	ENSG00000203852	HIST2H3A	3.63	0.72
ENST00000369157	HIST2H4B-001	ENSG00000182217	HIST2H4B	2.95	1.37
ENST00000392932	HIST2H4B-003	ENSG00000182217	HIST2H4B	2.72	0.59
ENST00000369155	HIST2H2BE-001	ENSG00000184678	HIST2H2BE	2.82	0.54
ENST00000331380	HIST2H2AC-001	ENSG00000184260	HIST2H2AC	3.11	0.74
ENST00000369146	SV2A-001	ENSG00000159164	SV2A	2.98	2.08
ENST00000369026	MCL1-001	ENSG00000143384	MCL1	2.49	0.93
ENST00000439749	MCL1-201	ENSG00000143384	MCL1	2.19	1.14
ENST00000368947	ANXA9-001	ENSG00000143412	ANXA9	-2.94	1.89
ENST00000368732	S100A8-002	ENSG00000143546	S100A8	2.53	3.71
ENST00000368714	S100A4-006	ENSG00000196154	S100A4	-2.23	0.74
ENST00000368476	CHRNB2-001	ENSG00000160716	CHRNB2	2.43	0.45
ENST00000368471	ADAR-002	ENSG00000160710	ADAR	2.40	0.70
ENST00000355014	SEMA4A-002	ENSG00000196189	SEMA4A	3.33	0.76
ENST00000470306	SEMA4A-005	ENSG00000196189	SEMA4A	2.88	3.20
ENST00000361170	IQGAP3-001	ENSG00000183856	IQGAP3	-2.80	0.68
ENST00000413909	ARHGEF11-202	ENSG00000132694	ARHGEF11	2.87	3.80
ENST00000368174	CD5L-001	ENSG00000073754	CD5L	3.08	0.82
ENST00000368131	IFI16-001	ENSG00000163565	IFI16	2.81	4.23
ENST00000295809	IFI16-201	ENSG00000163565	IFI16	2.56	0.59
ENST00000443928	RP11-404F10-001	ENSG00000228863	RP11-404F10.1	2.59	1.98
ENST00000499766	AL121985-201	ENSG00000247153	AL121985.1	2.76	1.97
ENST00000368046	CD48-001	ENSG00000117091	CD48	2.18	0.33
ENST00000368042	SLAMF7-003	ENSG00000026751	SLAMF7	3.03	6.19
ENST00000484221	SLAMF7-004	ENSG00000026751	SLAMF7	4.04	5.72
ENST00000367838	GPR161-002	ENSG00000143147	GPR161	2.17	3.12
ENST00000329281	BLZF1-002	ENSG00000117475	BLZF1	2.89	0.56
ENST00000367808	BLZF1-001	ENSG00000117475	BLZF1	3.24	0.79
ENST00000236147	SELL-001	ENSG00000188404	SELL	3.50	0.56
ENST00000239468	TNFSF18-201	ENSG00000120337	TNFSF18	5.25	1.51
ENST00000489615	RABGAP1L-006	ENSG00000152061	RABGAP1L	2.71	4.38
ENST00000478442	RABGAP1L-010	ENSG00000152061	RABGAP1L	4.76	2.68
ENST00000437695	RP5-1141O19-001	ENSG00000227085	RP5-1141O19.1	3.60	0.66
ENST00000367511	FAM129A-001	ENSG00000135842	FAM129A	3.41	4.20
ENST00000500715	AL356273-201	ENSG00000247183	AL356273.4	-3.40	3.19
ENST00000367468	PTGS2-001	ENSG00000073756	PTGS2	6.44	1.63
ENST00000367436	CDC73-201	ENSG00000134371	CDC73	2.39	3.86
ENST00000309502	ADORA1-002	ENSG00000163485	ADORA1	-3.57	1.60
ENST00000391949	PIK3C2B-201	ENSG00000133056	PIK3C2B	-2.47	3.39
ENST00000367128	FAM72A-001	ENSG00000196550	FAM72A	3.08	2.00

ENST00000295713	SRGAP2-001	ENSG00000163486	SRGAP2	2.43	3.15
ENST00000419187	SRGAP2-204	ENSG00000163486	SRGAP2	2.73	0.88
ENST00000467419	SRGAP2-003	ENSG00000163486	SRGAP2	2.80	2.04
ENST00000488049	SRGAP2-006	ENSG00000163486	SRGAP2	2.21	0.49
ENST00000501119	AL035209-201	ENSG00000245296	AL035209.1	2.19	0.44
ENST00000367029	G0S2-001	ENSG00000123689	G0S2	2.77	0.82
ENST00000367021	IRF6-001	ENSG00000117595	IRF6	2.13	1.62
ENST00000446962	RP11-338C15-001	ENSG00000237980	RP11-338C15.1	2.19	1.38
ENST00000243440	BATF3-001	ENSG00000123685	BATF3	3.48	1.31
ENST00000478275	BATF3-002	ENSG00000123685	BATF3	4.51	3.01
ENST00000490299	RPS6KC1-003	ENSG00000136643	RPS6KC1	2.27	2.31
ENST00000407981	MOSC1-002	ENSG00000186205	MOSC1	-2.84	1.87
ENST00000427693	HLX-002	ENSG00000136630	HLX	2.37	5.04
ENST00000344922	MIA3-001	ENSG00000154305	MIA3	2.50	3.73
ENST00000340535	MIA3-005	ENSG00000154305	MIA3	2.45	1.49
ENST00000456298	FAM177B-005	ENSG00000197520	FAM177B	3.57	0.66
ENST00000327449	DUSP5P-201	ENSG00000183929	DUSP5P	2.85	0.92
ENST00000493897	ASB13-002	ENSG00000196372	ASB13	-2.36	3.63
ENST00000397250	IL15RA-203	ENSG00000134470	IL15RA	6.70	1.61
ENST00000379775	PFKFB3-002	ENSG00000170525	PFKFB3	4.33	0.81
ENST00000459607	Y_RNA	ENSG00000238366	Y_RNA	2.26	1.62
ENST00000277575	USP6NL-201	ENSG00000148429	USP6NL	3.50	3.42
ENST00000378757	OPTN-007	ENSG00000123240	OPTN	2.30	5.66
ENST00000424614	OPTN-001	ENSG00000123240	OPTN	4.04	2.97
ENST00000442900	RP11-730A19-001	ENSG00000228330	RP11-730A19.6	2.36	1.08
ENST00000426373	RP11-108B14-001	ENSG00000227462	RP11-108B14.3	2.65	0.75
ENST00000376495	OTUD1-001	ENSG00000165312	OTUD1	3.42	1.00
ENST00000477034	MASTL-002	ENSG00000120539	MASTL	3.59	2.61
ENST00000375321	MAP3K8-003	ENSG00000107968	MAP3K8	2.62	4.00
ENST00000473940	CREM-003	ENSG00000095794	CREM	3.39	2.66
ENST00000440465	NAMPTL-001	ENSG00000229644	NAMPTL	3.13	0.91
ENST00000355710	RET-001	ENSG00000165731	RET	2.88	2.04
ENST00000374429	CXCL12-002	ENSG00000107562	CXCL12	4.18	1.19
ENST00000343575	CXCL12-001	ENSG00000107562	CXCL12	3.40	1.15
ENST00000298295	C10orf10-001	ENSG00000165507	C10orf10	2.31	5.26
ENST00000489171	RASSF4-002	ENSG00000107551	RASSF4	2.55	0.36
ENST00000430188	RP11-38L15-001	ENSG00000231187	RP11-38L15.3	2.06	0.58
ENST00000422399	CTSLL4-201	ENSG00000230011	CTSLL4	2.42	1.13
ENST00000374007	ASAH2B-002	ENSG00000204147	ASAH2B	3.86	0.70
ENST00000373290	TSPAN15-001	ENSG00000099282	TSPAN15	-2.73	1.73
ENST00000476605	CBARA1-003	ENSG00000107745	CBARA1	2.71	2.65
ENST00000456375	RP11-165M8-003	ENSG00000223820	RP11-165M8.1	2.35	1.97
ENST00000355740	FAS-005	ENSG00000026103	FAS	3.22	3.25
ENST00000371795	IFIT5-001	ENSG00000152778	IFIT5	3.92	0.32
ENST00000416601	IFIT5-201	ENSG00000152778	IFIT5	5.33	4.40
ENST00000463743	MYOF-006	ENSG00000138119	MYOF	2.26	1.40
ENST00000453258	ENTPD1-005	ENSG00000138185	ENTPD1	-2.68	2.46
ENST00000224337	BLNK-001	ENSG00000095585	BLNK	2.66	5.38
ENST00000501877	AL353719-201	ENSG00000247504	AL353719.1	2.58	0.68
ENST00000497217	SLC25A28-002	ENSG00000155287	SLC25A28	2.14	0.54
ENST00000336486	NFKB2-201	ENSG00000077150	NFKB2	2.06	0.98
ENST00000467116	NFKB2-002	ENSG00000077150	NFKB2	2.18	2.96
ENST00000404739	NT5C2-001	ENSG00000076685	NT5C2	2.37	1.21
ENST00000393258	RP11-332O19-201	ENSG00000213277	RP11-332O19.4	2.21	1.94
ENST00000329905	CALHM1-001	ENSG00000185933	CALHM1	-2.72	1.55
ENST00000435434	RP11-127L20-001	ENSG00000231233	RP11-127L20.2	2.69	1.02
ENST00000369583	DUSP-001	ENSG00000138166	DUSP	3.10	0.49
ENST00000442393	CASP7-205	ENSG00000165806	CASP7	2.43	3.92
ENST00000415217	ADAM8-202	ENSG00000151651	ADAM8	2.26	0.53
ENST00000399817	IFITM2-202	ENSG00000185201	IFITM2	3.05	0.50
ENST00000443559	IFITM2-203	ENSG00000185201	IFITM2	4.74	0.90
ENST00000327366	IFITM2-201	ENSG00000185201	IFITM2	3.40	4.42
ENST00000328221	IFITM1-201	ENSG00000185885	IFITM1	8.11	1.29
ENST00000399808	IFITM3-202	ENSG00000142089	IFITM3	5.49	0.87
ENST00000452428	IFITM3-203	ENSG00000142089	IFITM3	5.61	0.68
ENST00000469048	IRF7-004	ENSG00000185507	IRF7	5.23	0.98
ENST00000331588	DUSP8-201	ENSG00000184545	DUSP8	2.29	1.13
ENST00000381395	IGF2-003	ENSG00000167244	IGF2	4.37	1.45
ENST00000331289	ASCL2-001	ENSG00000183734	ASCL2	3.58	0.67
ENST00000501274	AC013791-201	ENSG00000246000	AC013791.1	2.11	0.86
ENST00000471998	OSBPL5-003	ENSG00000021762	OSBPL5	2.29	2.12

ENST00000416879	AC010930-201	ENSG00000225101	AC010930.2	3.49	0.87
ENST00000433961	TRIM5-005	ENSG00000132256	TRIM5	3.58	0.86
ENST00000379965	TRIM22-001	ENSG00000132274	TRIM22	4.34	3.61
ENST00000480395	TRIM22-002	ENSG00000132274	TRIM22	4.30	0.61
ENST00000414897	TRIM22-007	ENSG00000132274	TRIM22	3.31	5.60
ENST00000328194	DENND5A-201	ENSG00000184014	DENND5A	2.66	0.82
ENST00000278175	ADM-201	ENSG00000148926	ADM	3.06	0.38
ENST00000396553	AMPD3-202	ENSG00000133805	AMPD3	3.17	2.85
ENST00000396554	AMPD3-203	ENSG00000133805	AMPD3	2.84	0.87
ENST00000241051	DEPDC7-201	ENSG00000121690	DEPDC7	2.71	1.41
ENST00000298992	ABTB2-201	ENSG00000166016	ABTB2	3.65	0.42
ENST00000457912	SMTNL1-202	ENSG00000214872	SMTNL1	4.18	1.69
ENST00000395113	UBE2L6-203	ENSG00000156587	UBE2L6	2.85	0.18
ENST00000433668	SERPING1-203	ENSG00000149131	SERPING1	4.53	0.62
ENST00000361723	FAM111A-201	ENSG00000166801	FAM111A	2.07	5.88
ENST00000428532	PATL1-202	ENSG00000166889	PATL1	2.38	5.97
ENST00000300146	PATL1-201	ENSG00000166889	PATL1	1.79	0.61
ENST00000435054	PATL1-203	ENSG00000166889	PATL1	2.85	3.36
ENST00000323961	MS4A6A-201	ENSG00000110077	MS4A6A	-2.80	0.80
ENST00000412309	MS4A6A-202	ENSG00000110077	MS4A6A	-2.78	1.01
ENST00000354445	RARRES3-202	ENSG00000133321	RARRES3	3.98	3.17
ENST00000435842	BATF2-202	ENSG00000168062	BATF2	6.07	2.47
ENST00000301887	BATF2-201	ENSG00000168062	BATF2	5.28	1.92
ENST00000279249	CDC42EP2-201	ENSG00000149798	CDC42EP2	3.75	0.85
ENST00000312106	CATSPER1-201	ENSG00000175294	CATSPER1	-2.63	2.05
ENST00000327259	TMEM151A-201	ENSG00000179292	TMEM151A	-2.68	1.28
ENST00000357440	SLC29A2-202	ENSG00000174669	SLC29A2	-2.59	2.92
ENST00000320740	PELI3-201	ENSG00000174516	PELI3	-2.92	3.23
ENST00000309996	SPTBN2-201	ENSG00000173898	SPTBN2	-2.39	0.51
ENST00000312390	TBC1D10C-201	ENSG00000175463	TBC1D10C	-2.67	0.70
ENST00000309099	MRGPRF-201	ENSG00000172935	MRGPRF	-2.75	2.68
ENST00000308946	MYEOV-201	ENSG00000172927	MYEOV	2.64	1.19
ENST00000321297	ATG16L2-201	ENSG00000168010	ATG16L2	-2.49	4.16
ENST00000353172	KCTD14-001	ENSG00000151364	KCTD14	4.57	1.31
ENST00000309186	FZD4-201	ENSG00000174804	FZD4	4.61	0.90
ENST00000500319	AP001528-201	ENSG00000245350	AP001528.2	3.35	1.48
ENST00000227638	PANX1-201	ENSG00000110218	PANX1	2.76	0.65
ENST00000358752	FUT4-201	ENSG00000196371	FUT4	2.99	0.44
ENST00000446230	PIWIL4-202	ENSG00000134627	PIWIL4	3.70	2.37
ENST00000278505	ENDOD1-201	ENSG00000149218	ENDOD1	3.26	0.46
ENST00000331217	MAML2-201	ENSG00000184384	MAML2	1.96	4.31
ENST00000263464	BIRC3-201	ENSG00000023445	BIRC3	3.34	0.54
ENST00000398136	TMEM123-202	ENSG00000152558	TMEM123	2.19	0.96
ENST00000375735	DYNC2H1-202	ENSG00000187240	DYNC2H1	-2.56	1.35
ENST00000260309	CASP1-201	ENSG00000137752	CASP1	2.58	0.92
ENST00000320578	RAB39-201	ENSG00000179331	RAB39	2.98	0.91
ENST00000481955	RP11-361A21-001	ENSG00000224776	RP11-361A21.1	2.78	0.99
ENST00000280350	PIH1D2-201	ENSG00000150773	PIH1D2	3.17	1.92
ENST00000260282	FXYD6-201	ENSG00000137726	FXYD6	3.13	0.46
ENST00000278940	TMPRSS13-201	ENSG00000137747	TMPRSS13	4.26	2.21
ENST00000227752	IL10RA-201	ENSG00000110324	IL10RA	2.54	0.37
ENST00000356289	AMICA1-202	ENSG00000160593	AMICA1	-4.23	0.77
ENST00000499820	AP000783-201	ENSG00000245866	AP000783.1	2.29	0.65
ENST00000322282	GRAMD1B-201	ENSG00000023171	GRAMD1B	2.96	1.82
ENST00000450171	GRAMD1B-202	ENSG00000023171	GRAMD1B	2.42	1.71
ENST00000284292	NRGN-201	ENSG00000154146	NRGN	-2.65	5.59
ENST00000326261	IQSEC3-201	ENSG00000120645	IQSEC3	3.28	2.17
ENST00000261405	VWF-201	ENSG00000110799	VWF	-2.38	0.47
ENST00000406697	C1S-001	ENSG00000182326	C1S	2.60	0.82
ENST00000266542	C1RL-201	ENSG00000139178	C1RL	3.86	1.62
ENST00000382215	AC233309-201	ENSG00000205885	AC233309.1	3.47	1.21
ENST00000229307	NANOG-201	ENSG00000111704	NANOG	2.71	1.72
ENST00000307637	C3AR1-201	ENSG00000171860	C3AR1	2.02	0.56
ENST00000299663	CLEC4E-201	ENSG00000166523	CLEC4E	4.37	0.85
ENST00000501043	AC023796-201	ENSG00000245492	AC023796.2	-3.53	4.06
ENST00000182377	FAR2-201	ENSG00000064763	FAR2	2.44	0.63
ENST00000453713	AC023157-201	ENSG00000223722	AC023157.1	5.37	0.98
ENST00000395360	RAPGEF3-002	ENSG00000079337	RAPGEF3	-2.54	1.13
ENST00000309739	RND1-201	ENSG00000172602	RND1	3.59	1.12
ENST00000451096	RHEBL1-203	ENSG00000167550	RHEBL1	3.74	1.64
ENST00000420065	RHEBL1-202	ENSG00000167550	RHEBL1	2.90	2.87

ENST00000330553	KRT79-201	ENSG00000185640	KRT79	-2.77	0.60
ENST00000301464	IGFBP6-201	ENSG00000167779	IGFBP6	3.97	1.61
ENST00000422257	ITGB7-203	ENSG00000139626	ITGB7	2.63	2.00
ENST00000267082	ITGB7-201	ENSG00000139626	ITGB7	2.77	4.30
ENST00000267015	GPR84-201	ENSG00000139572	GPR84	2.86	0.33
ENST00000228534	IL23A-201	ENSG00000110944	IL23A	2.33	0.55
ENST00000501134	AC025574-201	ENSG00000247899	AC025574.1	3.30	0.89
ENST00000314128	STAT2-201	ENSG00000170581	STAT2	3.15	0.54
ENST00000262031	RBMS2-201	ENSG00000076067	RBMS2	2.34	0.34
ENST00000300119	MYO1A-001	ENSG00000166866	MYO1A	6.04	1.01
ENST00000379391	TMEM194A-202	ENSG00000166881	TMEM194A	3.77	3.06
ENST00000228606	CYP27B1-201	ENSG00000111012	CYP27B1	3.27	0.57
ENST00000499481	AC083805-201	ENSG00000245651	AC083805.1	5.57	1.87
ENST00000440906	HELB-202	ENSG00000127311	HELB	2.79	3.46
ENST00000500831	AC025423-201	ENSG00000245062	AC025423.2	2.58	2.09
ENST00000258111	KCNMB4-201	ENSG00000135643	KCNMB4	-3.17	1.40
ENST00000438169	KRR1-202	ENSG00000111615	KRR1	2.39	2.65
ENST00000501535	AC025568-201	ENSG00000245720	AC025568.1	-3.02	1.17
ENST00000256015	BTG1-201	ENSG00000133639	BTG1	2.52	0.26
ENST00000393113	AC073655-201	ENSG00000213250	AC073655.1	2.92	0.57
ENST00000258534	DRAM1-201	ENSG00000136048	DRAM1	2.41	0.40
ENST00000337514	IGF1-001	ENSG00000017427	IGF1	-3.37	3.91
ENST00000307046	IGF1-002	ENSG00000017427	IGF1	-3.02	1.54
ENST00000425610	SLC41A2-202	ENSG00000136052	SLC41A2	2.68	0.62
ENST00000397689	AC009729-201	ENSG00000214163	AC009729.1	6.94	0.52
ENST00000478808	USP30-004	ENSG00000135093	USP30	3.82	0.70
ENST00000429628	CUX2-203	ENSG00000111249	CUX2	-3.28	1.08
ENST00000423386	AC137055-201	ENSG00000238168	AC137055.2	2.79	0.62
ENST00000442119	C12orf47-202	ENSG00000234608	C12orf47	-2.51	4.94
ENST00000412615	TRAFD1-202	ENSG00000135148	TRAFD1	2.57	0.24
ENST00000452357	OAS1-205	ENSG00000089127	OAS1	3.74	0.49
ENST00000202917	OAS1-201	ENSG00000089127	OAS1	3.37	0.80
ENST00000228928	OAS3-201	ENSG00000111331	OAS3	5.14	0.51
ENST00000342315	OAS2-201	ENSG00000111335	OAS2	5.92	0.31
ENST00000392583	OAS2-202	ENSG00000111335	OAS2	6.72	0.97
ENST00000449768	OAS2-203	ENSG00000111335	OAS2	4.31	2.89
ENST00000261729	RASAL1-201	ENSG00000111344	RASAL1	-2.95	3.44
ENST00000257549	SDS-201	ENSG00000135094	SDS	2.07	0.41
ENST00000358150	NCRNA00173-201	ENSG00000196668	NCRNA00173	2.67	1.92
ENST00000335209	TESC-201	ENSG00000088992	TESC	-2.80	4.58
ENST00000257570	OASL-001	ENSG00000135114	OASL	9.53	0.90
ENST00000261826	P2RX7-201	ENSG00000089041	P2RX7	2.27	0.97
ENST00000328880	GPR109A-201	ENSG00000182782	GPR109A	5.07	1.38
ENST00000253083	HIP1R-201	ENSG00000130787	HIP1R	3.39	1.02
ENST00000442028	ABCB9-205	ENSG00000150967	ABCB9	-3.34	3.88
ENST00000501992	AC122688-201	ENSG00000245605	AC122688.1	-2.36	0.56
ENST00000281703	GLT1D1-201	ENSG00000151948	GLT1D1	2.42	3.39
ENST00000261654	GPR133-201	ENSG00000111452	GPR133	-2.38	2.37
ENST00000382848	GJB2-001	ENSG00000165474	GJB2	3.36	1.01
ENST00000409126	SPATA13-011	ENSG00000182957	SPATA13	2.21	3.37
ENST00000399762	RNF6-202	ENSG00000127870	RNF6	2.64	5.57
ENST00000241463	RASL11A-001	ENSG00000122035	RASL11A	-2.48	1.85
ENST00000480803	RASL11A-003	ENSG00000122035	RASL11A	-2.63	1.82
ENST00000475385	SLC46A3-002	ENSG00000139508	SLC46A3	-2.96	3.22
ENST00000380152	BRCA2-001	ENSG00000139618	BRCA2	2.09	0.65
ENST00000470094	BRCA2-002	ENSG00000139618	BRCA2	3.04	2.75
ENST00000255465	CCNA1-001	ENSG00000133101	CCNA1	4.38	3.15
ENST00000313624	EPSTI1-001	ENSG00000133106	EPSTI1	6.24	0.89
ENST00000313640	EPSTI1-003	ENSG00000133106	EPSTI1	7.16	0.51
ENST00000261489	TSC22D1-002	ENSG00000102804	TSC22D1	2.34	0.69
ENST00000453033	RP11-278A16-001	ENSG00000225131	RP11-278A16.1	2.96	0.52
ENST00000496623	PHF11-012	ENSG00000136147	PHF11	2.24	2.01
ENST00000465045	PHF11-011	ENSG00000136147	PHF11	2.60	5.65
ENST00000488958	PHF11-010	ENSG00000136147	PHF11	2.30	2.75
ENST00000425106	RP11-522F22-001	ENSG00000223717	RP11-522F22.1	2.19	0.81
ENST00000376958	GPR180-001	ENSG00000152749	GPR180	2.76	1.06
ENST00000375887	TNFSF13B-001	ENSG00000102524	TNFSF13B	4.92	0.52
ENST00000493765	TNFSF13B-004	ENSG00000102524	TNFSF13B	2.88	5.56
ENST00000463102	PCID2-018	ENSG00000126226	PCID2	-2.43	2.71
ENST00000450766	GAS6-204	ENSG00000183087	GAS6	-3.19	2.43
ENST00000327773	GAS6-001	ENSG00000183087	GAS6	-3.10	2.71

ENST00000397532	SLC7A7-002	ENSG00000155465	SLC7A7	2.23	4.45
ENST00000311852	MMP14-001	ENSG00000157227	MMP14	2.03	0.38
ENST00000470718	PSME1-004	ENSG00000092010	PSME1	2.24	0.59
ENST00000396881	PSME2-201	ENSG00000100911	PSME2	2.62	0.49
ENST00000471700	PSME2-003	ENSG00000100911	PSME2	2.97	0.73
ENST00000396864	IRF9-001	ENSG00000213928	IRF9	2.14	0.17
ENST00000324076	IRF9-002	ENSG00000213928	IRF9	2.93	3.35
ENST00000447460	REC8-202	ENSG00000100918	REC8	2.22	2.12
ENST00000258807	CIDEB-201	ENSG00000136305	CIDEB	-3.36	1.54
ENST00000267406	CBLN3-201	ENSG00000139899	CBLN3	2.48	0.84
ENST00000280979	AKAP6-001	ENSG00000151320	AKAP6	-2.97	1.62
ENST00000358716	BAZ1A-201	ENSG00000198604	BAZ1A	2.33	7.30
ENST00000216797	NFKBIA-001	ENSG00000100906	NFKBIA	2.88	0.59
ENST00000500541	AL117692-201	ENSG00000247881	AL117692.1	2.59	1.49
ENST00000395834	CDKL1-001	ENSG00000100490	CDKL1	3.35	1.61
ENST00000335281	GNG2-201	ENSG00000186469	GNG2	-3.99	1.16
ENST00000245457	PTGER2-001	ENSG00000125384	PTGER2	3.49	0.54
ENST00000305831	SAMD4A-202	ENSG00000020577	SAMD4A	2.20	3.38
ENST00000254299	GCH1-001	ENSG00000131979	GCH1	6.63	2.25
ENST00000394709	HSPA2-001	ENSG00000126803	HSPA2	2.36	1.77
ENST00000394691	PLEKHG3-201	ENSG00000126822	PLEKHG3	3.68	2.49
ENST00000471182	PLEKHG3-002	ENSG00000126822	PLEKHG3	3.46	4.30
ENST00000484731	PLEKHG3-007	ENSG00000126822	PLEKHG3	3.03	3.43
ENST00000389722	SPTB-205	ENSG00000070182	SPTB	4.41	3.85
ENST00000357461	TMEM229B-201	ENSG00000198133	TMEM229B	5.34	0.51
ENST00000334696	ENTPD5-201	ENSG00000187097	ENTPD5	2.20	0.84
ENST00000216484	SPTLC2-201	ENSG00000100596	SPTLC2	2.29	0.51
ENST00000298902	IFI27-201	ENSG00000165949	IFI27	8.19	1.04
ENST00000392908	SLC25A29-003	ENSG00000197119	SLC25A29	-2.47	0.81
ENST00000358655	WARS-203	ENSG00000140105	WARS	2.56	0.75
ENST00000355338	WARS-202	ENSG00000140105	WARS	3.66	4.88
ENST00000392880	WARS-204	ENSG00000140105	WARS	2.71	0.84
ENST00000501384	AL157871-201	ENSG00000247574	AL157871.2	2.62	1.78
ENST00000380069	C14orf73-201	ENSG00000205436	C14orf73	3.86	1.57
ENST00000428256	CKB-202	ENSG00000166165	CKB	5.72	3.89
ENST00000329797	GPR132-201	ENSG00000183484	GPR132	2.55	2.03
ENST00000356865	ATP10A-201	ENSG00000206190	ATP10A	3.54	0.92
ENST00000356107	TJP1-201	ENSG00000104067	TJP1	3.68	2.49
ENST00000382688	C15orf52-002	ENSG00000188549	C15orf52	-2.70	1.22
ENST00000338264	TRIM69-001	ENSG00000185880	TRIM69	4.06	1.84
ENST00000396650	C15orf48-201	ENSG00000166920	C15orf48	3.77	0.63
ENST00000344300	C15orf48-001	ENSG00000166920	C15orf48	4.31	0.66
ENST00000390185	hsa-mir-147b	ENSG00000211519	hsa-mir-147b	2.84	4.37
ENST00000261867	SLC30A4-001	ENSG00000104154	SLC30A4	5.84	0.78
ENST00000409454	C15orf21-001	ENSG00000179362	C15orf21	5.18	0.65
ENST00000490076	CYP19A1-005	ENSG00000137869	CYP19A1	2.78	1.76
ENST00000420301	CYP19A1-203	ENSG00000137869	CYP19A1	3.83	1.35
ENST00000499613	AC066613-201	ENSG00000247729	AC066613.1	2.34	1.99
ENST00000454181	LYSMD2-201	ENSG00000140280	LYSMD2	4.04	1.04
ENST00000499648	AC009712-201	ENSG00000247829	AC009712.2	4.28	0.93
ENST00000395135	PML-202	ENSG00000140464	PML	3.88	0.80
ENST00000435786	PML-205	ENSG00000140464	PML	3.77	0.29
ENST00000354026	PML-004	ENSG00000140464	PML	3.29	0.79
ENST00000268058	PML-001	ENSG00000140464	PML	3.66	0.37
ENST00000305428	KIAA1024-201	ENSG00000169330	KIAA1024	2.38	2.27
ENST00000500393	AC015871-201	ENSG00000246404	AC015871.3	2.20	2.17
ENST00000394652	IL16-002	ENSG00000172349	IL16	-2.32	0.41
ENST00000379224	ISG20-201	ENSG00000172183	ISG20	10.41	1.17
ENST00000464728	CHTF18-003	ENSG00000127586	CHTF18	-2.35	2.98
ENST00000293981	FLYWCH2-002	ENSG00000162076	FLYWCH2	-2.69	3.95
ENST00000008180	IL32-201	ENSG00000008517	IL32	3.62	4.69
ENST00000396870	ZNF200-001	ENSG00000010539	ZNF200	2.78	3.78
ENST00000219596	MEFV-001	ENSG00000103313	MEFV	3.40	2.14
ENST00000339854	MEFV-201	ENSG00000103313	MEFV	2.83	3.52
ENST00000268251	ABAT-001	ENSG00000183044	ABAT	-2.77	2.71
ENST00000332029	SOCS1-001	ENSG00000185338	SOCS1	5.94	0.68
ENST00000499115	AC009121-201	ENSG00000245798	AC009121.6	3.77	2.45
ENST00000389126	MKL2-204	ENSG00000186260	MKL2	-2.54	2.67
ENST00000468219	CLEC19A-204	ENSG00000188477	CLEC19A	4.04	0.61
ENST00000341901	SBK1-201	ENSG00000188322	SBK1	3.01	1.86
ENST00000356897	IL27-001	ENSG00000197272	IL27	5.78	1.03

ENST00000324873	NUPR1-001	ENSG00000176046	NUPR1	4.21	0.93
ENST00000416168	AC009133-201	ENSG00000238045	AC009133.1	2.60	0.71
ENST00000358758	PRRT2-001	ENSG00000167371	PRRT2	3.63	3.78
ENST00000500263	AC009133-201	ENSG00000246900	AC009133.2	2.43	0.52
ENST00000219150	CORO1A-001	ENSG00000102879	CORO1A	-2.73	0.59
ENST00000254109	C16orf67-001	ENSG00000131797	C16orf67	3.13	1.95
ENST00000398685	AC142381-201	ENSG00000214627	AC142381.3	3.00	1.01
ENST00000498914	AC007608-201	ENSG00000245295	AC007608.1	2.57	1.52
ENST00000300589	NOD2-001	ENSG00000167207	NOD2	3.05	0.95
ENST00000431240	NOD2-201	ENSG00000167207	NOD2	3.58	1.41
ENST00000379780	CETP-201	ENSG00000087237	CETP	2.76	1.88
ENST00000327982	NLRC5-202	ENSG00000140853	NLRC5	2.59	2.28
ENST00000262510	NLRC5-001	ENSG00000140853	NLRC5	2.47	0.69
ENST00000322384	DPEP2-202	ENSG00000167261	DPEP2	-2.51	0.79
ENST00000393350	MAF-002	ENSG00000178573	MAF	-2.68	0.80
ENST00000393255	AC025280-201	ENSG00000213275	AC025280.1	5.10	0.83
ENST00000332281	SNAI3-201	ENSG00000185669	SNAI3	-2.67	0.76
ENST00000499959	AC092385-201	ENSG00000246879	AC092385.1	-2.70	1.14
ENST00000450523	SERPINF2-202	ENSG00000167711	SERPINF2	-2.89	2.54
ENST00000225538	P2RX1-201	ENSG00000108405	P2RX1	-2.57	0.35
ENST00000397041	ATP2A3-206	ENSG00000074370	ATP2A3	-2.59	4.43
ENST00000315768	C17orf87-201	ENSG00000161929	C17orf87	3.73	0.85
ENST00000399600	C17orf87-001	ENSG00000161929	C17orf87	3.86	0.66
ENST00000307229	NLRP1-001	ENSG00000170233	NLRP1	-3.81	1.61
ENST00000502200	AC055839-201	ENSG00000246184	AC055839.2	-3.32	1.45
ENST00000431790	XAF1-204	ENSG00000132530	XAF1	5.61	0.64
ENST00000173229	NTN1-001	ENSG00000065320	NTN1	2.50	1.15
ENST00000360954	HS3ST3B1-001	ENSG00000125430	HS3ST3B1	6.62	1.68
ENST00000472570	CENPV-004	ENSG00000166582	CENPV	-3.13	1.76
ENST00000440501	SLC46A1-203	ENSG00000076351	SLC46A1	-3.81	4.66
ENST00000225831	CCL2-001	ENSG00000108691	CCL2	6.22	0.90
ENST00000225844	CCL13-001	ENSG00000181374	CCL13	3.87	1.40
ENST00000299977	SLFN5-201	ENSG00000166750	SLFN5	2.49	1.10
ENST00000452764	SLFN12-201	ENSG00000172123	SLFN12	2.30	0.80
ENST00000460530	SLFN12-006	ENSG00000172123	SLFN12	2.12	2.41
ENST00000361112	SLFN12L-201	ENSG00000205045	SLFN12L	4.33	1.16
ENST00000366113	CCL5-002	ENSG00000161570	CCL5	9.87	1.71
ENST00000225245	CCL3-001	ENSG00000006075	CCL3	4.35	1.14
ENST00000394495	CCL4-202	ENSG00000129277	CCL4	7.35	2.42
ENST00000425833	CCL3L1-206	ENSG00000205021	CCL3L1	4.45	1.22
ENST00000422211	CCL3L1-001	ENSG00000205021	CCL3L1	5.13	1.27
ENST00000431231	ARHGAP23-201	ENSG00000225485	ARHGAP23	3.64	0.73
ENST00000502030	AC006449-201	ENSG00000247905	AC006449.1	3.28	0.73
ENST00000301659	GSDMA-201	ENSG00000167914	GSDMA	-3.43	0.98
ENST00000225474	CSF3-001	ENSG00000108342	CSF3	3.68	2.99
ENST00000269593	IGFBP4-001	ENSG00000141753	IGFBP4	2.80	0.88
ENST00000393931	JUP-008	ENSG00000173801	JUP	4.22	1.14
ENST00000310706	JUP-001	ENSG00000173801	JUP	4.41	2.77
ENST00000393888	CNP-002	ENSG00000173786	CNP	2.52	0.47
ENST00000251642	DHX58-001	ENSG00000108771	DHX58	3.91	0.21
ENST00000438323	IFI35-203	ENSG00000068079	IFI35	3.94	3.57
ENST00000415816	IFI35-202	ENSG00000068079	IFI35	4.20	0.58
ENST00000499030	AC109326-201	ENSG00000225584	AC109326.1	2.46	1.06
ENST00000393547	DBF4B-202	ENSG00000161692	DBF4B	2.78	0.98
ENST00000428638	SH3D20-203	ENSG00000159314	SH3D20	2.29	4.19
ENST00000389608	UBE2Z-201	ENSG00000159202	UBE2Z	2.28	0.30
ENST00000499842	AC009720-201	ENSG00000246640	AC009720.1	-2.78	1.56
ENST00000397861	C17orf67-001	ENSG00000214226	C17orf67	2.61	0.45
ENST00000316881	TRIM25-201	ENSG00000121060	TRIM25	2.35	0.68
ENST00000500524	AC004687-201	ENSG00000245620	AC004687.1	3.10	1.23
ENST00000365207	U1	ENSG00000202077	U1	2.61	1.97
ENST00000259008	BRIP1-201	ENSG00000136492	BRIP1	5.98	1.72
ENST00000357585	SSTR2-202	ENSG00000180616	SSTR2	5.11	1.21
ENST00000328630	CD300E-201	ENSG00000186407	CD300E	4.49	5.08
ENST00000392619	CD300E-001	ENSG00000186407	CD300E	6.88	1.40
ENST00000499557	AC087289-201	ENSG00000245670	AC087289.4	1.82	0.52
ENST00000392496	SPHK1-202	ENSG00000176170	SPHK1	2.24	0.88
ENST00000318430	TMC8-202	ENSG00000167895	TMC8	-3.22	0.52
ENST00000330871	SOCS3-201	ENSG00000184557	SOCS3	3.57	0.84
ENST00000501717	AC061992-201	ENSG00000245152	AC061992.4	3.46	0.96
ENST00000500207	AC061992-201	ENSG00000246030	AC061992.1	2.50	3.09

ENST00000262776	LGALS3BP-201	ENSG00000108679	LGALS3BP	3.09	0.65
ENST00000418895	LGALS3BP-202	ENSG00000108679	LGALS3BP	3.40	0.89
ENST00000319921	AC124319-201	ENSG00000180843	AC124319.2	3.96	0.24
ENST00000456466	AC124319-203	ENSG00000180843	AC124319.2	3.82	0.87
ENST00000411702	AC124319-202	ENSG00000180843	AC124319.2	3.54	0.31
ENST00000336301	RNF213-201	ENSG00000173821	RNF213	4.11	1.13
ENST00000437954	AC124319-201	ENSG00000237199	AC124319.3	3.58	0.55
ENST00000434136	AC124319-201	ENSG00000234591	AC124319.1	3.18	0.50
ENST00000326724	AATK-001	ENSG00000181409	AATK	-2.64	3.63
ENST00000306897	RAC3-201	ENSG00000169750	RAC3	-2.49	0.97
ENST00000312648	CD7-201	ENSG00000173762	CD7	2.28	2.01
ENST00000269389	SECTM1-201	ENSG00000141574	SECTM1	3.71	0.53
ENST00000300784	FN3K-201	ENSG00000167363	FN3K	-2.53	1.06
ENST00000400256	COLEC12-201	ENSG00000158270	COLEC12	-3.39	1.52
ENST00000261597	NDC80-001	ENSG00000080986	NDC80	2.38	0.94
ENST00000256925	CABLES1-201	ENSG00000134508	CABLES1	-3.08	2.54
ENST00000400473	CABLES1-202	ENSG00000134508	CABLES1	-2.42	1.12
ENST00000217740	RNF125-001	ENSG00000101695	RNF125	-3.56	0.75
ENST00000257190	RNF138-002	ENSG00000134758	RNF138	2.69	2.68
ENST00000399218	FAM59A-001	ENSG00000141441	FAM59A	3.07	0.97
ENST00000409746	PSTPIP2-002	ENSG00000152229	PSTPIP2	3.55	2.74
ENST00000460200	PSTPIP2-004	ENSG00000152229	PSTPIP2	3.41	1.17
ENST00000502112	AC090355-201	ENSG00000245457	AC090355.2	2.69	1.50
ENST00000332053	ZBTB7C-201	ENSG00000184828	ZBTB7C	-2.54	1.09
ENST00000316660	PMAIP1-001	ENSG00000141682	PMAIP1	4.67	1.02
ENST00000269518	PMAIP1-002	ENSG00000141682	PMAIP1	5.22	1.79
ENST00000398175	AC090377-201	ENSG00000214349	AC090377.5	2.84	0.78
ENST00000252288	GAMT-201	ENSG00000130005	GAMT	-3.83	2.71
ENST00000404279	LINGO3-201	ENSG00000220008	LINGO3	-2.63	2.02
ENST00000417546	AC104530-201	ENSG00000235353	AC104530.1	-3.21	1.85
ENST00000382159	GNG7-201	ENSG00000176533	GNG7	-3.16	1.65
ENST00000499973	AC011522-201	ENSG00000244897	AC011522.1	-3.98	2.30
ENST00000394321	MCOLN1-202	ENSG00000090674	MCOLN1	-2.43	2.59
ENST00000221515	RETN-201	ENSG00000104918	RETN	-2.76	2.34
ENST00000423345	PRAM1-202	ENSG00000133246	PRAM1	-3.60	2.74
ENST00000253110	C19orf66-201	ENSG00000130813	C19orf66	3.22	0.29
ENST00000264832	ICAM1-201	ENSG00000090339	ICAM1	3.15	0.55
ENST00000393423	RGL3-203	ENSG00000205517	RGL3	-2.93	2.54
ENST00000360105	NFIX-203	ENSG00000008441	NFIX	2.87	2.77
ENST00000357018	CACNA1A-202	ENSG00000141837	CACNA1A	4.74	1.94
ENST00000269720	AC022098-201	ENSG00000141854	AC022098.1	-2.59	1.17
ENST00000397372	HSH2D-202	ENSG00000196684	HSH2D	8.45	1.96
ENST00000252590	PLVAP-201	ENSG00000130300	PLVAP	2.83	1.46
ENST00000252593	BST2-201	ENSG00000130303	BST2	2.24	0.61
ENST00000500836	AC010319-201	ENSG00000244887	AC010319.2	2.57	0.35
ENST00000458235	JAK3-204	ENSG00000105639	JAK3	5.53	0.65
ENST00000222249	KCNN1-201	ENSG00000105642	KCNN1	5.45	1.69
ENST00000392406	LRRC25-202	ENSG00000175489	LRRC25	-2.76	2.67
ENST00000317991	GRAMD1A-201	ENSG00000089351	GRAMD1A	3.86	3.92
ENST00000270310	FXYD7-201	ENSG00000221946	FXYD7	-2.49	1.20
ENST00000246549	FFAR2-201	ENSG00000126262	FFAR2	4.30	1.02
ENST00000007510	SNX26-201	ENSG00000004777	SNX26	-2.56	3.06
ENST00000454404	RASGRP4-204	ENSG00000171777	RASGRP4	-3.34	5.61
ENST00000221347	FCGBP-201	ENSG00000090920	FCGBP	-4.09	0.73
ENST00000428281	FCGBP-202	ENSG00000090920	FCGBP	-4.63	1.54
ENST00000434370	FCGBP-203	ENSG00000090920	FCGBP	-4.73	2.32
ENST00000352632	SPTBN4-203	ENSG00000160460	SPTBN4	-2.74	3.00
ENST00000359092	AXL-202	ENSG00000167601	AXL	4.71	1.27
ENST00000415495	CEACAM3-003	ENSG00000170956	CEACAM3	-2.52	2.09
ENST00000403534	BCL3-201	ENSG00000069399	BCL3	2.16	0.36
ENST00000473468	BCL3-004	ENSG00000069399	BCL3	2.11	4.26
ENST00000270233	BCAM-201	ENSG00000187244	BCAM	-2.71	1.46
ENST00000252483	PVRL2-201	ENSG00000130202	PVRL2	2.15	0.29
ENST00000252485	PVRL2-202	ENSG00000130202	PVRL2	2.09	0.67
ENST00000221452	RELB-201	ENSG00000104856	RELB	2.43	0.60
ENST00000291294	PTGIR-201	ENSG00000160013	PTGIR	2.20	0.98
ENST00000300880	BBC3-201	ENSG00000105327	BBC3	2.50	2.33
ENST00000354276	PLA2G4C-201	ENSG00000105499	PLA2G4C	2.75	0.48
ENST00000200453	PPP1R15A-201	ENSG00000087074	PPP1R15A	2.86	0.67
ENST00000501000	AC026803-201	ENSG00000247915	AC026803.1	3.25	1.77
ENST00000270645	RCN3-201	ENSG00000142552	RCN3	-3.16	0.79

ENST00000246801	TSKS-201	ENSG00000126467	TSKS	-2.71	2.03
ENST00000391833	AKT1S1-005	ENSG00000204673	AKT1S1	2.94	0.68
ENST00000391832	AKT1S1-003	ENSG00000204673	AKT1S1	2.98	3.70
ENST00000391835	AKT1S1-002	ENSG00000204673	AKT1S1	4.63	0.73
ENST00000391831	AKT1S1-004	ENSG00000204673	AKT1S1	3.04	0.99
ENST00000221543	TBC1D17-201	ENSG00000104946	TBC1D17	2.11	0.86
ENST00000391826	IL4I1-203	ENSG00000104951	IL4I1	4.80	0.43
ENST00000423777	ATF5-202	ENSG00000169136	ATF5	3.59	0.85
ENST00000306139	ATF5-201	ENSG00000169136	ATF5	3.80	0.81
ENST00000253720	NAPSB-201	ENSG00000131401	NAPSB	3.11	1.10
ENST00000391813	SHANK1-003	ENSG00000161681	SHANK1	-3.26	2.63
ENST00000500289	AC011473-201	ENSG00000245686	AC011473.1	-3.14	1.73
ENST00000340023	FPR2-201	ENSG00000171049	FPR2	3.41	1.08
ENST00000339223	FPR3-201	ENSG00000187474	FPR3	2.28	0.75
ENST00000396331	LILRB1-002	ENSG00000104972	LILRB1	4.65	0.65
ENST00000430952	LILRB4-002	ENSG00000186818	LILRB4	1.60	0.44
ENST00000434286	LILRB4-006	ENSG00000186818	LILRB4	2.43	0.45
ENST00000502093	AC017076-201	ENSG00000247107	AC017076.1	7.85	1.01
ENST00000382040	RSAD2-001	ENSG00000134321	RSAD2	10.13	1.93
ENST00000304567	RRM2-201	ENSG00000171848	RRM2	-2.56	1.18
ENST00000295082	KCNF1-001	ENSG00000162975	KCNF1	-3.69	1.52
ENST00000396123	GREB1-209	ENSG00000196208	GREB1	-2.82	0.45
ENST00000405331	TRIB2-004	ENSG00000071575	TRIB2	3.55	2.05
ENST00000448919	C2orf84-009	ENSG00000219626	C2orf84	-2.55	2.14
ENST00000490823	KHK-003	ENSG00000138030	KHK	-2.93	1.56
ENST00000429697	KHK-006	ENSG00000138030	KHK	-2.35	1.80
ENST00000444257	PLB1-005	ENSG00000163803	PLB1	-3.01	1.98
ENST00000465300	FAM179A-001	ENSG00000189350	FAM179A	-3.66	1.09
ENST00000331664	C2orf71-001	ENSG00000179270	C2orf71	-3.50	1.13
ENST00000233057	EIF2AK2-001	ENSG00000055332	EIF2AK2	3.32	0.41
ENST00000395127	EIF2AK2-203	ENSG00000055332	EIF2AK2	3.63	4.41
ENST00000272249	HNRPLL-005	ENSG00000143889	HNRPLL	2.25	2.71
ENST00000410063	GALM-003	ENSG00000143891	GALM	2.55	4.74
ENST00000467135	PRKCE-002	ENSG00000171132	PRKCE	2.04	2.80
ENST00000450096	PNPT1-201	ENSG00000138035	PNPT1	2.86	2.34
ENST00000481066	PNPT1-005	ENSG00000138035	PNPT1	4.17	0.87
ENST00000496857	EHBP1-009	ENSG00000115504	EHBP1	2.21	2.17
ENST00000358912	PELI1-001	ENSG00000197329	PELI1	2.95	1.06
ENST00000481714	CNRIP1-003	ENSG00000119865	CNRIP1	-2.25	1.06
ENST00000409202	ARHGAP25-001	ENSG00000163219	ARHGAP25	2.06	5.76
ENST00000264444	MXD1-001	ENSG00000059728	MXD1	2.56	0.60
ENST00000454815	TIA1-013	ENSG00000116001	TIA1	2.71	3.42
ENST00000258083	C2orf7-001	ENSG00000135617	C2orf7	-2.36	0.54
ENST00000428767	ALMS1P-001	ENSG00000163016	ALMS1P	2.75	1.64
ENST00000470592	MTHFD2-007	ENSG00000065911	MTHFD2	2.50	4.57
ENST00000306384	VAMP5-001	ENSG00000168899	VAMP5	4.60	1.20
ENST00000455121	AC105053-002	ENSG00000232504	AC105053.3	2.51	2.32
ENST00000458519	AC008268-005	ENSG00000237510	AC008268.4	-2.70	2.29
ENST00000482704	GPAT2-210	ENSG00000186281	GPAT2	-2.66	1.35
ENST00000409345	ADRA2B-001	ENSG00000222040	ADRA2B	-3.56	0.69
ENST00000240423	NCAPH-001	ENSG00000121152	NCAPH	-2.82	2.95
ENST00000427946	NCAPH-002	ENSG00000121152	NCAPH	-2.49	3.15
ENST00000435349	NCAPH-006	ENSG00000121152	NCAPH	-2.66	0.50
ENST00000454558	ARID5A-004	ENSG00000196843	ARID5A	2.44	0.64
ENST00000357682	BCL2L11-202	ENSG00000153094	BCL2L11	2.29	0.93
ENST00000354115	IL1RN-002	ENSG00000136689	IL1RN	3.17	0.72
ENST00000409930	IL1RN-005	ENSG00000136689	IL1RN	4.18	4.31
ENST00000376448	CBWD2-201	ENSG00000136682	CBWD2	2.30	1.58
ENST00000306406	TMEM37-001	ENSG00000171227	TMEM37	-2.89	0.67
ENST00000452780	EPB41L5-007	ENSG00000115109	EPB41L5	2.16	2.99
ENST00000438005	AC012363-001	ENSG00000223549	AC012363.5	2.47	1.30
ENST00000445926	AC012363-001	ENSG00000236878	AC012363.11	2.25	0.99
ENST00000415971	AC012363-001	ENSG00000234065	AC012363.6	2.49	1.27
ENST00000428909	AC013444-001	ENSG00000229781	AC013444.2	2.41	1.30
ENST00000331426	RBM43-001	ENSG00000184898	RBM43	2.56	0.58
ENST00000243346	NMI-001	ENSG00000123609	NMI	3.15	0.37
ENST00000421360	AC092584-001	ENSG00000236917	AC092584.1	2.33	1.19
ENST00000339562	NR4A2-001	ENSG00000153234	NR4A2	3.28	1.79
ENST00000259075	TANK-001	ENSG00000136560	TANK	2.47	3.51
ENST00000360534	DPP4-001	ENSG00000197635	DPP4	5.54	2.51
ENST00000491591	DPP4-010	ENSG00000197635	DPP4	4.52	1.77

ENST00000502006	AC008063-201	ENSG00000246092	AC008063.2	3.60	0.66
ENST00000432251	AC008063-001	ENSG00000233397	AC008063.3	2.80	1.58
ENST00000263642	IFIH1-001	ENSG00000115267	IFIH1	6.21	0.57
ENST00000473240	GCA-013	ENSG00000115271	GCA	4.34	2.36
ENST00000409882	GALNT3-010	ENSG00000115339	GALNT3	3.13	0.97
ENST00000190611	OSBPL6-001	ENSG00000079156	OSBPL6	2.32	2.00
ENST00000361099	STAT1-001	ENSG00000115415	STAT1	4.76	0.85
ENST00000392322	STAT1-003	ENSG00000115415	STAT1	3.68	6.94
ENST00000320717	GLS-001	ENSG00000115419	GLS	2.31	0.57
ENST00000447143	STAT4-201	ENSG00000138378	STAT4	4.20	3.21
ENST00000409140	SPATS2L-005	ENSG00000196141	SPATS2L	2.33	2.43
ENST00000341582	CFLAR-006	ENSG00000003402	CFLAR	2.07	0.35
ENST00000395148	CFLAR-005	ENSG00000003402	CFLAR	2.94	0.31
ENST00000423241	CFLAR-008	ENSG00000003402	CFLAR	2.41	3.95
ENST00000440180	CFLAR-010	ENSG00000003402	CFLAR	2.70	1.96
ENST00000457277	CFLAR-011	ENSG00000003402	CFLAR	2.50	2.88
ENST00000462763	CFLAR-023	ENSG00000003402	CFLAR	2.90	3.39
ENST00000460961	CFLAR-024	ENSG00000003402	CFLAR	2.60	0.41
ENST00000415011	ALS2CR10-001	ENSG00000226312	ALS2CR10	2.47	0.68
ENST00000286186	CASP10-001	ENSG00000003400	CASP10	2.76	3.83
ENST00000432109	CASP8-014	ENSG00000064012	CASP8	2.91	3.22
ENST00000490682	CASP8-008	ENSG00000064012	CASP8	3.11	0.52
ENST00000445231	BMPR2-201	ENSG00000204217	BMPR2	2.26	0.56
ENST00000408943	PLEKHM3-203	ENSG00000178385	PLEKHM3	2.34	3.75
ENST00000392194	MAP2-002	ENSG00000078018	MAP2	4.95	2.15
ENST00000260947	BARD1-001	ENSG00000138376	BARD1	2.12	0.73
ENST00000441828	RUFY4-202	ENSG00000188282	RUFY4	5.29	1.69
ENST00000439871	IL8RBP-002	ENSG00000229754	IL8RBP	2.89	0.73
ENST00000490275	GPBAR1-002	ENSG00000179921	GPBAR1	3.26	5.04
ENST00000354867	GPBAR1-201	ENSG00000179921	GPBAR1	4.22	2.45
ENST00000441749	AC021016-001	ENSG00000225062	AC021016.3	-2.79	1.13
ENST00000233202	SLC11A1-001	ENSG00000018280	SLC11A1	-2.52	0.96
ENST00000494322	SLC11A1-010	ENSG00000018280	SLC11A1	-3.58	3.43
ENST00000490872	SLC11A1-019	ENSG00000018280	SLC11A1	-2.69	3.30
ENST00000490536	SLC11A1-012	ENSG00000018280	SLC11A1	-2.64	2.44
ENST00000392068	SGPP2-201	ENSG00000163082	SGPP2	4.18	1.77
ENST00000343805	SP140-009	ENSG00000079263	SP140	4.52	4.76
ENST00000373645	SP140-003	ENSG00000079263	SP140	3.78	0.88
ENST00000483728	SP140L-002	ENSG00000185404	SP140L	2.68	2.93
ENST00000483776	SP140L-008	ENSG00000185404	SP140L	2.92	2.85
ENST00000477068	SP110-005	ENSG00000135899	SP110	3.79	0.32
ENST00000392048	SP110-004	ENSG00000135899	SP110	3.86	0.24
ENST00000489597	SP110-012	ENSG00000135899	SP110	3.84	2.77
ENST00000409341	SP100-006	ENSG00000067066	SP100	2.86	4.05
ENST00000494901	SP100-007	ENSG00000067066	SP100	2.52	2.76
ENST00000410320	SRP_euk_arch	ENSG00000222252	SRP_euk_arch	2.48	1.78
ENST00000305141	NMUR1-001	ENSG00000171596	NMUR1	-3.64	1.45
ENST00000442811	AC104809-002	ENSG00000226321	AC104809.2	-4.93	1.25
ENST00000418218	AC104809-002	ENSG00000233392	AC104809.3	-5.89	1.32
ENST00000438506	AC104809-003	ENSG00000233392	AC104809.3	-4.74	0.85
ENST00000334409	PDCD1-001	ENSG00000188389	PDCD1	3.47	1.88
ENST00000380605	CPXM1-001	ENSG00000088882	CPXM1	2.76	2.19
ENST00000419548	SIGLEC1-002	ENSG00000088827	SIGLEC1	3.07	0.93
ENST00000344754	SIGLEC1-001	ENSG00000088827	SIGLEC1	2.76	0.76
ENST00000378827	BMP2-001	ENSG00000125845	BMP2	2.81	1.76
ENST00000484638	RIN2-001	ENSG00000132669	RIN2	3.01	1.25
ENST00000377103	THBD-001	ENSG00000178726	THBD	-4.69	1.03
ENST00000375862	HCK-002	ENSG00000101336	HCK	2.36	4.84
ENST00000501883	AL121906-201	ENSG00000246833	AL121906.1	-3.29	1.59
ENST00000397711	MAP1LC3A-201	ENSG00000101460	MAP1LC3A	5.03	3.16
ENST00000216968	PROCR-201	ENSG00000101000	PROCR	3.34	2.96
ENST00000438192	PPP1R16B-004	ENSG00000101445	PPP1R16B	2.12	2.33
ENST00000372285	CD40-001	ENSG00000101017	CD40	3.10	0.69
ENST00000372278	CD40-201	ENSG00000101017	CD40	3.41	0.35
ENST00000466205	CD40-006	ENSG00000101017	CD40	4.48	3.04
ENST00000371752	ZNFX1-001	ENSG00000124201	ZNFX1	2.96	0.40
ENST00000498985	AL035683-201	ENSG00000247323	AL035683.1	3.00	2.24
ENST00000449816	RNF114-202	ENSG00000124226	RNF114	2.98	3.00
ENST00000244050	SNAI1-001	ENSG00000124216	SNAI1	4.01	1.32
ENST00000252889	RP4-697K14-201	ENSG00000130589	RP4-697K14.2	5.90	2.36
ENST00000467148	RP4-697K14-003	ENSG00000130589	RP4-697K14.2	5.48	6.27

ENST00000479540	RP4-697K14-005	ENSG00000130589	RP4-697K14.2	5.87	0.62
ENST00000389440	SAMSN1-201	ENSG00000155307	SAMSN1	2.63	3.04
ENST00000435315	AF127936-001	ENSG00000226751	AF127936.4	3.22	1.86
ENST00000284881	C21orf91-003	ENSG00000154642	C21orf91	2.95	1.83
ENST00000501838	AL109616-201	ENSG00000246147	AL109616.1	3.82	0.94
ENST00000456917	MIRHG2-001	ENSG00000234883	MIRHG2	4.03	0.80
ENST00000502007	C21orf71-201	ENSG00000247211	C21orf71	4.32	1.88
ENST00000333063	OLIG1-201	ENSG00000184221	OLIG1	2.41	2.72
ENST00000290349	CBR1-001	ENSG00000159228	CBR1	2.44	0.19
ENST00000472398	TTC3-006	ENSG00000182670	TTC3	-3.10	3.12
ENST00000330714	MX2-001	ENSG00000183486	MX2	6.42	0.66
ENST00000482953	MX2-002	ENSG00000183486	MX2	5.84	0.53
ENST00000474368	MX2-004	ENSG00000183486	MX2	5.46	0.74
ENST00000398598	MX1-002	ENSG00000157601	MX1	9.36	3.10
ENST00000455164	MX1-201	ENSG00000157601	MX1	7.47	3.89
ENST00000400377	ICOSLG-002	ENSG00000160223	ICOSLG	2.49	3.92
ENST00000473212	COL18A1-004	ENSG00000182871	COL18A1	-2.77	1.20
ENST00000468508	SLC19A1-011	ENSG00000173638	SLC19A1	-3.63	1.66
ENST00000311124	SLC19A1-001	ENSG00000173638	SLC19A1	-3.31	0.86
ENST00000474319	LSS-003	ENSG00000160285	LSS	2.54	0.76
ENST00000397728	LSS-002	ENSG00000160285	LSS	2.27	0.56
ENST00000397679	C21orf58-004	ENSG00000160298	C21orf58	-2.83	1.81
ENST00000291700	S100B-001	ENSG00000160307	S100B	-2.40	1.65
ENST00000399777	BCL2L13-006	ENSG00000099968	BCL2L13	2.27	0.38
ENST00000215794	USP18-001	ENSG00000184979	USP18	6.39	0.78
ENST00000263208	HIRA-001	ENSG00000100084	HIRA	1.67	0.49
ENST00000292729	USP41-001	ENSG00000161133	USP41	7.28	0.95
ENST00000470202	USP41-002	ENSG00000161133	USP41	6.83	1.27
ENST00000382701	ASPHD2-201	ENSG00000128203	ASPHD2	3.17	0.75
ENST00000215939	CRYBB1-001	ENSG00000100122	CRYBB1	-2.97	2.86
ENST00000302326	MN1-001	ENSG00000169184	MN1	4.07	0.96
ENST00000344347	XBP1-201	ENSG00000100219	XBP1	2.15	3.64
ENST00000215781	OSM-001	ENSG00000099985	OSM	3.11	1.48
ENST00000400289	SFI1-014	ENSG00000198089	SFI1	2.31	3.22
ENST00000442204	C22orf28-203	ENSG00000100220	C22orf28	2.86	3.01
ENST00000426906	C22orf28-201	ENSG00000100220	C22orf28	2.58	0.31
ENST00000409652	APOL6-001	ENSG00000221963	APOL6	3.28	0.45
ENST00000479929	APOL4-003	ENSG00000100336	APOL4	2.58	0.85
ENST00000426053	APOL1-203	ENSG00000100342	APOL1	4.45	6.85
ENST00000416338	APOL1-007	ENSG00000100342	APOL1	3.30	6.00
ENST00000397279	APOL1-201	ENSG00000100342	APOL1	2.89	1.08
ENST00000262825	CSF2RB-201	ENSG00000100368	CSF2RB	2.49	3.95
ENST00000215886	LGALS2-001	ENSG00000100079	LGALS2	4.01	1.55
ENST00000216044	GTPBP1-001	ENSG00000100226	GTPBP1	2.64	0.32
ENST00000458073	GTPBP1-010	ENSG00000100226	GTPBP1	3.47	5.41
ENST00000469086	UNC84B-013	ENSG00000100242	UNC84B	2.62	3.95
ENST00000499426	AL008583-201	ENSG00000244850	AL008583.1	3.09	2.21
ENST00000396770	APOBEC3A-202	ENSG00000128383	APOBEC3A	8.44	6.52
ENST00000249116	APOBEC3A-201	ENSG00000128383	APOBEC3A	9.87	1.02
ENST00000308521	APOBEC3F-001	ENSG00000128394	APOBEC3F	4.21	0.40
ENST00000381565	APOBEC3F-002	ENSG00000128394	APOBEC3F	2.54	0.54
ENST00000476513	APOBEC3F-004	ENSG00000128394	APOBEC3F	3.10	0.84
ENST00000263247	APOBEC3G-201	ENSG00000239713	APOBEC3G	4.55	0.39
ENST00000407997	APOBEC3G-001	ENSG00000239713	APOBEC3G	4.90	2.82
ENST00000494150	APOBEC3G-007	ENSG00000239713	APOBEC3G	3.86	0.35
ENST00000481958	APOBEC3G-003	ENSG00000239713	APOBEC3G	3.55	0.52
ENST00000216133	CBX7-001	ENSG00000100307	CBX7	-2.43	0.83
ENST00000441207	RP4-742C19-001	ENSG00000227009	RP4-742C19.2	2.78	0.61
ENST00000441316	RP4-756G23-001	ENSG00000235513	RP4-756G23.1	2.17	0.73
ENST00000463886	PPPDE2-002	ENSG00000100418	PPPDE2	2.24	4.69
ENST00000216180	PNPLA3-001	ENSG00000100344	PNPLA3	-4.58	2.12
ENST00000453888	PARVG-204	ENSG00000138964	PARVG	-3.03	0.48
ENST00000417767	PARVG-010	ENSG00000138964	PARVG	-2.60	3.39
ENST00000501307	AL096766-201	ENSG00000246911	AL096766.1	-2.40	2.24
ENST00000497738	MAPK12-008	ENSG00000188130	MAPK12	-2.53	3.78
ENST00000496227	NCAPH2-010	ENSG00000025770	NCAPH2	2.91	0.79
ENST00000252785	SCO2-002	ENSG00000130489	SCO2	4.02	4.24
ENST00000449555	TYMP-203	ENSG00000025708	TYMP	2.48	0.64
ENST00000487577	TYMP-007	ENSG00000025708	TYMP	2.20	0.70
ENST00000476284	TYMP-002	ENSG00000025708	TYMP	2.54	5.18
ENST00000425169	TYMP-008	ENSG00000025708	TYMP	2.29	3.89

ENST00000419305	TYMP-201	ENSG00000025708	TYMP	2.18	7.01
ENST00000487162	TYMP-004	ENSG00000025708	TYMP	2.60	0.59
ENST00000395676	KLHDC7B-001	ENSG00000130487	KLHDC7B	3.49	2.35
ENST00000423069	CPT1B-012	ENSG00000205560	CPT1B	2.33	3.24
ENST00000476790	CPT1B-010	ENSG00000205560	CPT1B	2.31	3.06
ENST00000450418	AC023480-001	ENSG00000229241	AC023480.1	4.20	1.19
ENST00000500408	AC008151-201	ENSG00000246544	AC008151.1	4.13	2.98
ENST00000482803	CAMK1-004	ENSG00000134072	CAMK1	-2.71	3.52
ENST00000256458	IRAK2-001	ENSG00000134070	IRAK2	3.56	0.91
ENST00000454374	AC090947-203	ENSG00000224182	AC090947.1	-2.87	1.63
ENST00000396999	PPARG-014	ENSG00000132170	PPARG	-3.13	2.35
ENST00000429166	PPARG-016	ENSG00000132170	PPARG	-3.02	2.58
ENST00000285046	FGD5-001	ENSG00000154783	FGD5	-2.58	2.98
ENST00000393779	AC104297-001	ENSG00000213383	AC104297.1	2.32	0.50
ENST00000263754	KAT2B-001	ENSG00000114166	KAT2B	2.35	1.05
ENST00000301807	LBA1-201	ENSG00000168016	LBA1	4.26	0.74
ENST00000429976	LBA1-202	ENSG00000168016	LBA1	3.77	4.03
ENST00000396428	LRRFIP2-006	ENSG00000093167	LRRFIP2	2.25	3.51
ENST00000417037	MYD88-202	ENSG00000172936	MYD88	2.48	1.33
ENST00000484513	MYD88-007	ENSG00000172936	MYD88	2.36	3.29
ENST00000273153	CSRNP1-001	ENSG00000144655	CSRNP1	4.13	0.72
ENST00000499886	AC092053-201	ENSG00000246007	AC092053.1	3.84	0.50
ENST00000354698	ZNF589-001	ENSG00000164048	ZNF589	-2.99	2.21
ENST00000333486	UBA7-001	ENSG00000182179	UBA7	2.15	0.30
ENST00000488536	UBA7-004	ENSG00000182179	UBA7	2.37	0.32
ENST00000460703	UBA7-009	ENSG00000182179	UBA7	1.96	0.45
ENST00000475688	ZMYND10-005	ENSG00000004838	ZMYND10	-2.51	1.64
ENST00000495880	DUSP7-001	ENSG00000164086	DUSP7	-2.35	2.57
ENST00000469623	DUSP7-002	ENSG00000164086	DUSP7	-2.44	1.78
ENST00000441742	AC115284-201	ENSG00000233341	AC115284.1	-2.52	0.46
ENST00000231721	SEMA3G-001	ENSG00000010319	SEMA3G	-3.29	0.86
ENST00000321725	STAB1-001	ENSG00000010327	STAB1	-2.87	0.72
ENST00000468472	MUSTN1-004	ENSG00000243696	MUSTN1	3.28	0.37
ENST00000495552	MUSTN1-003	ENSG00000243696	MUSTN1	3.06	0.50
ENST00000355083	TMEM110-004	ENSG00000213533	TMEM110	3.29	0.34
ENST00000464769	TMEM110-005	ENSG00000213533	TMEM110	3.06	0.67
ENST00000478366	RP5-966M1-001	ENSG00000239799	RP5-966M1.1	3.00	1.43
ENST00000500808	AC097015-201	ENSG00000247777	AC097015.3	2.07	1.03
ENST00000473921	HESX1-002	ENSG00000163666	HESX1	5.90	1.11
ENST00000481527	ACOX2-013	ENSG00000168306	ACOX2	-2.75	2.55
ENST00000431462	AC108724-201	ENSG00000234317	AC108724.2	3.17	0.72
ENST00000463183	RP11-413E6-001	ENSG00000242516	RP11-413E6.5	2.28	1.84
ENST00000486971	C3orf38-003	ENSG00000179021	C3orf38	2.80	3.60
ENST00000490574	TFG-003	ENSG00000114354	TFG	2.58	5.35
ENST00000361309	CD47-004	ENSG00000196776	CD47	2.40	0.69
ENST00000478182	CD80-002	ENSG00000121594	CD80	4.73	0.51
ENST00000463729	CD80-003	ENSG00000121594	CD80	4.65	3.23
ENST00000478399	ADPRH-001	ENSG00000144843	ADPRH	2.33	3.46
ENST00000500908	AC023494-201	ENSG00000247078	AC023494.1	2.28	1.46
ENST00000295633	FSTL1-001	ENSG00000163430	FSTL1	2.19	1.12
ENST00000492382	PARP9-004	ENSG00000138496	PARP9	4.07	1.69
ENST00000462315	PARP9-003	ENSG00000138496	PARP9	3.71	0.65
ENST00000296161	DTX3L-001	ENSG00000163840	DTX3L	2.90	0.27
ENST00000483793	PARP15-003	ENSG00000173200	PARP15	2.83	0.81
ENST00000464300	PARP15-001	ENSG00000173200	PARP15	2.94	2.42
ENST00000310366	PARP15-002	ENSG00000173200	PARP15	3.03	4.99
ENST00000398157	PARP14-203	ENSG00000173193	PARP14	3.71	5.67
ENST00000499221	AC092983-201	ENSG00000245009	AC092983.1	2.47	2.11
ENST00000319340	CHST13-201	ENSG00000180767	CHST13	-2.70	0.50
ENST00000392981	XRN1-004	ENSG00000114127	XRN1	3.11	0.57
ENST00000463916	XRN1-003	ENSG00000114127	XRN1	3.25	2.60
ENST00000501539	AC023508-201	ENSG00000247996	AC023508.2	2.54	1.01
ENST00000502194	AC023508-201	ENSG00000247567	AC023508.1	3.15	1.05
ENST00000309575	CHST2-001	ENSG00000175040	CHST2	2.40	0.83
ENST00000493432	PLSCR1-005	ENSG00000188313	PLSCR1	4.97	0.81
ENST00000448787	PLSCR1-003	ENSG00000188313	PLSCR1	5.69	3.39
ENST00000483300	PLSCR1-018	ENSG00000188313	PLSCR1	5.80	2.55
ENST00000470496	PLSCR1-020	ENSG00000188313	PLSCR1	5.26	2.63
ENST00000462666	PLSCR1-014	ENSG00000188313	PLSCR1	4.78	5.40
ENST00000325602	P2RY13-001	ENSG00000181631	P2RY13	-2.79	1.57
ENST00000492569	RP11-292E2-001	ENSG00000238755	RP11-292E2.3	3.53	0.81

ENST00000295927	PTX3-001	ENSG00000163661	PTX3	2.42	0.95
ENST00000468627	RP11-12N13-001	ENSG00000244429	RP11-12N13.1	2.26	0.35
ENST00000420541	TNFSF10-002	ENSG00000121858	TNFSF10	8.08	1.86
ENST00000357390	MFN1-002	ENSG00000171109	MFN1	3.27	3.17
ENST00000483867	RP11-145M9-001	ENSG00000181260	RP11-145M9.2	2.19	1.43
ENST00000265598	LAMP3-001	ENSG00000078081	LAMP3	6.10	1.77
ENST00000259030	RTP4-001	ENSG00000136514	RTP4	4.23	0.29
ENST00000315470	C3orf59-201	ENSG00000180611	C3orf59	2.40	1.99
ENST00000489628	TNK2-018	ENSG00000061938	TNK2	1.90	3.24
ENST00000430929	TNK2-017	ENSG00000061938	TNK2	2.34	5.12
ENST00000502036	AC124944-201	ENSG00000245211	AC124944.4	2.09	4.68
ENST00000424819	AC124944-001	ENSG00000226155	AC124944.1	2.69	1.82
ENST00000397544	ZDHHC19-001	ENSG00000163958	ZDHHC19	-2.95	2.00
ENST00000497422	ZDHHC19-005	ENSG00000163958	ZDHHC19	-3.52	1.81
ENST00000486020	AC069257-201	ENSG00000244636	AC069257.4	-2.55	1.96
ENST00000441275	BDH1-002	ENSG00000161267	BDH1	-2.71	1.51
ENST00000500402	AC128709-201	ENSG00000247754	AC128709.2	-2.58	1.20
ENST00000273582	KIAA0226-001	ENSG00000145016	KIAA0226	2.85	0.38
ENST00000455374	KIAA0226-203	ENSG00000145016	KIAA0226	2.61	5.13
ENST00000337190	MXD4-201	ENSG00000123933	MXD4	-2.17	0.61
ENST00000360265	AFAP1-001	ENSG00000196526	AFAP1	4.30	4.53
ENST00000226279	CD38-001	ENSG00000004468	CD38	9.56	1.91
ENST00000226299	LAP3-001	ENSG00000002549	LAP3	3.77	0.49
ENST00000382114	LGI2-001	ENSG00000153012	LGI2	-3.28	1.75
ENST00000282970	LGI2-201	ENSG00000153012	LGI2	-3.66	1.75
ENST00000264864	PI4K2B-001	ENSG00000038210	PI4K2B	2.64	0.84
ENST00000303965	ARAP2-001	ENSG00000047365	ARAP2	2.48	0.78
ENST00000381799	RHOH-001	ENSG00000168421	RHOH	3.80	0.94
ENST00000248706	RASL11B-001	ENSG00000128045	RASL11B	-3.16	0.38
ENST00000443143	AC116049-201	ENSG00000236562	AC116049.2	4.06	0.63
ENST00000417478	RUFY3-201	ENSG00000018189	RUFY3	2.12	3.50
ENST00000307407	IL8-001	ENSG00000169429	IL8	6.84	1.78
ENST00000395761	CXCL1-201	ENSG00000163739	CXCL1	4.70	1.22
ENST00000296026	CXCL3-201	ENSG00000163734	CXCL3	4.19	1.13
ENST00000264492	CXCL2-201	ENSG00000081041	CXCL2	3.56	1.01
ENST00000500608	AC093677-201	ENSG00000245879	AC093677.1	2.32	0.96
ENST00000244869	EREG-001	ENSG00000124882	EREG	6.13	1.45
ENST00000335927	RASGEF1B-202	ENSG00000138670	RASGEF1B	2.41	0.88
ENST00000454730	HPSE-201	ENSG00000173083	HPSE	2.85	0.56
ENST00000264409	AGPAT9-002	ENSG00000138678	AGPAT9	-3.07	3.74
ENST00000295908	PPM1K-001	ENSG00000163644	PPM1K	4.56	0.34
ENST00000380265	HERC6-203	ENSG00000138642	HERC6	5.13	5.67
ENST00000264350	HERC5-001	ENSG00000138646	HERC5	7.64	0.62
ENST00000296414	DAPP1-201	ENSG00000070190	DAPP1	2.86	0.71
ENST00000356736	SLC39A8-001	ENSG00000138821	SLC39A8	2.33	1.12
ENST00000226574	NFKB1-001	ENSG00000109320	NFKB1	2.31	0.36
ENST00000265171	EGF-001	ENSG00000138798	EGF	2.20	1.37
ENST00000441170	AC092661-002	ENSG00000213492	AC092661.2	5.80	0.60
ENST00000306802	KIAA1109-009	ENSG00000138688	KIAA1109	2.43	1.51
ENST00000320650	IL15-002	ENSG00000164136	IL15	4.55	3.38
ENST00000477265	IL15-003	ENSG00000164136	IL15	4.15	1.96
ENST00000394159	IL15-201	ENSG00000164136	IL15	3.50	3.91
ENST00000296578	OTUD4-201	ENSG00000164164	OTUD4	2.92	7.34
ENST00000500963	AC096757-201	ENSG00000245154	AC096757.3	2.51	2.93
ENST00000411937	DCLK2-204	ENSG00000170390	DCLK2	-4.72	2.80
ENST00000502034	AC106865-201	ENSG00000245126	AC106865.1	2.69	2.20
ENST00000260010	TLR2-201	ENSG00000137462	TLR2	2.54	0.52
ENST00000455639	GUCY1A3-205	ENSG00000164116	GUCY1A3	3.50	1.75
ENST00000264431	RAPGEF2-201	ENSG00000109756	RAPGEF2	2.72	0.95
ENST00000393743	DDX60-202	ENSG00000137628	DDX60	4.47	0.97
ENST00000393739	DDX60-201	ENSG00000137628	DDX60	3.90	0.63
ENST00000260184	DDX60L-201	ENSG00000181381	DDX60L	3.37	0.91
ENST00000457303	AC107214-201	ENSG00000232648	AC107214.3	-2.39	1.22
ENST00000281455	ACSL1-201	ENSG00000151726	ACSL1	2.10	1.36
ENST00000296795	TLR3-201	ENSG00000164342	TLR3	5.61	1.06
ENST00000230859	POLS-001	ENSG00000112941	POLS	2.53	0.53
ENST00000342362	PRLR-203	ENSG00000113494	PRLR	3.06	3.51
ENST00000343305	IL7R-201	ENSG00000168685	IL7R	3.07	6.20
ENST00000303115	IL7R-001	ENSG00000168685	IL7R	3.55	0.45
ENST00000339788	DAB2-202	ENSG00000153071	DAB2	-3.68	4.76
ENST00000302472	PTGER4-001	ENSG00000171522	PTGER4	2.39	0.63

ENST00000388827	CCDC152-202	ENSG00000198865	CCDC152	-4.56	2.60
ENST00000500570	AC008875-201	ENSG00000245900	AC008875.2	2.70	0.34
ENST00000296585	ITGA2-001	ENSG00000164171	ITGA2	3.31	1.42
ENST00000424459	GPBP1-203	ENSG00000062194	GPBP1	2.41	3.20
ENST00000296596	ELOVL7-201	ENSG00000164181	ELOVL7	2.53	2.31
ENST00000340159	NLN-201	ENSG00000123213	NLN	2.36	3.15
ENST00000256447	CD180-001	ENSG00000134061	CD180	-3.03	0.80
ENST00000322348	GCNT4-001	ENSG00000176928	GCNT4	4.46	1.57
ENST00000442824	AC116347-201	ENSG00000238000	AC116347.1	2.92	0.43
ENST00000427199	TICAM2-001	ENSG00000243414	TICAM2	1.96	0.81
ENST00000261366	LMNB1-001	ENSG00000113368	LMNB1	2.86	0.50
ENST00000472034	LMNB1-004	ENSG00000113368	LMNB1	4.10	3.60
ENST00000484340	LMNB1-008	ENSG00000113368	LMNB1	3.03	1.97
ENST00000200652	SLC22A4-001	ENSG00000197208	SLC22A4	3.23	0.71
ENST00000378953	C5orf56-002	ENSG00000197536	C5orf56	3.10	2.24
ENST00000337752	C5orf56-001	ENSG00000197536	C5orf56	3.52	0.45
ENST00000461203	C5orf56-004	ENSG00000197536	C5orf56	3.69	1.36
ENST00000443093	AC116366-001	ENSG00000234290	AC116366.1	3.83	2.31
ENST00000245414	IRF1-001	ENSG00000125347	IRF1	3.41	0.72
ENST00000421011	IRF1-201	ENSG00000125347	IRF1	3.60	0.30
ENST00000405885	IRF1-008	ENSG00000125347	IRF1	3.72	0.39
ENST00000378679	SHROOM1-001	ENSG00000164403	SHROOM1	-3.49	2.89
ENST00000433412	PHF15-203	ENSG00000043143	PHF15	3.81	2.40
ENST00000395003	PHF15-002	ENSG00000043143	PHF15	3.18	0.45
ENST00000470876	PHF15-004	ENSG00000043143	PHF15	3.43	1.76
ENST00000430087	PHF15-006	ENSG00000043143	PHF15	3.45	2.97
ENST00000449031	C5orf20-001	ENSG00000235739	C5orf20	2.21	0.46
ENST00000499862	AC135457-201	ENSG00000246304	AC135457.3	2.69	0.85
ENST00000417127	TNIP1-203	ENSG00000145901	TNIP1	2.05	3.63
ENST00000435489	NIPAL4-202	ENSG00000172548	NIPAL4	3.12	2.74
ENST00000499754	AC020894-201	ENSG00000244960	AC020894.1	-3.79	2.33
ENST00000046794	LCP2-201	ENSG00000043462	LCP2	2.26	0.49
ENST00000311601	SH3PXD2B-201	ENSG00000174705	SH3PXD2B	2.76	0.51
ENST00000390654	COL23A1-001	ENSG00000050767	COL23A1	-2.22	2.92
ENST00000361132	RASGEF1C-002	ENSG00000146090	RASGEF1C	-2.66	1.87
ENST00000412334	IRF4-201	ENSG00000137265	IRF4	3.74	4.05
ENST00000500521	AL357054-201	ENSG00000247005	AL357054.2	8.25	1.38
ENST00000380874	FOXC1-001	ENSG00000054598	FOXC1	3.85	1.20
ENST00000499208	AL133351-201	ENSG00000247767	AL133351.2	4.49	1.62
ENST00000380698	SERPINB9-001	ENSG00000170542	SERPINB9	4.25	0.60
ENST00000380409	RIPK1-201	ENSG00000137275	RIPK1	2.59	3.57
ENST00000441301	F13A1-203	ENSG00000124491	F13A1	-6.12	2.01
ENST00000379802	DSP-001	ENSG00000096696	DSP	2.65	2.19
ENST00000379597	GCNT2-003	ENSG00000111846	GCNT2	2.36	2.34
ENST00000339240	NEDD9-201	ENSG00000111859	NEDD9	4.54	3.56
ENST00000399469	HIVEP1-003	ENSG00000095951	HIVEP1	2.63	2.41
ENST00000379375	EDN1-001	ENSG00000078401	EDN1	5.74	1.99
ENST00000259727	GMPR-001	ENSG00000137198	GMPR	6.27	0.56
ENST00000424923	DEK-202	ENSG00000124795	DEK	2.31	3.74
ENST00000429054	RNF144B-202	ENSG00000137393	RNF144B	2.81	0.37
ENST00000259939	RNF144B-001	ENSG00000137393	RNF144B	2.92	0.50
ENST00000259698	FAM65B-001	ENSG00000111913	FAM65B	7.50	1.07
ENST00000397093	RP3-522P13-001	ENSG00000213972	RP3-522P13.3	-3.53	1.54
ENST00000349458	TRIM38-201	ENSG00000112343	TRIM38	2.32	0.87
ENST00000289361	BTN3A1-001	ENSG00000026950	BTN3A1	2.44	3.87
ENST00000480110	BTN3A3-003	ENSG00000111801	BTN3A3	2.68	2.82
ENST00000490254	BTN3A3-011	ENSG00000111801	BTN3A3	2.18	3.50
ENST00000482257	HLA-F-009	ENSG00000204642	HLA-F	2.63	0.43
ENST00000485513	HLA-F-011	ENSG00000204642	HLA-F	3.49	3.98
ENST00000429294	HLA-F-006	ENSG00000204642	HLA-F	2.17	0.53
ENST00000484704	HLA-F-012	ENSG00000204642	HLA-F	4.15	3.38
ENST00000441380	IFITM4P-001	ENSG00000235821	IFITM4P	4.44	0.94
ENST00000430151	HLA-K-001	ENSG00000230795	HLA-K	2.18	3.90
ENST00000454678	TRIM26-002	ENSG00000234127	TRIM26	3.11	3.87
ENST00000480999	TRIM26-004	ENSG00000234127	TRIM26	2.71	4.39
ENST00000493699	HLA-E-003	ENSG00000204592	HLA-E	2.28	0.97
ENST00000399196	C6orf214-001	ENSG00000214894	C6orf214	2.75	1.52
ENST00000412585	HLA-B-001	ENSG00000234745	HLA-B	1.03	0.34
ENST00000383320	HCP5-201	ENSG00000206337	HCP5	3.60	1.73
ENST00000449264	TNF-001	ENSG00000232810	TNF	5.77	0.87
ENST00000419679	XXbac-BPG32J3-001	ENSG00000235663	XXbac-BPG32J3.1	-2.60	1.68

ENST00000375651	HSPA1A-001	ENSG00000204389	HSPA1A	2.16	0.72
ENST00000374897	TAP2-001	ENSG00000204267	TAP2	3.48	0.44
ENST00000428917	TAP2-201	ENSG00000204267	TAP2	2.98	0.57
ENST00000374882	PSMB8-002	ENSG00000204264	PSMB8	2.13	0.37
ENST00000354258	TAP1-001	ENSG00000168394	TAP1	4.28	0.43
ENST00000415067	XXbac-BPG246D15-006	ENSG00000204261	XXbac-BPG246D15.6	3.94	1.89
ENST00000414474	PSMB9-006	ENSG00000240065	PSMB9	4.13	5.31
ENST00000453265	PSMB9-201	ENSG00000240065	PSMB9	3.60	0.71
ENST00000374859	PSMB9-001	ENSG00000240065	PSMB9	3.57	0.43
ENST00000341486	HLA-DMA-201	ENSG00000204257	HLA-DMA	-2.98	3.96
ENST00000454398	HLA-DPA3-001	ENSG00000237398	HLA-DPA3	4.87	1.44
ENST00000339867	RP3-391O22-001	ENSG00000196114	RP3-391O22.1	3.96	0.88
ENST00000316637	DEF6-001	ENSG00000023892	DEF6	-2.70	0.71
ENST00000454686	RP1-193M11-001	ENSG00000213500	RP1-193M11.1	3.99	0.80
ENST00000373451	FTSJD2-001	ENSG00000137200	FTSJD2	2.68	0.34
ENST00000475364	FTSJD2-004	ENSG00000137200	FTSJD2	3.03	5.27
ENST00000297153	MDGA1-201	ENSG00000112139	MDGA1	4.36	3.23
ENST00000373401	MDGA1-001	ENSG00000112139	MDGA1	4.06	2.77
ENST00000437044	TREML1-203	ENSG00000161911	TREML1	-2.72	2.00
ENST00000446507	PTCRA-203	ENSG00000171611	PTCRA	-2.48	0.83
ENST00000393882	GTPBP2-203	ENSG00000172432	GTPBP2	2.12	5.65
ENST00000478660	RUNX2-001	ENSG00000124813	RUNX2	3.23	2.34
ENST00000416077	RP3-437C15-001	ENSG00000232702	RP3-437C15.1	3.07	1.03
ENST00000370336	DDX43-001	ENSG00000080007	DDX43	2.51	1.20
ENST00000370315	C6orf150-002	ENSG00000164430	C6orf150	3.43	0.34
ENST00000336032	PNRC1-001	ENSG00000146278	PNRC1	3.02	0.51
ENST00000368850	SLC16A10-002	ENSG00000112394	SLC16A10	-2.81	1.75
ENST00000359831	TRAF3IP2-005	ENSG00000056972	TRAF3IP2	3.04	4.47
ENST00000359564	DSE-201	ENSG00000111817	DSE	2.33	1.42
ENST00000368605	FAM26F-001	ENSG00000188820	FAM26F	7.45	1.83
ENST00000368604	FAM26F-003	ENSG00000188820	FAM26F	4.78	3.48
ENST00000368123	AKAP7-001	ENSG00000118507	AKAP7	2.12	2.06
ENST00000237289	TNFAIP3-001	ENSG00000118503	TNFAIP3	3.92	0.69
ENST00000012134	HIVEP2-002	ENSG00000010818	HIVEP2	3.11	4.41
ENST00000367582	PHACTR2-201	ENSG00000112419	PHACTR2	2.25	2.98
ENST00000367568	STX11-001	ENSG00000135604	STX11	3.30	0.77
ENST00000416573	ZC3H12D-202	ENSG00000178199	ZC3H12D	2.34	4.29
ENST00000409694	SYNE1-015	ENSG00000131018	SYNE1	-2.48	2.91
ENST00000265198	IPCEF1-001	ENSG00000074706	IPCEF1	-3.10	2.14
ENST00000462408	TIAM2-001	ENSG00000146426	TIAM2	2.22	0.56
ENST00000367089	DYNLT1-001	ENSG00000146425	DYNLT1	2.28	0.22
ENST00000367066	TAGAP-001	ENSG00000164691	TAGAP	3.42	0.40
ENST00000367061	TAGAP-201	ENSG00000164691	TAGAP	4.12	0.90
ENST00000501526	AL035530-201	ENSG00000246620	AL035530.1	2.99	0.59
ENST00000499555	AL035530-201	ENSG00000245892	AL035530.2	2.39	0.91
ENST00000430078	RP3-393E18-001	ENSG00000237927	RP3-393E18.1	2.86	1.02
ENST00000427974	RP1-56L9-001	ENSG00000224073	RP1-56L9.2	3.27	1.19
ENST00000367055	SOD2-002	ENSG00000112096	SOD2	3.98	0.80
ENST00000337404	SOD2-001	ENSG00000112096	SOD2	4.00	1.32
ENST00000499116	AL135914-201	ENSG00000248087	AL135914.3	4.06	0.72
ENST00000337387	WTAP-002	ENSG00000146457	WTAP	3.03	0.57
ENST00000462110	WTAP-003	ENSG00000146457	WTAP	2.73	3.52
ENST00000297468	GPR146-201	ENSG00000164849	GPR146	-2.89	3.04
ENST00000396946	CARD11-011	ENSG00000198286	CARD11	-2.95	0.59
ENST00000223122	C1GALT1-001	ENSG00000106392	C1GALT1	2.21	0.68
ENST00000420252	AC013470-001	ENSG00000236048	AC013470.3	2.67	0.28
ENST00000297029	SCIN-001	ENSG00000006747	SCIN	3.20	2.74
ENST00000445618	SCIN-201	ENSG00000006747	SCIN	2.58	3.86
ENST00000435131	IGF2BP3-202	ENSG00000136231	IGF2BP3	5.48	1.84
ENST00000056233	NFE2L3-001	ENSG00000050344	NFE2L3	3.60	0.76
ENST00000338523	SNX10-002	ENSG00000086300	SNX10	2.58	4.05
ENST00000446848	SNX10-201	ENSG00000086300	SNX10	3.24	0.68
ENST00000451368	AC004540-001	ENSG00000225792	AC004540.2	2.36	0.59
ENST00000409290	WIPF3-002	ENSG00000122574	WIPF3	-2.40	1.33
ENST00000430537	AC007036-001	ENSG00000227017	AC007036.1	2.28	1.10
ENST00000323037	ZNRF2-001	ENSG00000180233	ZNRF2	2.32	0.71
ENST00000319243	ZNRF2-201	ENSG00000180233	ZNRF2	2.61	0.91
ENST00000442800	AC006027-002	ENSG00000244279	AC006027.2	3.79	2.35
ENST00000444150	AC006978-201	ENSG00000235859	AC006978.1	2.17	0.74
ENST00000242210	NT5C3-001	ENSG00000122643	NT5C3	5.58	2.57
ENST00000415237	AC004988-001	ENSG00000203446	AC004988.1	2.19	0.34

ENST00000242208	INHBA-001	ENSG00000122641	INHBA	3.35	2.16
ENST00000442711	INHBA-002	ENSG00000122641	INHBA	2.95	1.34
ENST00000483585	MYO1G-003	ENSG00000136286	MYO1G	2.47	5.15
ENST00000258775	NACAD-201	ENSG00000136274	NACAD	3.47	2.04
ENST00000297323	ADCY1-001	ENSG00000164742	ADCY1	-2.47	1.41
ENST00000442064	RP4-725G10-001	ENSG00000230191	RP4-725G10.4	5.62	0.93
ENST00000360117	ZNF588-003	ENSG00000196247	ZNF588	2.90	2.27
ENST00000344930	ZNF588-002	ENSG00000196247	ZNF588	3.34	2.30
ENST00000395391	ZNF588-001	ENSG00000196247	ZNF588	3.06	3.08
ENST00000338578	AC006995-201	ENSG00000241849	AC006995.3	8.33	1.11
ENST00000423083	NCF1B-003	ENSG00000182487	NCF1B	5.56	1.15
ENST00000395060	CLIP2-002	ENSG00000106665	CLIP2	-2.88	3.55
ENST00000473759	GTF2I-006	ENSG00000077809	GTF2I	2.12	2.25
ENST00000289473	NCF1-001	ENSG00000158517	NCF1	7.12	0.96
ENST00000486097	NCF1-004	ENSG00000158517	NCF1	7.06	4.04
ENST00000297905	NCF1C-001	ENSG00000165178	NCF1C	7.62	0.93
ENST00000248598	FGL2-001	ENSG00000127951	FGL2	2.25	0.85
ENST00000464213	CD36-014	ENSG00000135218	CD36	-3.52	5.32
ENST00000222390	HGF-001	ENSG00000019991	HGF	-4.37	1.60
ENST00000457544	HGF-005	ENSG00000019991	HGF	-2.36	3.15
ENST00000320415	AC004894-001	ENSG00000213530	AC004894.1	2.29	2.19
ENST00000265362	SEMA3A-001	ENSG00000075213	SEMA3A	3.21	1.39
ENST00000380050	PFTK1-001	ENSG00000058091	PFTK1	2.41	4.28
ENST00000422095	ANKIB1-002	ENSG00000001629	ANKIB1	2.17	2.77
ENST00000379958	SAMD9-001	ENSG00000205413	SAMD9	5.19	0.61
ENST00000318238	SAMD9L-001	ENSG00000177409	SAMD9L	4.82	0.33
ENST00000394472	SAMD9L-201	ENSG00000177409	SAMD9L	5.12	0.99
ENST00000502018	AC000119-201	ENSG00000246295	AC000119.2	6.29	4.63
ENST00000412190	STAG3-022	ENSG00000066923	STAG3	2.69	0.84
ENST00000262935	AGFG2-201	ENSG00000106351	AGFG2	-2.64	1.49
ENST00000474713	AGFG2-003	ENSG00000106351	AGFG2	-2.89	1.86
ENST00000478730	ORAI2-001	ENSG00000160991	ORAI2	2.44	5.00
ENST00000318724	ATXN7L1-001	ENSG00000146776	ATXN7L1	3.30	2.05
ENST00000478915	ATXN7L1-004	ENSG00000146776	ATXN7L1	2.61	3.18
ENST00000222553	PBEF1-001	ENSG00000105835	PBEF1	3.37	0.76
ENST00000472714	LAMB1-004	ENSG00000091136	LAMB1	2.33	0.82
ENST00000265440	TFEC-001	ENSG00000105967	TFEC	2.43	4.47
ENST00000462828	TFEC-006	ENSG00000105967	TFEC	2.50	0.33
ENST00000452096	TFEC-201	ENSG00000105967	TFEC	3.37	1.09
ENST00000456775	ST7OT1-001	ENSG00000227199	ST7OT1	3.48	1.16
ENST00000257696	C7orf68-001	ENSG00000135245	C7orf68	-2.54	0.59
ENST00000432045	AC058791-001	ENSG00000226380	AC058791.1	2.63	0.88
ENST00000477620	NUP205-007	ENSG00000155561	NUP205	2.57	1.66
ENST00000242351	ZC3HAV1-001	ENSG00000105939	ZC3HAV1	2.92	0.72
ENST00000460845	ZC3HAV1-004	ENSG00000105939	ZC3HAV1	4.40	2.14
ENST00000499543	AC083868-201	ENSG00000245286	AC083868.2	2.31	0.32
ENST00000429934	RP11-383F6-001	ENSG00000229677	RP11-383F6.1	4.40	1.75
ENST00000448866	TBXAS1-003	ENSG00000059377	TBXAS1	-2.24	0.64
ENST00000473341	PARP12-002	ENSG00000059378	PARP12	2.89	0.67
ENST00000488726	PARP12-003	ENSG00000059378	PARP12	2.37	0.27
ENST00000486511	EPHB6-005	ENSG00000106123	EPHB6	-2.66	2.35
ENST00000494750	GIMAP4-006	ENSG00000133574	GIMAP4	3.48	3.14
ENST00000430830	GIMAP4-202	ENSG00000133574	GIMAP4	2.35	0.27
ENST00000500990	AC069304-201	ENSG00000248010	AC069304.1	2.47	1.69
ENST00000413040	NUB1-001	ENSG00000013374	NUB1	3.08	3.86
ENST00000355851	NUB1-002	ENSG00000013374	NUB1	2.79	1.00
ENST00000468404	NUB1-003	ENSG00000013374	NUB1	2.48	4.67
ENST00000483358	NUB1-008	ENSG00000013374	NUB1	4.18	2.77
ENST00000453527	CTSB-205	ENSG00000164733	CTSB	-1.08	0.60
ENST00000265807	SH2D4A-001	ENSG00000104611	SH2D4A	3.04	1.03
ENST00000306793	GFRA2-201	ENSG00000168546	GFRA2	-3.73	1.48
ENST00000276420	DOK2-001	ENSG00000147443	DOK2	-3.31	0.62
ENST00000256412	ADAMDEC1-001	ENSG00000134028	ADAMDEC1	2.22	0.89
ENST00000330843	RAB11FIP1-202	ENSG00000156675	RAB11FIP1	-3.01	3.89
ENST00000253513	IDO1-201	ENSG00000131203	IDO1	10.80	2.27
ENST00000392961	LYN-202	ENSG00000147507	LYN	3.19	2.44
ENST00000403181	LYN-203	ENSG00000147507	LYN	2.22	0.48
ENST00000452400	NCOA2-202	ENSG00000140396	NCOA2	2.50	1.15
ENST00000379114	IL7-203	ENSG00000104432	IL7	2.73	4.37
ENST00000327835	ZNF704-201	ENSG00000164684	ZNF704	-2.50	1.67
ENST00000220751	RIPK2-201	ENSG00000104312	RIPK2	2.92	0.55

ENST00000409330	NBN-003	ENSG00000104320	NBN	4.07	3.11
ENST00000436771	C8orf56-201	ENSG00000236939	C8orf56	5.52	1.75
ENST00000395861	BAALC-205	ENSG00000164929	BAALC	3.85	1.96
ENST00000309982	BAALC-203	ENSG00000164929	BAALC	3.95	1.45
ENST00000239690	NUDCD1-201	ENSG00000120526	NUDCD1	2.81	3.75
ENST00000378204	EXT1-001	ENSG00000182197	EXT1	6.28	0.91
ENST00000437196	EXT1-004	ENSG00000182197	EXT1	6.79	2.03
ENST00000286234	DEPDC6-201	ENSG00000155792	DEPDC6	-5.05	1.60
ENST00000399018	FER1L6-201	ENSG00000214814	FER1L6	2.37	1.19
ENST00000301258	PSCA-201	ENSG00000167653	PSCA	-3.05	1.19
ENST00000292494	LY6E-201	ENSG00000160932	LY6E	2.84	0.93
ENST00000429120	LY6E-202	ENSG00000160932	LY6E	2.64	1.08
ENST00000436759	PLEC1-209	ENSG00000178209	PLEC1	-1.35	0.61
ENST00000313028	PARP10-201	ENSG00000178685	PARP10	2.81	0.70
ENST00000424149	CYHR1-204	ENSG00000187954	CYHR1	-2.64	2.09
ENST00000483757	DOCK8-004	ENSG00000107099	DOCK8	2.22	1.06
ENST00000426054	DOCK8-203	ENSG00000107099	DOCK8	2.43	1.08
ENST00000381652	JAK2-001	ENSG00000096968	JAK2	3.23	0.92
ENST00000381577	CD274-001	ENSG00000120217	CD274	5.09	0.57
ENST00000397747	PDCD1LG2-001	ENSG00000197646	PDCD1LG2	3.27	2.95
ENST00000500325	AL161909-201	ENSG00000246447	AL161909.2	-2.86	1.13
ENST00000495827	PTPLAD2-003	ENSG00000188921	PTPLAD2	2.64	2.44
ENST00000431203	IFNWP2-201	ENSG00000237691	IFNWP2	2.63	2.06
ENST00000276927	IFNA1-001	ENSG00000197919	IFNA1	9.85	1.58
ENST00000453177	RP11-370B11-001	ENSG00000236739	RP11-370B11.1	2.48	0.50
ENST00000379882	DDX58-201	ENSG00000107201	DDX58	5.82	1.28
ENST00000379868	DDX58-002	ENSG00000107201	DDX58	7.35	4.43
ENST00000330899	DNAJA1-001	ENSG00000086061	DNAJA1	2.25	0.38
ENST00000486056	CREB3-002	ENSG00000107175	CREB3	1.97	0.89
ENST00000486387	CBWD7-003	ENSG00000215126	CBWD7	2.32	1.53
ENST00000446290	RP11-274B18-002	ENSG00000234506	RP11-274B18.1	2.82	0.79
ENST00000436315	RP11-274B18-001	ENSG00000224025	RP11-274B18.2	2.24	1.57
ENST00000377245	TJP2-003	ENSG00000119139	TJP2	3.14	0.83
ENST00000490113	RFK-004	ENSG00000135002	RFK	2.17	2.77
ENST00000343150	CTSL1-001	ENSG00000135047	CTSL1	2.27	6.43
ENST00000343780	SEMA4D-001	ENSG00000187764	SEMA4D	4.15	3.04
ENST00000422704	SEMA4D-005	ENSG00000187764	SEMA4D	4.13	0.80
ENST00000499581	AL929575-201	ENSG00000247593	AL929575.1	3.67	2.14
ENST00000500574	AL353764-201	ENSG00000245383	AL353764.1	2.42	0.76
ENST00000355295	TDRD7-001	ENSG00000196116	TDRD7	3.33	1.89
ENST00000422139	TDRD7-201	ENSG00000196116	TDRD7	1.99	3.06
ENST00000375018	ANKS6-201	ENSG00000165138	ANKS6	-2.49	2.22
ENST00000238459	NR4A3-201	ENSG00000119508	NR4A3	2.26	2.00
ENST00000330847	NR4A3-003	ENSG00000119508	NR4A3	1.95	2.29
ENST00000361820	GRIN3A-001	ENSG00000198785	GRIN3A	3.38	0.50
ENST00000497048	KLF4-001	ENSG00000136826	KLF4	2.67	1.76
ENST00000337530	KIAA1958-001	ENSG00000165185	KIAA1958	3.15	0.58
ENST00000374244	KIAA1958-002	ENSG00000165185	KIAA1958	3.48	1.85
ENST00000454585	C9orf110-201	ENSG00000225684	C9orf110	2.46	0.61
ENST00000453010	C9orf109-001	ENSG00000231528	C9orf109	2.43	0.29
ENST00000288466	ZNF618-201	ENSG00000157657	ZNF618	2.77	0.39
ENST00000374126	ZNF618-004	ENSG00000157657	ZNF618	3.20	5.15
ENST00000448809	C9orf91-201	ENSG00000157693	C9orf91	2.99	0.44
ENST00000482552	C9orf91-004	ENSG00000157693	C9orf91	3.61	2.87
ENST00000427842	TNFSF15-201	ENSG00000181634	TNFSF15	4.79	1.59
ENST00000373887	TRAF1-001	ENSG00000056558	TRAF1	4.54	1.08
ENST00000223642	C5-001	ENSG00000106804	C5	4.09	0.74
ENST00000286713	STOM-001	ENSG00000148175	STOM	2.16	0.35
ENST00000481799	GGTA1-005	ENSG00000204136	GGTA1	-3.21	1.20
ENST00000373782	DAB2IP-010	ENSG00000136848	DAB2IP	3.27	2.43
ENST00000361171	FAM125B-001	ENSG00000196814	FAM125B	3.02	0.36
ENST00000373176	AK1-008	ENSG00000106992	AK1	-3.11	0.53
ENST00000455981	RP11-344B5-001	ENSG00000224307	RP11-344B5.3	-4.04	1.16
ENST00000444125	RP11-65J3-003	ENSG00000233901	RP11-65J3.4	-2.72	2.45
ENST00000501138	AL353803-201	ENSG00000245107	AL353803.2	-2.56	1.53
ENST00000259339	TOR1B-001	ENSG00000136816	TOR1B	3.37	0.33
ENST00000420546	RP13-100B2-001	ENSG00000227898	RP13-100B2.1	2.78	2.02
ENST00000371899	SLC2A6-001	ENSG00000160326	SLC2A6	4.11	1.11
ENST00000485978	SLC2A6-003	ENSG00000160326	SLC2A6	4.22	2.22
ENST00000371600	NPDC1-002	ENSG00000107281	NPDC1	-2.73	1.41
ENST00000491734	EXD3-007	ENSG00000187609	EXD3	-3.58	2.50

ENST00000479452	EXD3-005	ENSG00000187609	EXD3	-2.65	3.52
ENST00000341349	NOXA1-001	ENSG00000188747	NOXA1	-2.98	0.78
ENST00000392815	NOXA1-201	ENSG00000188747	NOXA1	-2.51	2.58
ENST00000492278	PNPLA7-002	ENSG00000130653	PNPLA7	-2.81	1.24
ENST00000469998	PNPLA7-003	ENSG00000130653	PNPLA7	-2.46	3.58
ENST00000406427	PNPLA7-204	ENSG00000130653	PNPLA7	-3.00	2.46
ENST00000361899	J01415-201	ENSG00000198899	J01415.24	-0.80	0.30
ENST00000399012	PLCXD1-001	ENSG00000182378	PLCXD1	-3.05	1.90
ENST00000427886	RP13-297E16-001	ENSG00000223511	RP13-297E16.1	3.71	1.17
ENST00000380693	MSL3-010	ENSG00000005302	MSL3	3.08	3.61
ENST00000380064	REPS2-202	ENSG00000169891	REPS2	-2.62	3.11
ENST00000469714	REPS2-004	ENSG00000169891	REPS2	-3.18	1.98
ENST00000366134	RP13-314C10-001	ENSG00000233785	RP13-314C10.1	4.02	1.56
ENST00000422221	RP11-469E19-001	ENSG00000237931	RP11-469E19.1	2.56	0.64
ENST00000453805	USP9X-006	ENSG00000124486	USP9X	2.51	7.01
ENST00000378138	GPR34-002	ENSG00000171659	GPR34	-4.18	3.04
ENST00000414389	KDM6A-002	ENSG00000147050	KDM6A	2.85	3.34
ENST00000431196	KDM6A-008	ENSG00000147050	KDM6A	2.48	1.19
ENST00000330258	FAM123B-002	ENSG00000184675	FAM123B	2.61	0.61
ENST00000373177	HDX-001	ENSG00000165259	HDX	3.90	1.04
ENST00000399417	RP1-320J15-001	ENSG00000215007	RP1-320J15.1	2.35	0.57
ENST00000361407	MID2-201	ENSG00000080561	MID2	2.77	2.74
ENST00000420625	PLS3-201	ENSG00000102024	PLS3	3.24	2.05
ENST00000429967	APLN-001	ENSG00000171388	APLN	-2.71	2.44
ENST00000371064	ZDHHC9-005	ENSG00000188706	ZDHHC9	2.63	5.46
ENST00000427391	RP11-453F18__B-001	ENSG00000213468	RP11-453F18__B.1	2.31	0.35
ENST00000475361	FAM122C-006	ENSG00000156500	FAM122C	2.32	0.91
ENST00000379410	PLEKHN1-001	ENSG00000187583	PLEKHN1	2.78	4.05
ENST00000491024	PLEKHN1-003	ENSG00000187583	PLEKHN1	3.11	2.47
ENST00000461111	AGRN-008	ENSG00000188157	AGRN	3.04	4.52
ENST00000475369	C1orf224-002	ENSG00000237276	C1orf224	5.29	0.83
ENST00000482359	ARHGEF10L-008	ENSG00000074964	ARHGEF10L	2.21	3.06
ENST00000339145	IFI6-003	ENSG00000126709	IFI6	4.18	5.56
ENST00000373456	RNF19B-001	ENSG00000116514	RNF19B	2.33	4.87
ENST00000475935	ADC-007	ENSG00000142920	ADC	3.55	2.34
ENST00000471012	ZC3H12A-005	ENSG00000163874	ZC3H12A	3.17	3.69
ENST00000262675	TTC39A-001	ENSG00000085831	TTC39A	2.35	2.08
ENST00000447329	RP4-794H19-002	ENSG00000230812	RP4-794H19.4	3.64	1.17
ENST00000438195	RP4-794H19-001	ENSG00000230812	RP4-794H19.4	3.91	1.39
ENST00000407417	NFIA-203	ENSG00000162599	NFIA	-2.74	2.99
ENST00000371045	PDE4B-007	ENSG00000184588	PDE4B	5.93	1.88
ENST00000342754	NEXN-005	ENSG00000162614	NEXN	7.31	2.21
ENST00000486882	IFI44L-002	ENSG00000137959	IFI44L	8.94	6.56
ENST00000446486	IFI44-009	ENSG00000137965	IFI44	4.42	4.55
ENST00000370608	MCOLN2-201	ENSG00000153898	MCOLN2	5.34	2.37
ENST00000417291	GBP5-202	ENSG00000154451	GBP5	6.85	0.82
ENST00000471903	FAM72B-006	ENSG00000188610	FAM72B	2.33	1.19
ENST00000425004	FAM72D-201	ENSG00000215784	FAM72D	3.09	2.13
ENST00000331128	HIST2H2AB-001	ENSG00000184270	HIST2H2AB	3.29	1.17
ENST00000452671	RFX5-002	ENSG00000143390	RFX5	2.30	2.56
ENST00000341831	ATP8B2-202	ENSG00000143515	ATP8B2	2.25	3.11
ENST00000292254	RUSC1-005	ENSG00000160753	RUSC1	2.21	5.29
ENST00000492592	ARHGEF11-007	ENSG00000132694	ARHGEF11	2.91	3.97
ENST00000368130	AIM2-001	ENSG00000163568	AIM2	8.87	1.02
ENST00000302035	SLAMF1-001	ENSG00000117090	SLAMF1	4.63	1.86
ENST00000458602	SLAMF7-205	ENSG00000026751	SLAMF7	2.39	10.59
ENST00000444639	KIAA0040-001	ENSG00000235750	KIAA0040	4.17	2.68
ENST00000366910	MOSC1-001	ENSG00000186205	MOSC1	-2.76	2.51
ENST00000319465	IL15RA-002	ENSG00000134470	IL15RA	4.87	3.79
ENST00000358499	PFKFB3-003	ENSG00000170525	PFKFB3	3.64	7.36
ENST00000378747	OPTN-008	ENSG00000123240	OPTN	4.43	4.42
ENST00000430603	MAP3K8-001	ENSG00000107968	MAP3K8	2.43	2.84
ENST00000374426	CXCL12-003	ENSG00000107562	CXCL12	4.08	3.19
ENST00000374370	ANUBL1-006	ENSG00000172671	ANUBL1	2.30	1.97
ENST00000399298	TMEM26-202	ENSG00000196932	TMEM26	2.25	1.34
ENST00000354393	MYPN-002	ENSG00000138347	MYPN	2.53	1.90
ENST00000458208	ACTA2-201	ENSG00000107796	ACTA2	2.22	0.59
ENST00000371697	ANKRD1-001	ENSG00000148677	ANKRD1	6.00	1.86
ENST00000370495	SLC25A28-001	ENSG00000155287	SLC25A28	2.50	4.30
ENST00000370005	ELOVL3-001	ENSG00000119915	ELOVL3	4.79	0.99
ENST00000454143	NFKB2-204	ENSG00000077150	NFKB2	2.94	4.66

ENST00000348655	IRF7-003	ENSG00000185507	IRF7	6.04	3.14
ENST00000397566	IRF7-202	ENSG00000185507	IRF7	2.75	2.11
ENST00000330243	IRF7-001	ENSG00000185507	IRF7	3.54	1.14
ENST00000380725	CDKN1C-002	ENSG00000129757	CDKN1C	2.25	2.45
ENST00000396847	TRIM5-004	ENSG00000132256	TRIM5	3.72	3.06
ENST00000394613	PLA2G16-202	ENSG00000176485	PLA2G16	3.85	2.18
ENST00000313599	CD163L1-201	ENSG00000177675	CD163L1	-3.66	3.53
ENST00000299665	CLEC4D-201	ENSG00000166527	CLEC4D	3.81	1.27
ENST00000478250	NR4A1-001	ENSG00000123358	NR4A1	-3.40	2.68
ENST00000416617	IQCD-203	ENSG00000166578	IQCD	-2.61	2.07
ENST00000413816	GLT1D1-202	ENSG00000151948	GLT1D1	2.44	2.56
ENST00000439658	C14orf182-202	ENSG00000214900	C14orf182	2.74	2.12
ENST00000361529	C14orf68-201	ENSG00000140107	C14orf68	3.80	1.33
ENST00000348956	CKB-201	ENSG00000166165	CKB	4.11	3.01
ENST00000392593	PLD4-001	ENSG00000166428	PLD4	3.47	2.83
ENST00000313559	C15orf21-004	ENSG00000179362	C15orf21	4.49	2.70
ENST00000267838	LYSMD2-001	ENSG00000140280	LYSMD2	3.81	2.60
ENST00000499612	AC068327-201	ENSG00000246848	AC068327.1	-4.23	3.11
ENST00000306072	ISG20-001	ENSG00000172183	ISG20	9.27	2.14
ENST00000359595	HAPLN3-001	ENSG00000140511	HAPLN3	7.36	1.78
ENST00000300797	PRRT2-201	ENSG00000167371	PRRT2	3.89	1.04
ENST00000268459	NKD1-001	ENSG00000140807	NKD1	2.74	1.14
ENST00000308149	NLRC5-201	ENSG00000140853	NLRC5	3.38	3.26
ENST00000314553	EXOC3L-201	ENSG00000179044	EXOC3L	4.49	1.52
ENST00000326043	MAF-001	ENSG00000178573	MAF	-2.76	4.43
ENST00000428963	CENPN-201	ENSG00000166451	CENPN	2.35	3.14
ENST00000378553	LRRC50-001	ENSG00000154099	LRRC50	7.81	2.04
ENST00000200307	CCL7-201	ENSG00000108688	CCL7	6.44	2.25
ENST00000394627	CCL7-003	ENSG00000108688	CCL7	5.01	2.28
ENST00000225840	CCL8-201	ENSG00000108700	CCL8	10.60	2.27
ENST00000378354	CCL3L1-201	ENSG00000205021	CCL3L1	5.41	2.34
ENST00000444414	CCL4L1-208	ENSG00000205020	CCL4L1	6.30	1.93
ENST00000378342	CCL4L2-201	ENSG00000197262	CCL4L2	7.27	2.30
ENST00000339270	CCL4L2-002	ENSG00000197262	CCL4L2	7.61	2.04
ENST00000246657	CCR7-001	ENSG00000126353	CCR7	8.82	1.17
ENST00000357037	PTRF-201	ENSG00000177469	PTRF	2.63	2.95
ENST00000225964	COL1A1-001	ENSG00000108821	COL1A1	2.70	1.18
ENST00000393142	SCPEP1-202	ENSG00000121064	SCPEP1	2.13	3.78
ENST00000428869	MRO-202	ENSG00000134042	MRO	-2.94	2.79
ENST00000221847	EBI3-201	ENSG00000105246	EBI3	5.00	1.39
ENST00000264825	NFIX-201	ENSG00000008441	NFIX	2.39	6.47
ENST00000253680	HSH2D-201	ENSG00000196684	HSH2D	6.38	2.23
ENST00000455515	FXYD1-202	ENSG00000221857	FXYD1	-2.96	2.80
ENST00000474300	BCL3-006	ENSG00000069399	BCL3	2.18	1.14
ENST00000263265	PLEKHA4-201	ENSG00000105559	PLEKHA4	8.03	2.74
ENST00000221399	TULP2-201	ENSG00000104804	TULP2	3.38	2.09
ENST00000358789	KLK10-202	ENSG00000129451	KLK10	3.18	2.26
ENST00000270458	CACNG8-001	ENSG00000142408	CACNG8	5.55	1.61
ENST00000448689	LILRB1-204	ENSG00000104972	LILRB1	3.33	4.38
ENST00000381465	TRIB2-002	ENSG00000071575	TRIB2	4.08	1.52
ENST00000339598	OTOF-003	ENSG00000115155	OTOF	6.91	1.71
ENST00000302188	RBKS-001	ENSG00000171174	RBKS	2.31	2.03
ENST00000436647	FOSL2-004	ENSG00000075426	FOSL2	2.19	2.77
ENST00000462451	VAMP5-002	ENSG00000168899	VAMP5	3.56	2.20
ENST00000393805	ST3GAL5-003	ENSG00000115525	ST3GAL5	2.15	3.52
ENST00000440974	AC012363-001	ENSG00000227788	AC012363.7	3.02	1.25
ENST00000243347	TNFAIP6-001	ENSG00000123610	TNFAIP6	10.14	1.39
ENST00000418968	AC007750-001	ENSG00000236841	AC007750.1	2.71	1.58
ENST00000415236	AC009948-007	ENSG00000223960	AC009948.3	2.65	2.07
ENST00000428675	PLCL1-006	ENSG00000115896	PLCL1	2.43	3.76
ENST00000470178	CFLAR-022	ENSG00000003402	CFLAR	3.03	3.00
ENST00000433692	AC007283-002	ENSG00000234431	AC007283.2	2.42	2.37
ENST00000392263	CASP8-009	ENSG00000064012	CASP8	2.40	4.84
ENST00000374155	RUFY4-002	ENSG00000188282	RUFY4	4.57	2.07
ENST00000419663	GPBAR1-202	ENSG00000179921	GPBAR1	2.66	4.97
ENST00000444636	SP140L-004	ENSG00000185404	SP140L	2.83	3.35
ENST00000458341	SP140L-001	ENSG00000185404	SP140L	2.31	2.62
ENST00000258381	SP110-002	ENSG00000135899	SP110	3.82	3.49
ENST00000484809	SNX5-015	ENSG00000089006	SNX5	-2.39	1.44
ENST00000498178	SRC-012	ENSG00000197122	SRC	2.15	3.52
ENST00000462493	C20orf175-001	ENSG00000042062	C20orf175	2.61	1.71

ENST00000370091	RP4-697K14-202	ENSG00000130589	RP4-697K14.2	7.29	3.02
ENST00000348354	BTG3-002	ENSG00000154640	BTG3	2.81	2.66
ENST00000400558	C21orf91-002	ENSG00000154642	C21orf91	2.55	2.68
ENST00000288383	MX1-003	ENSG00000157601	MX1	7.85	4.30
ENST00000326341	AP000355-001	ENSG00000242786	AP000355.1	2.95	1.17
ENST00000401395	KIAA1671-202	ENSG00000197077	KIAA1671	5.64	1.93
ENST00000349314	APOL3-003	ENSG00000128284	APOL3	5.00	4.04
ENST00000403662	CSF2RB-001	ENSG00000100368	CSF2RB	3.16	3.23
ENST00000462332	GTPBP1-002	ENSG00000100226	GTPBP1	2.96	3.07
ENST00000482294	CBX7-002	ENSG00000100307	CBX7	2.88	0.41
ENST00000273221	IQSEC1-001	ENSG00000144711	IQSEC1	-2.97	3.97
ENST00000441346	AC011816-001	ENSG00000234073	AC011816.4	2.74	2.11
ENST00000307872	TMEM110-001	ENSG00000213533	TMEM110	2.87	4.42
ENST00000264634	WNT5A-201	ENSG00000114251	WNT5A	3.20	3.47
ENST00000495160	HESX1-001	ENSG00000163666	HESX1	7.17	1.46
ENST00000484675	RP11-779P15-001	ENSG00000243089	RP11-779P15.1	2.93	0.99
ENST00000326172	NFKBIZ-003	ENSG00000144802	NFKBIZ	3.89	3.40
ENST00000398258	CD47-001	ENSG00000196776	CD47	1.95	0.91
ENST00000264246	CD80-001	ENSG00000121594	CD80	6.08	2.59
ENST00000383660	PARP15-201	ENSG00000173200	PARP15	3.95	2.49
ENST00000465304	PARP15-005	ENSG00000173200	PARP15	3.17	3.14
ENST00000493645	PARP15-006	ENSG00000173200	PARP15	3.48	2.37
ENST00000347421	ATP2C1-202	ENSG00000017260	ATP2C1	3.12	4.55
ENST00000468985	PLSCR1-008	ENSG00000188313	PLSCR1	4.53	6.63
ENST00000241261	TNFSF10-001	ENSG00000121858	TNFSF10	8.55	2.53
ENST00000325318	TCTEX1D2-001	ENSG00000213123	TCTEX1D2	-2.61	4.20
ENST00000438983	HERC6-204	ENSG00000138642	HERC6	4.05	4.44
ENST00000305798	TSPAN5-001	ENSG00000168785	TSPAN5	2.47	1.00
ENST00000057513	TNIP3-001	ENSG00000050730	TNIP3	7.21	1.38
ENST00000422544	PDGFC-202	ENSG00000145431	PDGFC	-2.87	2.68
ENST00000456232	RICTOR-203	ENSG00000164327	RICTOR	2.19	4.46
ENST00000378947	C5orf56-003	ENSG00000197536	C5orf56	3.59	1.38
ENST00000394020	ADAM19-201	ENSG00000135074	ADAM19	4.13	3.04
ENST00000420981	RP11-420G6-001	ENSG00000230438	RP11-420G6.1	3.61	0.87
ENST00000402854	RP11-367G6-001	ENSG00000220517	RP11-367G6.2	2.76	0.86
ENST00000314332	HIST1H2BC-201	ENSG00000180596	HIST1H2BC	2.49	1.35
ENST00000289316	HIST1H2BD-001	ENSG00000158373	HIST1H2BD	2.39	0.99
ENST00000489638	LTA-003	ENSG00000226979	LTA	4.31	3.34
ENST00000464100	TAP2-004	ENSG00000204267	TAP2	3.39	2.30
ENST00000467593	PSMB9-004	ENSG00000240065	PSMB9	3.65	2.11
ENST00000470273	FGD2-008	ENSG00000146192	FGD2	2.22	3.06
ENST00000354922	PNRC1-002	ENSG00000146278	PNRC1	4.36	2.67
ENST00000492671	TRAF3IP2-009	ENSG00000056972	TRAF3IP2	3.83	3.40
ENST00000473330	FSCN1-002	ENSG00000075618	FSCN1	-2.73	4.20
ENST00000398374	CAMK2B-202	ENSG00000058404	CAMK2B	-3.66	4.31
ENST00000463516	MYO1G-004	ENSG00000136286	MYO1G	2.64	1.37
ENST00000342771	AUTS2-001	ENSG00000158321	AUTS2	3.76	1.91
ENST00000435988	NCF1B-001	ENSG00000182487	NCF1B	7.20	1.05
ENST00000357520	BET1-005	ENSG00000105829	BET1	2.70	1.05
ENST00000413493	MOSPD3-203	ENSG00000106330	MOSPD3	-2.53	2.35
ENST00000474380	LAMB1-012	ENSG00000091136	LAMB1	2.82	2.75
ENST00000458153	MKLN1-003	ENSG00000128585	MKLN1	2.87	4.52
ENST00000463000	WDR86-005	ENSG00000187260	WDR86	3.50	1.22
ENST00000300332	NKX3-1-201	ENSG00000167034	NKX3-1	2.90	1.41
ENST00000265433	NBN-001	ENSG00000104320	NBN	3.00	2.51
ENST00000259555	IFNA13-201	ENSG00000233816	IFNA13	9.95	1.13
ENST00000423632	RP11-402G3-001	ENSG00000230054	RP11-402G3.1	2.25	1.40
ENST00000408936	DAB2IP-009	ENSG00000136848	DAB2IP	4.81	2.11
ENST00000360670	NTNG2-201	ENSG00000196358	NTNG2	3.82	1.89
ENST00000371897	SLC2A6-002	ENSG00000160326	SLC2A6	4.20	2.87
ENST00000381663	PLCXD1-201	ENSG00000182378	PLCXD1	-2.76	2.08
ENST00000462359	SLC38A5-006	ENSG00000017483	SLC38A5	5.87	1.07
ENST00000470657	FAM122C-004	ENSG00000156500	FAM122C	2.50	1.92
ENST00000379236	TNFRSF4-001	ENSG00000186827	TNFRSF4	4.30	2.36
ENST00000414055	C1orf224-201	ENSG00000237276	C1orf224	5.44	1.45
ENST00000373836	PHACTR4-004	ENSG00000204138	PHACTR4	2.87	4.16
ENST00000422952	GBP1-201	ENSG00000117228	GBP1	7.05	3.28
ENST00000490413	MOV10-008	ENSG00000155363	MOV10	2.33	3.27
ENST00000420505	ADAR-203	ENSG00000160710	ADAR	2.28	0.78
ENST00000392405	RUSC1-202	ENSG00000160753	RUSC1	2.07	5.90
ENST00000471382	BATF3-003	ENSG00000123685	BATF3	4.76	2.29

ENST00000435171	IL15RA-009	ENSG00000134470	IL15RA	4.94	1.80
ENST00000459894	SFXN2-002	ENSG00000156398	SFXN2	-2.55	3.13
ENST00000482993	RP11-108K14-004	ENSG00000214279	RP11-108K14.1	2.22	2.29
ENST00000382073	CLEC6A-201	ENSG00000205846	CLEC6A	3.38	1.52
ENST00000432556	OLR1-202	ENSG00000173391	OLR1	2.24	3.33
ENST00000357825	SRGAP1-202	ENSG00000196935	SRGAP1	-2.53	4.78
ENST00000262401	SLC46A1-201	ENSG00000076351	SLC46A1	-2.93	4.83
ENST00000427581	LILRB1-007	ENSG00000104972	LILRB1	4.16	2.99
ENST00000464371	KHK-005	ENSG00000138030	KHK	-3.17	2.39
ENST00000451794	NEURL3-202	ENSG00000163121	NEURL3	8.62	1.43
ENST00000263341	IL1B-001	ENSG00000125538	IL1B	6.91	2.18
ENST00000478233	MAP2-013	ENSG00000078018	MAP2	4.04	1.53
ENST00000413284	SP100-016	ENSG00000067066	SP100	2.44	3.63
ENST00000427522	RP4-697K14-001	ENSG00000130589	RP4-697K14.2	5.35	3.26
ENST00000472328	IMPDH2-004	ENSG00000178035	IMPDH2	-2.57	2.86
ENST00000492922	RP11-413E6-002	ENSG00000242516	RP11-413E6.5	3.49	2.34
ENST00000489652	PARP9-006	ENSG00000138496	PARP9	4.80	4.68
ENST00000471785	PARP9-005	ENSG00000138496	PARP9	4.26	5.46
ENST00000306602	CXCL10-201	ENSG00000169245	CXCL10	11.34	3.01
ENST00000501655	AC025171-201	ENSG00000246776	AC025171.2	3.49	1.38
ENST00000365663	Y_RNA	ENSG00000202533	Y_RNA	2.29	1.59
ENST00000426696	TRIM26-201	ENSG00000234127	TRIM26	2.13	0.91
ENST00000452596	HLA-B-202	ENSG00000234745	HLA-B	2.04	4.97
ENST00000486332	TAP1-002	ENSG00000168394	TAP1	3.85	2.30
ENST00000367054	SOD2-201	ENSG00000112096	SOD2	4.28	2.72
ENST00000436911	TRGC2-001	ENSG00000227191	TRGC2	3.42	0.88
ENST00000431284	AC004847-201	ENSG00000231337	AC004847.1	3.23	2.31
ENST00000398421	NCF1-002	ENSG00000158517	NCF1	7.45	2.25
ENST00000394000	PILRA-005	ENSG00000085514	PILRA	2.97	4.57
ENST00000477392	RP11-511P7-001	ENSG00000242258	RP11-511P7.1	5.46	1.61
ENST00000412660	GIMAP4-201	ENSG00000133574	GIMAP4	2.54	2.26
ENST00000483055	NTNG2-005	ENSG00000196358	NTNG2	4.31	1.31
ENST00000379361	PTCHD1-002	ENSG00000165186	PTCHD1	2.42	2.54
ENST00000474385	ZFX-005	ENSG00000005889	ZFX	2.61	2.14
ENST00000297977	HDX-201	ENSG00000165259	HDX	3.52	1.63
ENST00000497869	TNFRSF4-002	ENSG00000186827	TNFRSF4	3.83	2.02
ENST00000468348	C1orf127-003	ENSG00000175262	C1orf127	-3.47	2.08
ENST00000358481	PADI6-201	ENSG00000197996	PADI6	2.32	2.18
ENST00000466782	ARHGEF10L-009	ENSG00000074964	ARHGEF10L	2.29	3.50
ENST00000447420	RP4-803A2-001	ENSG00000239670	RP4-803A2.2	2.53	2.08
ENST00000484656	ADC-008	ENSG00000142920	ADC	3.25	3.24
ENST00000466619	GSTM1-011	ENSG00000134184	GSTM1	-2.55	3.52
ENST00000391154	U1	ENSG00000212456	U1	2.36	1.11
ENST00000463840	TSPAN15-008	ENSG00000099282	TSPAN15	-2.63	2.01
ENST00000395901	ARNTL2-204	ENSG00000029153	ARNTL2	3.20	4.38
ENST00000378487	CLLU1OS-201	ENSG00000205057	CLLU1OS	2.46	1.57
ENST00000250572	EMR1-201	ENSG00000174837	EMR1	2.42	4.13
ENST00000441512	AC011515-001	ENSG00000225370	AC011515.1	2.86	1.14
ENST00000442815	AP001046-001	ENSG00000237989	AP001046.2	3.55	1.70
ENST00000382723	MSX1-001	ENSG00000163132	MSX1	5.10	1.56
ENST00000360303	SORCS2-202	ENSG00000184985	SORCS2	2.97	1.43
ENST00000407526	GRB10-007	ENSG00000106070	GRB10	2.14	4.21
ENST00000423057	DOCK4-007	ENSG00000128512	DOCK4	2.07	2.81
ENST00000351513	DPYS-201	ENSG00000147647	DPYS	2.26	1.01
ENST00000474239	FBXO6-002	ENSG00000116663	FBXO6	3.38	3.69
ENST00000486901	HSPG2-002	ENSG00000142798	HSPG2	-3.35	2.75
ENST00000370751	IFI44L-001	ENSG00000137959	IFI44L	9.81	1.75
ENST00000415432	VAV3-202	ENSG00000134215	VAV3	2.59	2.90
ENST00000488160	MOV10-013	ENSG00000155363	MOV10	2.57	5.28
ENST00000455004	BX284650-001	ENSG00000231734	BX284650.6	2.35	1.57
ENST00000373214	KIAA1274-001	ENSG00000107719	KIAA1274	-3.86	3.27
ENST00000405308	MDK-006	ENSG00000110492	MDK	2.35	2.99
ENST00000415415	CTSC-205	ENSG00000109861	CTSC	2.49	4.48
ENST00000320621	CTSC-202	ENSG00000109861	CTSC	3.03	4.09
ENST00000203629	LAG3-201	ENSG00000089692	LAG3	7.37	2.19
ENST00000417623	TMEM117-202	ENSG00000139173	TMEM117	-2.56	3.19
ENST00000420463	EIF4B-203	ENSG00000063046	EIF4B	-2.74	5.24
ENST00000432758	TRAFD1-203	ENSG00000135148	TRAFD1	2.57	1.31
ENST00000389812	PLCB2-202	ENSG00000137841	PLCB2	-3.54	6.35
ENST00000343510	EIF2AK2-201	ENSG00000055332	EIF2AK2	5.58	3.68
ENST00000456923	FN1-011	ENSG00000115414	FN1	2.80	3.93

ENST00000382357	OLIG2-001	ENSG00000205927	OLIG2	2.50	2.46
ENST00000482533	RCAN1-006	ENSG00000159200	RCAN1	2.29	5.56
ENST00000383668	CD80-201	ENSG00000121594	CD80	3.67	2.01
ENST00000460683	PARP14-002	ENSG00000173193	PARP14	4.61	5.72
ENST00000476223	RP11-383G6-001	ENSG00000240576	RP11-383G6.3	3.95	1.28
ENST00000306621	CXCL11-001	ENSG00000169248	CXCL11	11.48	2.38
ENST00000284630	DDX60L-202	ENSG00000181381	DDX60L	4.06	3.59
ENST00000428771	HES4-201	ENSG00000188290	HES4	4.32	2.50
ENST00000481869	HES4-003	ENSG00000188290	HES4	6.00	2.41
ENST00000378324	SERPING1-202	ENSG00000149131	SERPING1	3.09	3.54
ENST00000418919	GNS-202	ENSG00000135677	GNS	2.53	4.73
ENST00000357167	BTBD11-004	ENSG00000151136	BTBD11	2.89	2.31
ENST00000300590	SNX20-001	ENSG00000167208	SNX20	2.53	4.62
ENST00000435374	MAP2K6-202	ENSG00000108984	MAP2K6	4.07	2.18
ENST00000409631	OSBPL6-005	ENSG00000079156	OSBPL6	3.15	3.38
ENST00000266041	ITIH4-001	ENSG00000055955	ITIH4	4.03	3.61
ENST00000461966	ITIH4-015	ENSG00000055955	ITIH4	4.33	3.41
ENST00000424970	SLC39A8-201	ENSG00000138821	SLC39A8	3.34	3.77
ENST00000446664	AC006367-201	ENSG00000231695	AC006367.1	4.59	1.91
ENST00000431069	RP11-39K24-001	ENSG00000235917	RP11-39K24.3	3.24	2.28
ENST00000429061	IGHEP2-001	ENSG00000215285	IGHEP2	2.93	2.89
ENST00000405737	ELF1-002	ENSG00000120690	ELF1	3.54	4.30
ENST00000269209	FAM59A-002	ENSG00000141441	FAM59A	3.89	2.20
ENST00000255612	PRAM1-201	ENSG00000133246	PRAM1	-3.13	6.60
ENST00000370097	C20orf195-002	ENSG00000125531	C20orf195	3.27	3.54
ENST00000492928	PLEKHG3-003	ENSG00000126822	PLEKHG3	3.01	3.38
