# Supplementary material for: Identification of Genes Critical for Resistance to Infection by West Nile Virus Using RNA-Seq Analysis
Source: Viruses. 2013 Jul 8;5(7):1664–81. doi: 10.3390/v5071664 (PMC3738954; doi:10.3390/v5071664)
Supplement: Supplementary File 1 — Supplementary (ZIP, 454 KB) [file viruses-05-01664-s001.zip › Qian_Table S4 Annotated differentially expressed HRF and HSF.pdf]

Table S4. Annotated differentially expressed HRF and HSF

HSF

Ensembl.Gene.ID	Assoc.Gene.Name	Description	Ensembl.Transcript.ID	Associated.Transcript.Name	log2 fold change
ENSG00000141837	CACNA1A	calcium channel, voltage-dependent, P/Q type, alpha 1A subunit	ENST00000357018	CACNA1A-202	4.736
ENSG00000173786	CNP	2',3'-cyclic nucleotide 3' phosphodiesterase	ENST00000393888	CNP-002	2.521
ENSG00000196371	FUT4	fucosyltransferase 4 (alpha (1,3) fucosyltransferase, myeloid-specific)	ENST00000358752	FUT4-201	2.993
ENSG00000104951	IL4I1	interleukin 4 induced 1	ENST00000391826	IL4I1-203	4.799
ENSG00000160932	LY6E	lymphocyte antigen 6 complex, locus E	ENST00000292494	LY6E-201	2.836
ENSG00000160932	LY6E	lymphocyte antigen 6 complex, locus E	ENST00000429120	LY6E-202	2.64
ENSG00000140807	NKD1	naked cuticle homolog 1 (Drosophila)	ENST00000268459	NKD1-001	2.744
ENSG00000105559	PLEKHA4	pleckstrin homology domain containing, family A (phosphoinositide binding sp	ENST00000263265	PLEKHA4-201	8.027
ENSG00000130300	PLVAP	plasmalemma vesicle associated protein	ENST00000252590	PLVAP-201	2.83
ENSG00000167653	PSCA	prostate stem cell antigen	ENST00000301258	PSCA-201	-3.053
ENSG00000006747	SCIN	scinderin	ENST00000297029	SCIN-001	3.2
ENSG00000006747	SCIN	scinderin	ENST00000445618	SCIN-201	2.583
ENSG00000156398	SFXN2	sideroflexin 2	ENST00000459894	SFXN2-002	-2.551
ENSG00000067066	SP100	SP100 nuclear antigen	ENST00000409341	SP100-006	2.857
ENSG00000067066	SP100	SP100 nuclear antigen	ENST00000494901	SP100-007	2.524
ENSG00000067066	SP100	SP100 nuclear antigen	ENST00000413284	SP100-016	2.444
ENSG00000102524	TNFSF13B	tumor necrosis factor (ligand) superfamily, member 13b	ENST00000375887	TNFSF13B-001	4.924
ENSG00000102524	TNFSF13B	tumor necrosis factor (ligand) superfamily, member 13b	ENST00000493765	TNFSF13B-004	2.882

HRF

Ensembl.Gene.ID	Assoc.Gene.Name	Description	Ensembl.Transcript.ID	Associated.Transcript.Name	log2 fold change
ENSG00000128512	DOCK4	dedicator of cytokinesis 4	ENST00000423057	DOCK4-007	2.068
ENSG00000160460	SPTBN4	spectrin, beta, non-erythrocytic 4	ENST00000352632	SPTBN4-203	-2.735
